# Supplementary material for: Fluorescent Pyranoindole Congeners: Synthesis and Photophysical Properties of Pyrano[3,2-f], [2,3-g], [2,3-f], and [2,3-e]Indoles
Source: Molecules. 2022 Dec 13;27(24):8867. doi: 10.3390/molecules27248867 (PMC9783255; doi:10.3390/molecules27248867)
Supplement: Supplementary file 1 [file molecules-27-08867-s001.zip › molecules-2084715-supplementary.pdf]

Fluorescent pyranoindole congeners: synthesis and photophysical properties of pyrano[3,2-*f*], [2,3-*g*], [2,3-*f*], and [2,3-*e*]indoles

Ainur D. Sharapov, Ramil F. Fatykhov, Igor A. Khalymbadzha, Maria I. Valieva, Igor L. Nikonov, Olga S. Taniya, Dmitry S. Kopchuk, Grigory V. Zyryanov, Alexander S. Novikov and Oleg N. Chupakhin

Supporting Information

Table of Contents

Experimental Section 5

General Information: 5

Synthesis and characterization of pyranoindole compounds 5

Table S1. Yields, NMR data, elemental analysis and melting points for compounds **2**, **3**, **6**-**10**, **12** and **14**.. 6

Figure S1. ^{1}H NMR spectrum of **2**. 13

Figure S2. ^{13}C NMR spectrum of **2**. 13

Figure S3. ^{1}H NMR spectrum of **3**. 14

Figure S4. ^{13}C NMR spectrum of **3**. 14

Figure S5. ^{1}H NMR spectrum of **6a**. 15

Figure S6. ^{13}C NMR spectrum of **6a**. 15

Figure S7. ^{1}H NMR spectrum of **6b**. 16

Figure S8. ^{13}C NMR spectrum of **6b**. 16

Figure S9. ^{1}H NMR spectrum of **7a**. 17

Figure S10. ^{13}C NMR spectrum of **7a** 17

Figure S11. ^{1}H NMR spectrum of **8a**. 18

Figure S12. ^{13}C NMR spectrum of **8a**. 18

Figure S13. ^{1}H NMR spectrum of **7b**. 19

Figure S14. ^{13}C NMR spectrum of **7b**. 19

Figure S15. ^{1}H NMR spectrum of **8b**. 20

Figure S16. ^{13}C NMR spectrum of **8b**. 20

Figure S17. ^{1}H NMR spectrum of **7c**. 21

Figure S18. ^{13}C NMR spectrum of **7c**. 21

Figure S19. ^{1}H NMR spectrum of **8c**. 22

Figure S20. ^{13}C NMR spectrum of **8c**. 22

Figure S21. ^{1}H NMR spectrum of **7d**. 23

Figure S22. ^{13}C NMR spectrum of **7d**. 23

Figure S23. ^{1}H NMR spectrum of **8d**. 24

Figure S24. ^{13}C NMR spectrum of **8d**. 24

Figure S25. ^{1}H NMR spectrum of **7e**. 25

Figure S26. ^{13}C NMR spectrum of **7e**. 25

Figure S27. ^{1}H NMR spectrum of **8e**. 26
Figure S28. ^{13}C NMR spectrum of **8e**. 26
Figure S29. ^{1}H NMR spectrum of **7f**. 27
Figure S30. ^{13}C NMR spectrum of **7f**. 27
Figure S31. ^{1}H NMR spectrum of **8f**. 28
Figure S32. ^{13}C NMR spectrum of **8f**. 28
Figure S33. ^{1}H NMR spectrum of **7g**. 29
Figure S34. ^{13}C NMR spectrum of **7g**. 29
Figure S35. ^{1}H NMR spectrum of **8g**. 30
Figure S36. ^{13}C NMR spectrum of **8g**. 30
Figure S37. ^{1}H NMR spectrum of **7h**. 31
Figure S38. ^{13}C NMR spectrum of **7h**. 31
Figure S39. ^{1}H NMR spectrum of **7i**. 32
Figure S40. ^{13}C NMR spectrum of **7i**. 32
Figure S41. ^{1}H NMR spectrum of **9a**. 33
Figure S42. ^{13}C NMR spectrum of **9a**. 33
Figure S43. ^{1}H NMR spectrum of **9b**. 34
Figure S44. ^{13}C NMR spectrum of **9b**. 34
Figure S45. ^{1}H NMR spectrum of **9c**. 35
Figure S46. ^{13}C NMR spectrum of **9c**. 35
Figure S47. ^{1}H NMR spectrum of **9d**. 36
Figure S48. ^{13}C NMR spectrum of **9d**. 36
Figure S49. ^{1}H NMR spectrum of **10**. 37
Figure S50. ^{13}C NMR spectrum of **10**. 38
Figure S51. ^{1}H NMR spectrum of 2,3-diphenyl-6-methoxyindole **13**. 38
Figure S52. ^{1}H NMR spectrum of 2,3-diphenyl-6-methoxyindole **13**. 38
Photophysical data 39
Materials and Methods 39
Equipment, measurements and characterization methods 39
Photoluminescence Absolute quantum yield (PLQY) measurement 39
Solvatochromic behavior of pyranoindole compounds 39
Table S2. Orientation polarizability for solvents (Δf), absorption and fluorescence emission maxima (λ_{abs}, λ_{em}, nm) and Stokes shift (nm, cm^{-1}) of **2** in different solvents. 39
Table S3. Orientation polarizability for solvents (Δf), absorption and fluorescence emission maxima (λ_{abs}, λ_{em}, nm) and Stokes shift (nm, cm^{-1}) of **6b** in different solvents. 39
Table S4. Orientation polarizability for solvents (Δf), absorption and fluorescence emission maxima (λ_{abs}, λ_{em}, nm) and Stokes shift (nm, cm^{-1}) of **7a** in different solvents. 39

Table S5. Orientation polarizability for solvents (Δf), absorption and fluorescence emission maxima (λ_{abs}, λ_{em}, nm) and Stokes shift (nm, cm^{-1}) of **7c** in different solvents. 40

Table S6. Orientation polarizability for solvents (Δf), absorption and fluorescence emission maxima (λ_{abs}, λ_{em}, nm) and Stokes shift (nm, cm^{-1}) of **7e** in different solvents. 40

Figure S53. Normalized fluorescence spectra of 7e in different solvents (C = 10^{-5} M^{-1}). 40

Table S7. Orientation polarizability for solvents (Δf), absorption and fluorescence emission maxima (λ_{abs}, λ_{em}, nm) and Stokes shift (nm, cm^{-1}) of **8a** in different solvents. 40

Table S8. Orientation polarizability for solvents (Δf), absorption and fluorescence emission maxima (λ_{abs}, λ_{em}, nm) and Stokes shift (nm, cm^{-1}) of **8c** in different solvents. 41

Table S9. Orientation polarizability for solvents (Δf), absorption and fluorescence emission maxima (λ_{abs}, λ_{em}, nm) and Stokes shift (nm, cm^{-1}) of **8d** in different solvents. 41

Figure S54. Fluorescence photograph of pyranoindole **7e** (1 mM, excitation with 365 nm Hg lamp) in different solvents (left to right: *n*-heptane, toluene, tetrahydrofuran, dichloromethane, DMSO, acetonitrile, methanol). 41

Table S10. Orientation polarizability for solvents (Δf), absorption and fluorescence emission maxima (λ_{abs}, λ_{em}, nm) and Stokes shift (nm, cm^{-1}) of **8e** in different solvents. 41

Figure S55. Normalized fluorescence spectra of 8e in different solvents (C = 10^{-5} M^{-1}). 42

Table S11. Orientation polarizability for solvents (Δf), absorption and fluorescence emission maxima (λ_{abs}, λ_{em}, nm) and Stokes shift (nm, cm^{-1}) of **8f** in different solvents. 42

Table S12. Orientation polarizability for solvents (Δf), absorption and fluorescence emission maxima (λ_{abs}, λ_{em}, nm) and Stokes shift (nm, cm^{-1}) of **8g** in different solvents. 42

Table S13. Orientation polarizability for solvents (Δf), absorption and fluorescence emission maxima (λ_{abs}, λ_{em}, nm) and Stokes shift (nm, cm^{-1}) of **9a** in different solvents. 42

Table S14. Orientation polarizability for solvents (Δf), absorption and fluorescence emission maxima (λ_{abs}, λ_{em}, nm) and Stokes shift (nm, cm^{-1}) of **9b** in different solvents. 43

Table S15. Orientation polarizability for solvents (Δf), absorption and fluorescence emission maxima (λ_{abs}, λ_{em}, nm) and Stokes shift (nm, cm^{-1}) of **10** in different solvents. 43

Table S16. Orientation polarizability for solvents (Δf), absorption and fluorescence emission maxima (λ_{abs}, λ_{em}, nm) and Stokes shift (nm, cm^{-1}) of **12** in different solvents. 43

Table S17. Lippert-Mataga plot for compounds **2**, **6**, **7**, **8**, **9**, **10**, and **12**. 43

Table S18. The photophysical properties of compounds in different solvents. 44

Table S19. Fluorescence lifetime of probe **8c**, **8g**, **9a** (C = 2×10^{-6} M) in MeOH. 45

Table S20. Energies of HOMO and LUMO, and energy gap for compounds **7** and **8**. 45

Figure S56. Calculated UV-Vis spectra for optimized equilibrium model structure **7c** (CAM-B3LYP/6-31+G* level of theory). 46

Figure S57. Calculated UV-Vis spectra for optimized equilibrium model structure 8c (CAM-B3LYP/6-31+G* level of theory). 46

Figure S58. Molecular orbital and energy levels of **7a**-**8a**, **7c**-**8c**, **7d**-**8d**, **7e**-**8e**, **7g**-**8g** pyranoindole fluorophores, calculated at the CAM-B3LYP/6-31+G* level of theory. 47

Figure S59. Visualization of HOMOs and LUMOs responsible for observed UV-Vis spectra in optimized equilibrium model structure 7c (CAM-B3LYP/6-31+G* level of theory). 48

Figure S60. Visualization of HOMOs and LUMOs responsible for observed UV-Vis spectra in optimized equilibrium model structure 8c (CAM-B3LYP/6-31+G* level of theory). 48

Table S21. Optimized structures in S_0 in gas phase and visualization of molecular electrostatic potential distribution. 49

Table S22. Calculated indexes related to hole-electron distribution in model structures......................... 51

Experimental Section
General Information:

All reagents were purchased from commercial sources and used without further purification. Silica gel 60 (Kieselgel 60, 230-400 mesh) was used for the column chromatography. NMR spectra were recorded on a Bruker Avance-400 spectrometer, 298 K, digital resolution ± 0.01 ppm, using TMS as internal standard and (Bruker Avance-600 spectrometer, 298 K, digital resolution ± 0.01 ppm, using residue signals of solution as internal standard). UV–Vis spectra were recorded on Lambda 45 spectrophotometer (Perkin Elmer). Luminescence spectra were recorded on a Horiba-Fluoromax-4 spectrofluorimeter equipped with integrated sphere.

Synthesis and characterization of pyranoindole compounds

*Synthesis of ethyl 9-methyl-5-oxo-1,2,3,4,5,10-hexahydroisochromeno[3,4-f]indole-8-carboxylate **2***

A mixture of ethyl 5-hydroxy-2-methyl-1*H*-indole-3-carboxylate **1** (5 mmol, 1095 mg, 1.0 equiv), ethyl 2-oxocyclohexane-1-carboxylate (5.5 mmol, 936 mg, 1.1 equiv), and MsOH (0,5 mmol, 48 mg, 0.1 equiv) was stirred at ambient temperature for 4 hours. The reaction mixture was crystallized from DMF to obtain pure **2** (992 mg, 61% yield).

*Synthesis of 9-methyl-2,3,4,10-tetrahydroisochromeno[3,4-f]indol-5(1H)-one **3***

3-Carbethoxyindole **2** (1 mmol, 325 mg) was added to a solution of 0.2 mL H_2SO_4 in 1.5 mL AcOH. The reaction mixture was stirred under heating for 36 hours, poured into water and the precipitate was filtered off. The resulting precipitate was purified by flash chromatography (chloroform/silica gel) affording pyranoindole **3** (124 mg, 49% yield).

*Synthesis of pyrano pyrano[2,3-e]indoles **6***

A mixture of 2,3-diphenyl-1*H*-indol-4-ol **4** (856 mg, 3.0 mmol, 1.0 equiv), 2-ketoester (3.3 mmol, 1.1 equiv), and MsOH (0.3 mmol, 29 mg, 0.1 equiv) were stirred at 70°C for 5 hours. After completion of the reaction, the resulting mixture was recrystallized from DMF providing **6**.

*Synthesis of pyrano[3,2-f] and [2,3-g]indoles **7** and **8***

A mixture of 2,3-diphenyl-1*H*-indol-6-ol **5** (856 mg, 3.0 mmol, 1.0 equiv), 2-ketoester (3.3 mmol, 1.1 equiv), and MsOH (0.3 mmol, 29 mg, 0.1 equiv) were stirred at ambient temperature (for compounds) or at 50°C (for compounds **7,8a** and **7,8b**) for 5 hours. After completion of the reaction, the crude mixture was separated to obtain isomeric indoles **7** and **8** using silica gel chromatography (chloroform-hexane (1:1) as eluent).

*Alkylation of pyrano[3,2-f]indol-2(8H)-ones **7***

To a solution of pyrano[3,2-*f*]indol-2(8*H*)-one **7** (1 mmol) and ethyl iodide (1.2 mmol, 187 mg) or benzyl bromide (1.2 mmol, 171 mg) in DMF a suspension of sodium hydride (1.2 mmol, 60%, 48 mg) was added. The reaction mixture is stirred for 8 hours, then water was added and the pH of

the resulting mixture is adjusted to 6 by adding acetic acid. The resulting precipitate was filtered off, washed with hexane, and recrystallized from ethanol to obtain pure **9**.

Aromatization of 2,3-diphenyl-8,9,10,11-tetrahydroisochromeno[3,4-g]indol-7(1H)-one

A solution of 2,3-diphenyl-8,9,10,11-tetrahydroisochromeno[3,4-*g*]indol-7(1*H*)-one **8g** (1 mmol, 392 mg) and DDQ (3 mmol, 681 mg) was heated under reflux for 6 hours. The reaction mixture was diluted with methylene chloride and the resulting solution was passed through a layer of aluminum oxide. The eluate was evaporated and the resulting solid was recrystallized from ethyl alcohol yielding 162 mg, 42% 2,3-diphenylisochromeno[3,4-*g*]indol-7(1*H*)-one **10**.

Table S1. Yields, NMR data, elemental analysis and melting points for compounds **2**, **3**, **6-10**, **12** and **14**.

Cmpd	Structure	Characterization
**2**	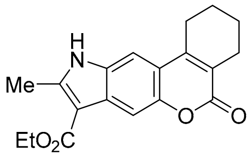	Off-white solid, m.p. > 300 °C. ^{1}H NMR (400 MHz, DMSO-d_6) δ 12.00 (s, 1H), 7.62 (s, 1H), 7.43 (s, 1H), 4.28 (q, *J* = 7.1 Hz, 2H), 2.75 (br s, 2H); 2.66 (s, 3H), 2.38 (br s, 2H), 1.68–1.78 (m, 4H), 1.37 (t, 3H). ^{13}C{^{1}H} NMR (101 MHz, DMSO-d_6) δ 164.6, 161.0, 148.6, 147.3, 146.9, 131.8, 128.5, 120.2, 114.8, 105.8, 104.9, 102.7, 59.0, 24.6, 23.7, 21.2, 20.9, 14.4, 13.9. Anal. Calcd for $C_{19}H_{19}NO_4$: C, 70.14; H, 5.89; N, 4.31. Found: C, 69.97; H, 5,02; N, 4.19.
**3**	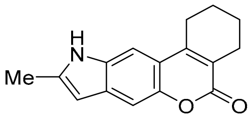	Light yellow solid with m.p. = 279–281 °C. ^{1}H NMR (400 MHz, DMSO-d_6) δ 11.19 (s, 1H), 7.51 (s, 1H), 7.33 (s, 1H), 6.21 (s, 1H), 2.84–2.87 (m, 2H), 2.43 (m, 5H), 1.72–1.84 (m, 4H). ^{13}C{^{1}H} NMR (101 MHz, DMSO-d_6) δ 161.4, 147.9, 145.7, 140.8, 133.4, 130.5, 119.2, 113.6, 104.1, 104.0, 99.3, 24.8, 23.8, 21.4, 21.1, 13.6. Anal. Calcd for $C_{16}H_{15}NO_2$: C, 75.87; H, 5.97; N, 5.53. Found: 75.79; H, 6.08; N, 5.35.
**6a**	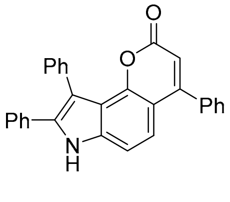	Yield 1406 mg, 68%. Beige solid with m.p. = 254-255 °C. ^{1}H NMR (400 MHz, DMSO-d_6) δ 12.18 (s, 1H), 7.53–7.59 (m, 5H), 7.28–7.43 (m, 11H), 7.15 (d, *J* = 8.7 Hz, 1H), 6.15 (s, 1H). ^{13}C{^{1}H} NMR (151 MHz, DMSO-d_6) δ 159.7, 156.9, 149.4, 138.7, 136.2, 135.7, 134.8, 131.6, 131.1 (2C), 129.4, 128.8 (2C), 128.50 (2C), 128.47 (2C), 128.3 (2C), 127.9 (2C), 127.8, 126.7, 120.0, 115.5, 114.1, 110.4, 110.2, 109.0. Anal. Calcd for $C_{29}H_{19}NO_2$: C, 84.24; H, 4.63; N, 3.39. Found: C, 84.07; H, 5.74; N, 3.19.

Cmpd	Structure	Characterization
**6b**	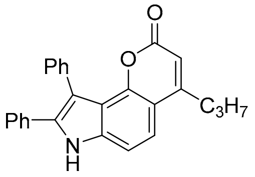	Yield 1195 mg, 63%. Light gray solid with m.p. = 252 °C. ^{1}H NMR (600 MHz, DMSO-d$_6$) δ 12.13 (s, 1H), 7.54 (d, *J* = 8.7 Hz, 1H), 7.43 (d, *J* = 8.7 Hz, 1H), 7.27–7.38 (m, 10H), 6.10 (s, 1H), 2.78–2.81 (m, 2H), 1.68–1.71 (m, 2H), 0.98–1.00 (m, 3H). ^{13}C{^{1}H} NMR (151 MHz, DMSO-d$_6$) δ 160.0, 158.0, 149.0, 138.5, 135.4, 134.8, 131.7, 131.1 (2C), 128.4 (2C), 128.3 (2C), 127.8 (2C), 127.7, 126.6, 118.1, 115.4, 114.0, 110.6, 109.4, 108.8, 33.8, 21.6, 13.7. Anal. Calcd for $C_{26}H_{21}NO_2$: C, 82.30; H, 5.58; N, 3.69. Found: C, 82.37; H, 5.66; N, 3.86.
**7a**	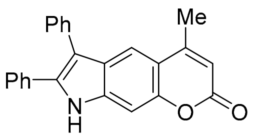	Yield 931 mg, 53%. Yellow solid with m.p. = 270°C. ^{1}H NMR (400 MHz, DMSO-d$_6$) δ 11.93 (s, 1H), 7.76 (s, 1H), 7.33–7.48 (m, 11H), 6.23 (s, 1H), 2.44 (s, 3H). ^{13}C{^{1}H} NMR (101 MHz, DMSO-d$_6$) δ 160.4, 154.0, 149.7, 137.9, 136.2, 134.3, 131.7, 129.8 (2C), 128.9 (2C), 128.6 (2C), 128.1 (2C), 128.0, 126.6, 125.7, 114.9, 113.8, 113.7, 111.1, 97.6, 18.5. Anal. Calcd for $C_{24}H_{17}NO_2$: C, 82.03; H, 4.88; N, 3.99. Found: C, 81.92; H, 4.98; N, 3.84.
**8a**	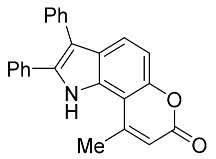	Yield 614 mg, 32%. Yellow solid with m.p. >300°C. ^{1}H NMR (400 MHz, DMSO-d$_6$) δ 10.82 (s, 1H), 7.68 (d, *J* = 8.6 Hz, 1H), 7.31–7.49 (m, 10H), 7.15 (d, *J* = 8.6 Hz, 1H), 6.35 (s, 1H), 2.93 (s, 3H). ^{13}C{^{1}H} NMR (101 MHz, DMSO-d$_6$) δ 159.9, 153.1, 151.1, 136.0, 133.9, 131.8, 130.9, 129.8 (2C), 129.2 (2C), 128.7 (2C), 128.3 (2C), 127.8, 126.6, 125.7, 123.0, 114.8, 112.7, 110.2, 106.6, 22.1. Anal. Calcd for $C_{24}H_{17}NO_2$: C, 82.03; H, 4.88; N, 3.99. Found: C, 82.08; H, 4.75; N, 4.07.
**7b**	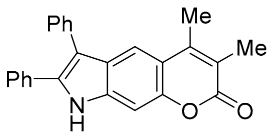	Yield 767 mg, 42%. Beige solid with m.p. > 300 °C. ^{1}H NMR (400 MHz, DMSO-d$_6$) δ 11.74 (s, 1H), 7.74 (s, 1H), 7.30–7.48 (m, 11H), 2.39 (s, 3H), 2.11 (s, 3H). ^{13}C{^{1}H} NMR (101 MHz, DMSO-d$_6$) δ 161.3, 148.2, 147.0, 137.0, 135.8, 134.4, 131.7, 129.6 (2C), 128.6 (2C), 128.3 (2C), 127.9 (2C), 127.7, 126.3, 125.6, 117.3, 114.3, 114.1, 113.6, 97.1, 14.8, 12.9. Anal. Calcd for $C_{25}H_{19}NO_2$: C, 82.17; H, 5.24; N, 3.83. Found: C, 82.00; H, 5.37; N, 3.88.
**8b**	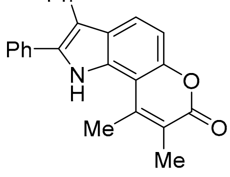	Yield 730 mg, 40%. Beige solid with m.p. > 300 °C. ^{1}H NMR (400 MHz, DMSO-d$_6$) δ 10.84 (s, 1H), 7.62 (d, *J* = 8.6 Hz, 1H), 7.31–7.48 (m, 10H), 7.12 (d, *J* = 8.6 Hz, 1H), 2.88 (s, 3H), 2.17 (s, 3H). ^{13}C{^{1}H} NMR (101 MHz, DMSO-d$_6$) δ 160.9, 149.1, 146.7, 135.7, 134.1, 131.9, 130.7, 129.9, 129.2, 128.7, 128.3 (2C), 127.7 (2C), 126.5 (2C), 125.9 (2C), 121.7, 119.1, 114.7, 110.0, 107.4, 18.7, 13.0. Anal. Calcd for $C_{25}H_{19}NO_2$: C, 82.17; H, 5.24; N, 3.83. Found: C, 82.29; H, 5.34; N, 3.70.

Cmpd	Structure	Characterization
**7c**	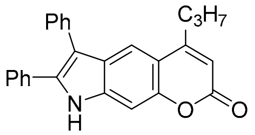	Yield 1176 mg, 62%. Yellow needles with m.p. > 300°C. ^{1}H NMR (400 MHz, DMSO-d_6) δ 11.82 (s, 1H), 7.77 (s, 1H) 7.32–7.48 (m, 11H), 6.15 (s, 1H), 2.76–2.79 (m, 2H), 1.64–1.74 (m, 2H), 0.95–0.99 (m, 3H). ^{13}C{^{1}H} NMR (101 MHz, DMSO-d_6) δ 160.3, 156.9, 149.8, 137.7, 136.2, 134.2, 131.6, 129.6 (2C), 128.6 (2C), 128.3 (2C), 127.9 (2C), 127.8, 126.4, 125.6, 114.2, 113.6, 112.9, 109.9, 97.7, 32.8, 20.8, 13.4. Anal. Calcd for $C_{26}H_{21}NO_2$: C, 82.30; H, 5.58; N, 3.69. Found: C, 82.22; H, 5.69; N, 3.46.
**8c**	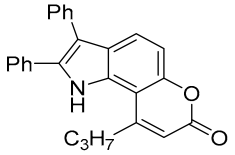	Yield 284 mg, 15%. Beige solid with m.p. > 300 °C. ^{1}H NMR (600 MHz, DMSO-d_6) δ 10.87 (s, 1H), 7.70 (d, *J* = 8.6 Hz, 1H), 7.31–7.44 (m, 10H), 7.18 (d, *J* = 8.6 Hz, 1H), 6.31 (s, 1H), 3.32–3.35 (m, 2H), 1.80–1.82 (m, 2H), 1.09–1.12 (m, 3H). ^{13}C{^{1}H} NMR (151 MHz, DMSO-d_6) δ 160.5, 156.9, 151.9, 136.6, 134.4, 132.3, 130.4 (2C), 130.1, 129.6 (2C), 129.2 (2C), 128.9 (2C), 128.3, 127.1, 126.5, 123.5, 115.3, 112.1, 111.0, 106.7, 35.8, 20.6, 13.9. Anal. Calcd for $C_{26}H_{21}NO_2$: C, 82.30; H, 5.58; N, 3.69. Found: C, 82.36; H, 5.60; N, 3.74.
**7d**	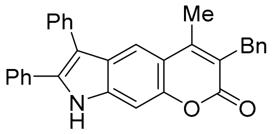	Yield 1324 mg, 60%. Yellow solid with m.p. > 275 °C. ^{1}H NMR (400 MHz, DMSO-d_6) δ 11.79 (s, 1H), 7.80 (s, 1H), 7.17–7.48 (m, 16H), 3.99 (s, 2H), 2.46 (s, 3H). ^{13}C{^{1}H} NMR (101 MHz, DMSO-d_6) δ 161.3, 148.6, 148.5, 139.4, 137.3, 136.0, 134.3, 131.6, 129.6 (2C), 128.6 (2C), 128.3 (2C), 128.2 (2C), 127.9 (2C), 127.8 (2C), 127.7, 126.4, 125.7, 125.7, 120.6, 114.7, 114.2, 113.7, 97.1, 32.2, 15.2. Anal. Calcd for $C_{31}H_{23}NO_2$: C, 84.33; H, 5.25; N, 3.17. Found: C, 84.16; H, 5.23; N, 3.36.
**8d**	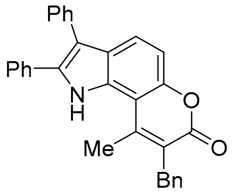	Yield 441 mg, 20%. Yellow solid with m.p. = 275 °C. ^{1}H NMR (400 MHz, DMSO-d_6) δ 10.84 (s, 1H), 7.66 (d, *J* = 8.6, 1H), 7.18–7.48 (m, 15H), 7.16 (d, *J* = 8.6 Hz, 1H), 4.07 (s, 2H), 2.95 (s, 3H). ^{13}C{^{1}H} NMR (101 MHz, DMSO-d_6) δ 161.0, 149.5, 148.5, 139.4, 135.8, 134.0, 131.8, 130.8, 129.9 (2C), 129.1 (2C), 128.70 (2C), 128.66, 128.4 (2C), 128.3 (2C), 128.0 (2C), 127.7, 126.5, 126.0, 126.0, 122.3, 122.2, 114.8, 110.1, 107.5, 31.9, 19.0. Anal. Calcd for $C_{31}H_{23}NO_2$: C, 84.33; H, 5.25; N, 3.17. Found: C, 84.23; H, 5.18; N, 3.22.
**7e**	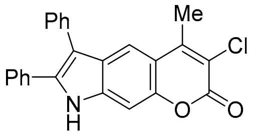	Yield 1292 mg, 67%. Yellow solid with m.p. > 220 °C dec. ^{1}H NMR (400 MHz, DMSO-d_6) δ 11.98 (s, 1H), 7.77, (s, 1H) 7.33–7.46 (m, 11H), 2.56 (s, 3H). ^{13}C{^{1}H} NMR (101 MHz, DMSO-d_6) δ 156.6, 149.4, 147.6, 137.7, 136.6, 134.2, 131.5, 129.8 (2C), 128.9 (2C), 128.6 (2C), 128.2 (2C), 128.1, 126.7, 126.0, 115.8, 115.3, 113.7, 113.4, 97.6, 16.3. Anal. Calcd for $C_{24}H_{16}ClNO_2$: C, 74.71; H, 4.18; N, 3.63. Found: C, 74.89; H, 4.05; N, 3.39.

Cmpd	Structure	Characterization
**8e**	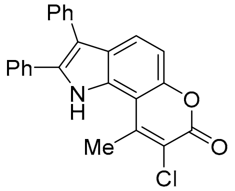	Yield 289 mg, 15%. Yellow solid with m.p. > 230 °C. ^{1}H NMR (400 MHz, DMSO-d_6) δ 11.01 (s, 1H), 7.72 (d, *J* = 8.7 Hz, 1H), 7.47–7.49 (m, 2H), 7.31–7.43 (m, 8H), 7.19 (d, *J* = 8.7 Hz, 1H), 3.08 (s, 3H). ^{13}C{^{1}H} NMR (101 MHz, DMSO-d_6) δ 156.2, 148.7, 148.4, 136.3, 133.8, 131.6, 130.4, 129.9 (2C), 129.3 (2C), 128.7 (2C), 128.3 (2C), 127.9, 126.6, 126.2, 123.2, 117.5, 114.9, 110.0, 106.7, 19.7. Anal. Calcd for $C_{24}H_{16}ClNO_2$: C, 74.71; H, 4.18; N, 3.63. Found: C, 74.89; H, 4.04; N, 3.34.
**7f**	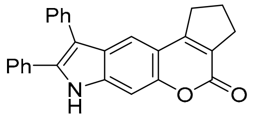	Yield 1076 mg, 57%. Yellow solid with m.p. > 300 °C. ^{1}H NMR (600 MHz, DMSO-d_6) δ 11.83 (s, 1H), 7.55 (br s, 1H), 7.32–7.47 (m, 11H), 3.07–3.11 (m, 2H), 2.75–2.78 (m, 2H), 2.07–2.12 (m, 2H). ^{13}C{^{1}H} NMR (151 MHz, DMSO-d_6) δ 159.4, 157.0, 150.2, 137.4, 136.0, 134.4, 131.7, 129.6 (2C), 128.6 (2C), 128.4 (2C), 127.9 (2C), 127.8, 126.4, 125.6, 123.0, 114.5, 113.5, 112.5, 97.4, 31.7, 30.1, 21.8. Anal. Calcd for $C_{26}H_{19}NO_2$: C, 82.74; H, 5.07; N, 3.71. Found: C, 82.80; H, 5.03; N, 3.81.
**8f**	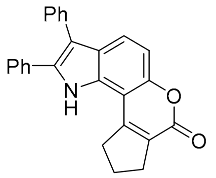	Yield 528 mg, 28%. Beige solid with m.p. > 300°C. ^{1}H NMR (600 MHz, DMSO-d_6) δ 10.90 (s, 1H), 7.64 (d, *J* = 8.7 Hz, 1H), 7.30–7.41 (m, 2H), 7.45–7.47 (m, 8H), 7.16 (d, *J* = 8.7 Hz, 1H), 3.61–3.63 (m, 2H), 2.77–2.80 (m, 2H), 2.16–2.21 (m, 2H). ^{13}C{^{1}H} NMR (151 MHz, DMSO-d_6) δ 159.1, 154.4, 151.2, 136.0, 134.1, 131.8, 130.5, 129.8 (2C), 129.3 (2C), 128.7 (2C), 128.3 (2C), 127.8, 126.5, 125.2, 125.0, 121.9, 114.8, 109.9, 105.0, 34.2, 29.6, 22.1. Anal. Calcd for $C_{26}H_{19}NO_2$: C, 82.74; H, 5.07; N, 3.71. Found: C, 82.69; H, 4.97; N, 3.89.
**7g**	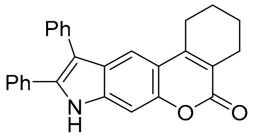	Yield 665 mg, 34%. White solid with m.p. > 290 °C. ^{1}H NMR (600 MHz, DMSO-d_6) δ 11.86 (s, 1H), 7.69 (s, 1H), 7.32–7.48 (m, 11H), 2.80 (br s, 2H), 2.44 (br s, 2H), 1.68–1.78 (m, 4H). ^{13}C{^{1}H} NMR (151 MHz, DMSO-d_6) δ 161.0, 148.2, 147.8, 136.9, 135.8, 134.5, 131.8, 129.7 (2C), 128.6 (2C), 128.4 (2C), 127.9 (2C), 127.7, 126.3, 125.5, 119.0, 113.8, 113.5, 112.9, 97.2, 24.6, 23.6, 21.2, 20.9. Anal. Calcd for $C_{27}H_{21}NO_2$: C, 82.84; H, 5.41; N, 3.58. Found: C, 82.93; H, 5.43; N, 3.39.

Cmpd	Structure	Characterization
**8g**	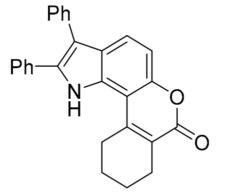	Yield 626 mg, 32%. Cream solid with m.p. > 300 °C.^{1}H NMR (400 MHz, DMSO-d_6) δ 10.75 (s, 1H), 7.62 (d, J = 8.6 Hz, 1H), 7.45–7.47 (m, 2H), 7.29–7.42 (m, 8H), 7.12 (d, J = 8.6 Hz, 1H), 3.40 (br s, 2H), 2.48 (br s, 2H), 1.75–1.87 (m, 4H). ^{13}C{^{1}H} NMR (101 MHz, DMSO-d_6) δ 160.5, 149.2, 148.0, 135.7, 134.1, 131.9, 130.3, 129.9 (2C), 129.3 (2C), 128.7 (2C), 128.2 (2C), 127.7, 126.5, 125.7, 121.6, 120.5, 114.6, 110.1, 106.9, 28.2, 24.1, 21.3, 20.9. Anal. Calcd for $C_{27}H_{21}NO_2$: C, 82.84; H, 5.41; N, 3.58. Found: C, 82.93; H, 5.32; N, 3.33.
**7h**	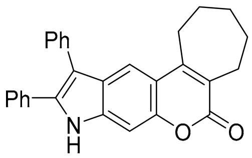	Yield 1379 mg, 68%. Off-white solid, m.p. > 300 °C. ^{1}H NMR (600 MHz, DMF-d_7) δ 11.98 (s, 1H), 8.07 (s, 1H), 7.61–7.63 (m, 2H), 7.39–7.56 (m, 8H), 3.10–3.12 (m, 2H), 2.93–2.95 (m, 2H), 1.91–1.93 (m, 2H), 1.70–1.71 (m ,2H), 1.60–1.62 (m, 2H). ^{13}C{^{1}H} NMR (151 MHz, DMF-d_7) δ 156.0, 150.8, 139.0, 137.5, 136.1, 133.4, 131.3 (2C), 130.0 (2C), 129.7 (2C), 129.4 (2C), 129.0, 127.8, 127.4, 125.5, 115.5, 115.4 (2C), 115.1, 98.9, 32.8, 28.9, 27.5, 26.9, 26.3. Anal. Calcd for $C_{28}H_{23}NO_2$: C, 82.94; H, 5.72; N, 3.45. Found: C, 82.85; H, 5.80; N, 3.31.
**7i**	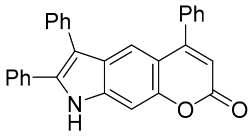	Yellow solid, m.p. > 300 °C. Yield 1199 mg, 58%. ^{1}H NMR (400 MHz, DMSO-d_6) δ 12.01 (s, 1H), 7.26–7.59 (m, 17H), 6.23 (s, 1H). ^{13}C{^{1}H} NMR (101 MHz, DMSO-d_6) δ 160.3, 156.3, 150.3, 137.9, 136.3, 135.6, 134.1, 131.5, 129.6 (3C), 128.7 (4C), 128.6 (2C), 128.5 (2C), 128.1 (3C), 126.7, 125.8, 117.1, 113.8, 112.6, 111.2, 98.1. Anal. Calcd for $C_{29}H_{19}NO_2$: C, 84.24; H, 4.63; N, 3.39. Found: C, 84.01; H, 4,85; N, 3.23.
**9a**	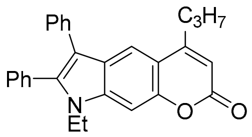	Yield 354 mg, 87%. Cream solid with m.p. > 200-201 °C. ^{1}H NMR (600 MHz, DMSO-d_6) δ 7.92 (s, 1H), 7.67 (s, 1H), 7.46–7.47 (m, 3H), 7.38–7.40 (m, 2H), 7.29–7.32 (m, 2H), 7.26–7.28 (m, 2H), 7.19–7.21 (m, 1H), 6.19 (s, 1H), 4.12–4.16 (m, 2H), 2.80–2.82 (m, 2H), 1.67–1.73 (m, 2H), 1.13–1.16 (m, 3H), 0.96–0.98 (m, 3H). ^{13}C{^{1}H} NMR (151 MHz, DMSO-d_6) δ 160.5, 157.0, 149.8, 139.1, 137.3, 133.8, 130.9, 130.7 (2C), 129.2 (2C), 128.6, 128.5 (2C), 128.4 (2C), 126.0, 123.9, 114.9, 114.7, 113.1, 110.2, 97.3, 38.5, 32.9, 21.0, 14.7, 13.6. Anal. Calcd for $C_{28}H_{25}NO_2$: C, 82.53; H, 6.18; N, 3.44. Found: C, 83.85; H, 6.40; N, 3.24.

Cmpd	Structure	Characterization
**9b**	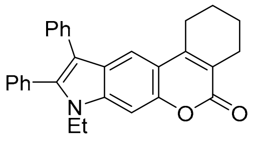	Yield 294 mg, 70%. Beige solid with m.p. > 205-207 °C. ^{1}H NMR (400 MHz, DMSO-d_6) δ 7.86 (s, 1H), 7.65 (s, 1H), 7.12–7.49 (m, 10H), 4.11–4.16 (m, 2H), 2.86 (br s, 2H), 2.45 (br s, 2H), 1.72–1.79 (m, 4H), 1.12–1.15 (s, 3H). ^{13}C{^{1}H} NMR (101 MHz, DMSO-d_6) δ 161.1, 148.2, 148.0, 138.8, 136.6, 134.0, 131.0, 130.8 (2C), 129.3 (2C), 128.7, 128.6 (2C), 128.4 (2C), 125.9, 123.8, 119.3, 114.8, 114.1, 113.4, 96.8, 38.5, 24.7, 23.7, 21.3, 21.0, 14.8. Anal. Calcd for $C_{29}H_{25}NO_2$: C, 83.03; H, 6.01; N, 3.34. Found: C, 82.98; H, 6.10; N, 3.56.
**9c**	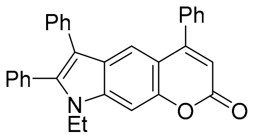	Yield 397 mg, 90%. Yellow crystals with m.p. > 210 °C. ^{1}H NMR (600 MHz, DMSO-d_6) δ 7.81 (s, 1H), 7.70 (s, 1H), 7.60–7.62 (m, 2H), 7.55–7.56 (m, 3H), 7.46–7.47 (m, 3H), 7.39–7.40 (m, 2H), 7.19–7.22 (m, 2H), 7.11–7.15 (m, 3H), 6.26 (s, 1H), 4.18 (q, *J* = 7.1 Hz, 2H), 1.16 (t, *J* = 7.1 Hz, 3H). ^{13}C{^{1}H} NMR (151 MHz, DMSO-d_6) δ 160.4, 156.2, 150.3, 139.2, 137.4, 135.5, 133.6, 130.8, 130.7 (2C), 129.6, 129.1 (2C), 128.8, 128.7 (2C), 128.6 (2C), 128.5 (2C), 128.3 (2C), 126.1, 124.0, 117.4, 115.0, 112.7, 111.3, 97.6, 38.6, 14.8. Anal. Calcd for $C_{31}H_{23}NO_2$: C, 84.33; H, 5.25; N, 3.17. Found: C, 84.18; H, 5.36; N, 3.02.
**9d**	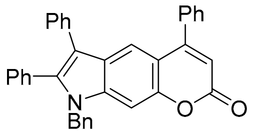	Yield 448 mg, 89%. Yellow solid with m.p. > 260 °C. ^{1}H NMR (600 MHz, DMSO-d_6) δ 7.21–7.72 (m, 15H), 6.93 (br s, 2H), 6.25 (br s, 1H), 5.44 (br s, 2H). ^{13}C{^{1}H} NMR (151 MHz, DMSO-d_6) δ 160.7, 156.6, 150.8, 140.1, 138.7, 137.8, 136.0, 133.9, 131.3 (2C), 130.9, 130.1, 129.7 (2C), 129.3, 129.2 (2C), 129.1 (6C), 128.9 (2C), 127.8, 126.8 (2C), 126.7, 124.5, 118.0, 115.9, 113.5, 112.0, 98.7, 47.4. Anal. Calcd for $C_{36}H_{25}NO_2$: C, 85.86; H, 5.00; N, 2.78. Found: C, 85.90; H, 4.85; N, 2.92.
**10**	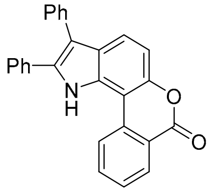	Yield 162 mg, 42%. Beige solid with m.p.= 250 °C. ^{1}H NMR (400 MHz, DMSO-d_6) δ 11.62 (s, 1H), 8.90 (m, 1H), 8.37 (m 1H), 8.05 (m, 1H), 7.73 (m, 1H), 7.67 (d, *J* = 8.7 Hz, 1H), 7.53–7.54 (m, 2H), 7.30–7.43 (m, 8H), 7.22 (d, J = 8.7 Hz, 1H). ^{13}C{^{1}H} NMR (101 MHz, DMSO-d_6) δ 160.5, 148.2, 136.6, 135.2, 134.1, 133.5, 131.9, 130.7, 129.9 (2C), 129.8, 129.5 (2C), 128.7 (2C), 128.4, 128.2 (2C), 127.8, 126.5 (2C), 125.8, 121.4, 120.2, 115.0, 110.7, 104.1. Anal. Calcd for $C_{27}H_{17}NO_2$: C, 83.70; H, 4.42; N, 3.62. Found: C, 83.82; H, 4.35; N, 3.52.

Cmpd	Structure	Characterization
**12**	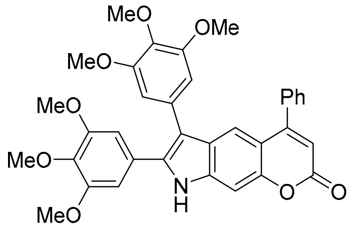	Yield 1211 mg, 68%. Beige solid with m.p.= 210–212 °C. ^{1}H NMR (400 MHz, DMSO-d_6) δ 11.96 (s, 1H), 7.61–7.63 (m, 3H), 7.53–7.54 (m, 3H), 7.48 (s, 1H), 6.84 (s, 2H), 6.62 (s, 2H), 6.23 (s, 1H), 3.68 (s, 3H), 3.66 (s, 6H), 3.64 (s, 3H), 3.60 (s, 6H). ^{13}C{^{1}H} NMR (101 MHz, DMSO-d_6) δ 160.4, 156.4, 153.0 (2C), 152.7 (2C), 150.3, 137.6, 137.5, 136.3, 136.1, 135.8, 129.5, 128.7 (2C), 128.5 (2C), 126.6, 125.6, 117.3, 113.6, 112.6, 111.2, 107.0 (2C), 105.8 (2C), 97.9, 60.14 (3C), 60.07 (3C), 55.8 (6C), 55.7 (6C). Anal. Calcd for $C_{35}H_{31}NO_8$: C, 70.82; H, 5.26; N, 2.36. Found: C, 70.72; H, 5.16; N, 2.51.
**13**	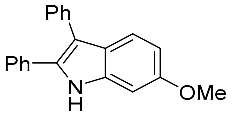	^{1}H NMR (400 MHz, DMSO) δ 11.37 (s, 1H), 7.24–7.44 (m, 11H), 6.94 (d, J = 2.3 Hz, 1H), 6.71 (dd, J = 8.7 Hz, J = 2.3 Hz, 1H), 3.80 (s, 3H). ^{13}C{^{1}H} NMR (101 MHz, DMSO) δ 156.1, 136.9, 135.4, 132.7 (2C), 129.6 (2C), 128.6 (2C), 128.4 (2C), 127.8 (2C), 127.1, 126.0, 122.4, 119.3, 113.3, 109.9, 94.4, 55.2.

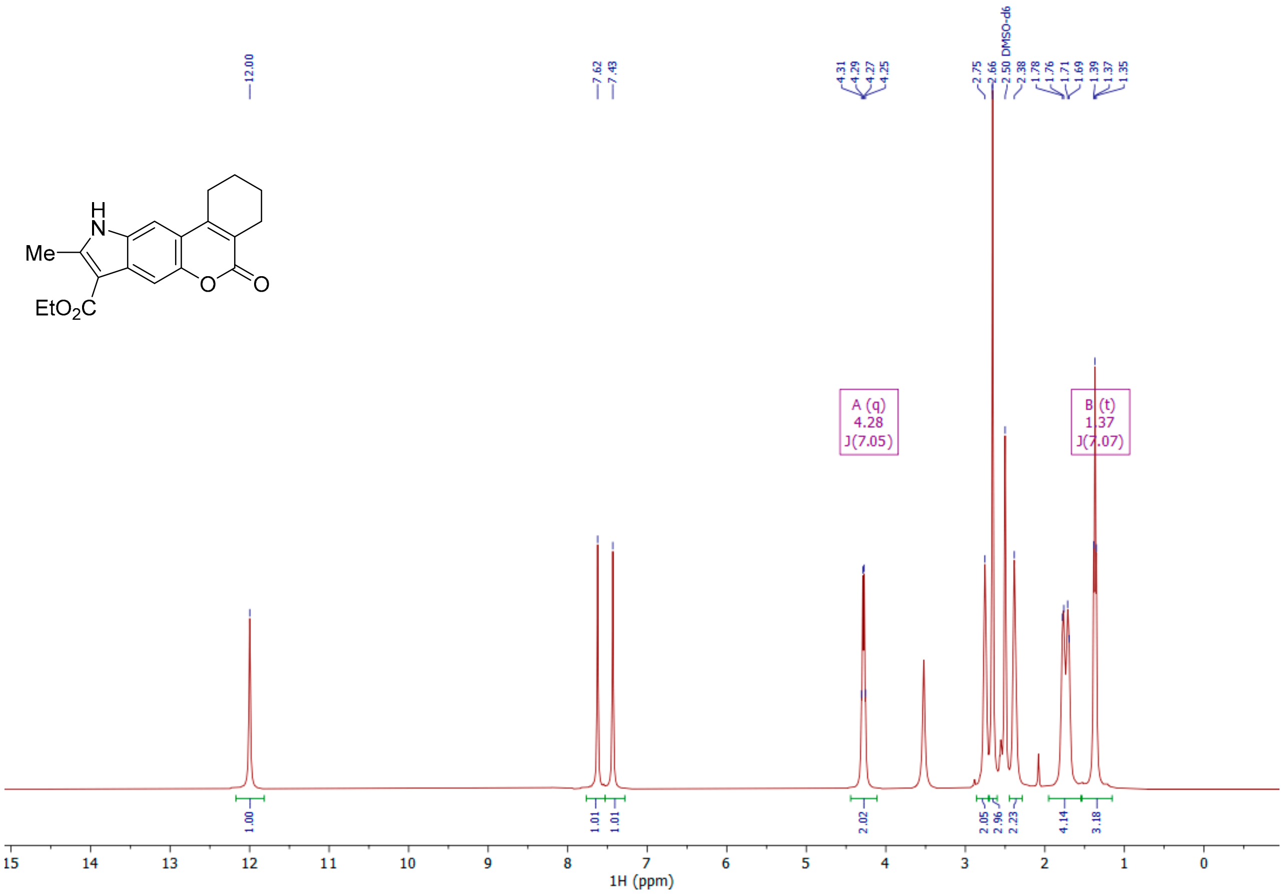

Figure S1. ^{1}H NMR spectrum of **2**.

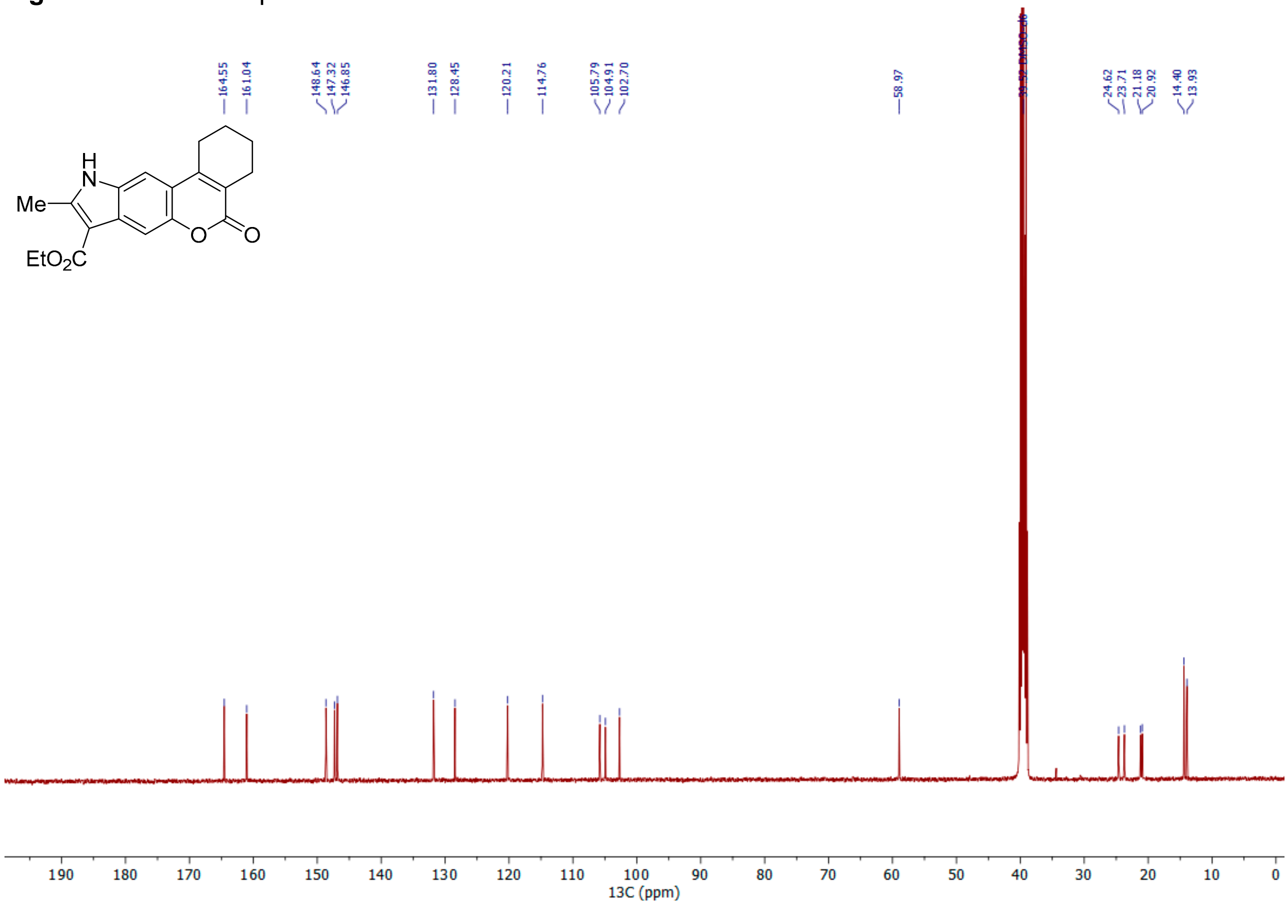

Figure S2. ^{13}C NMR spectrum of **2**.

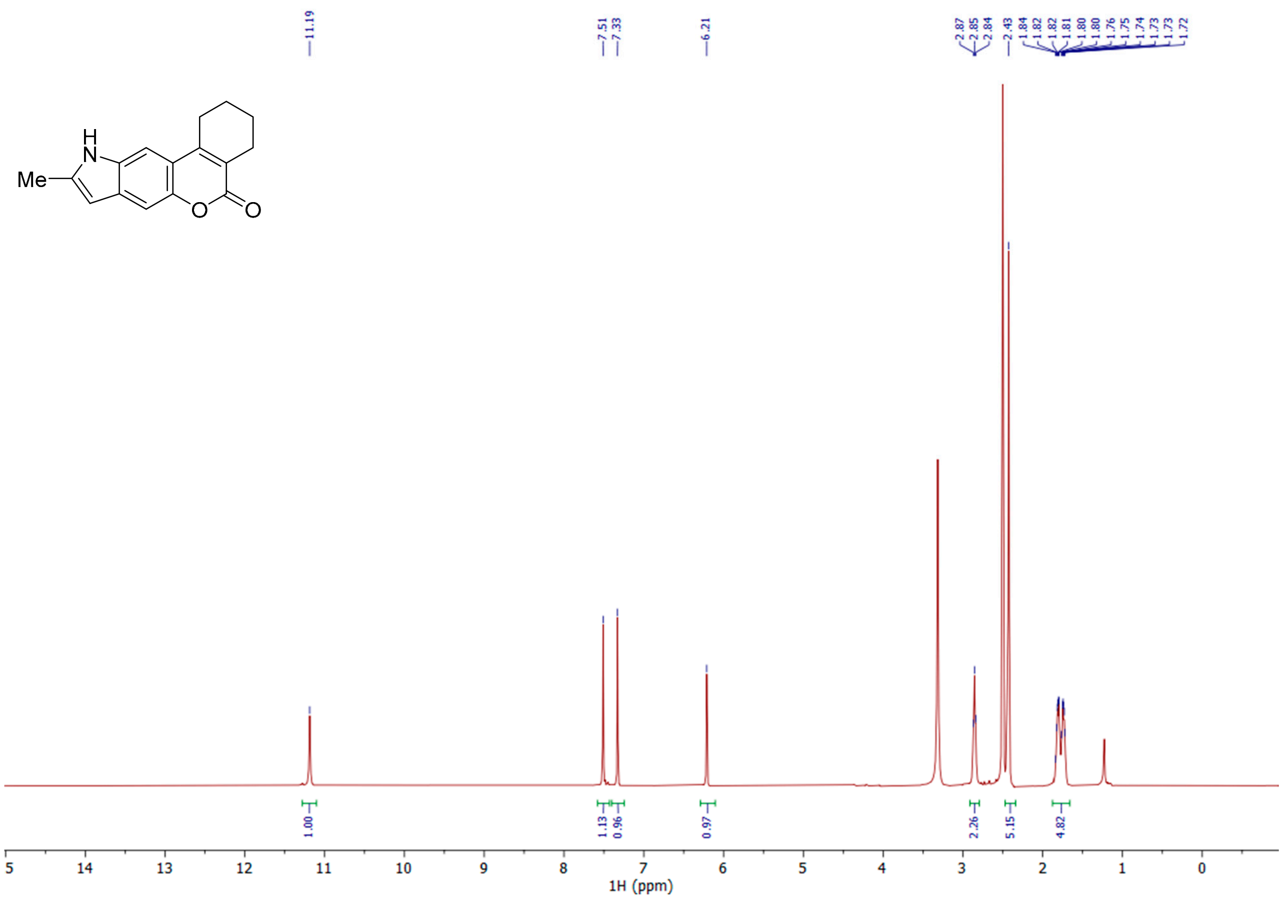

Figure S3. ^{1}H NMR spectrum of **3**.

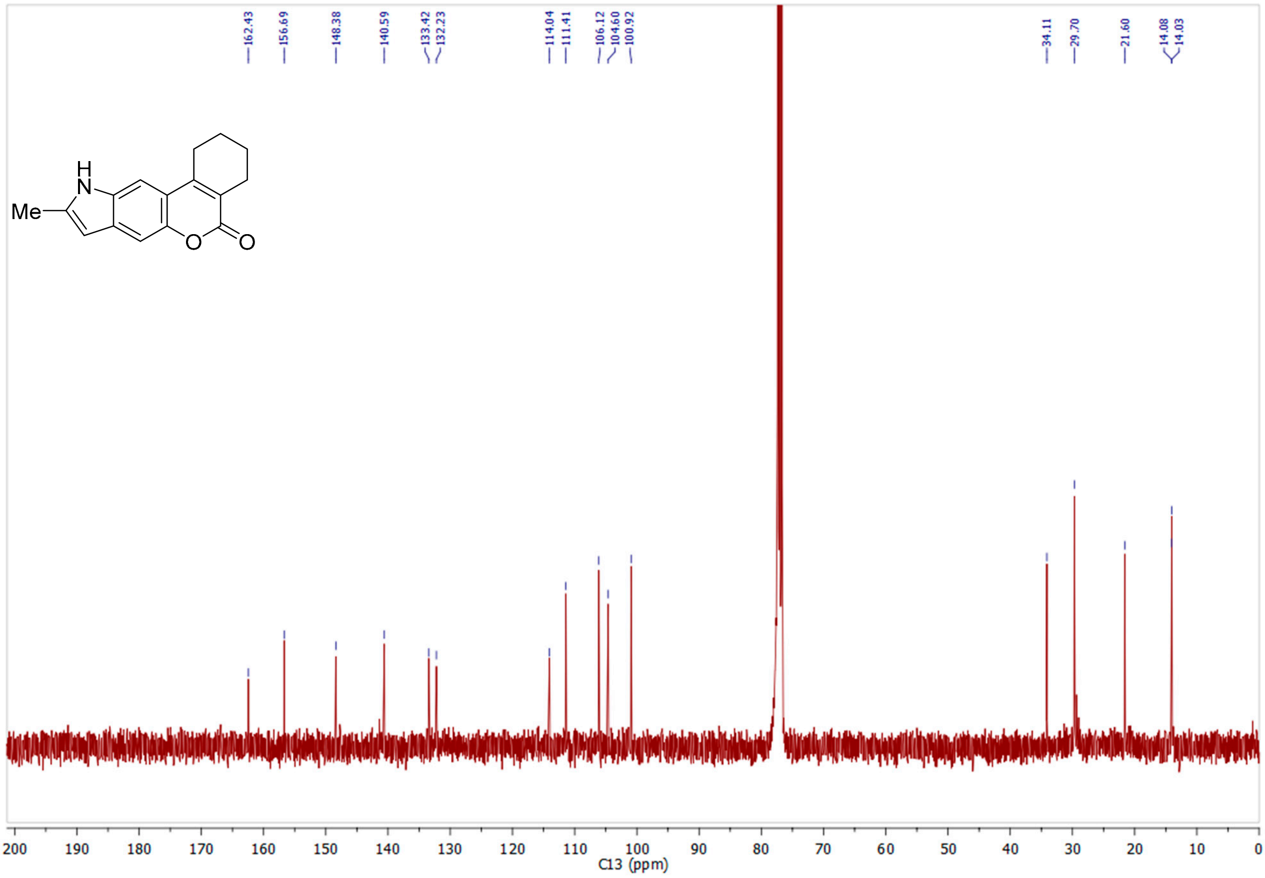

Figure S4. ^{13}C NMR spectrum of **3**.

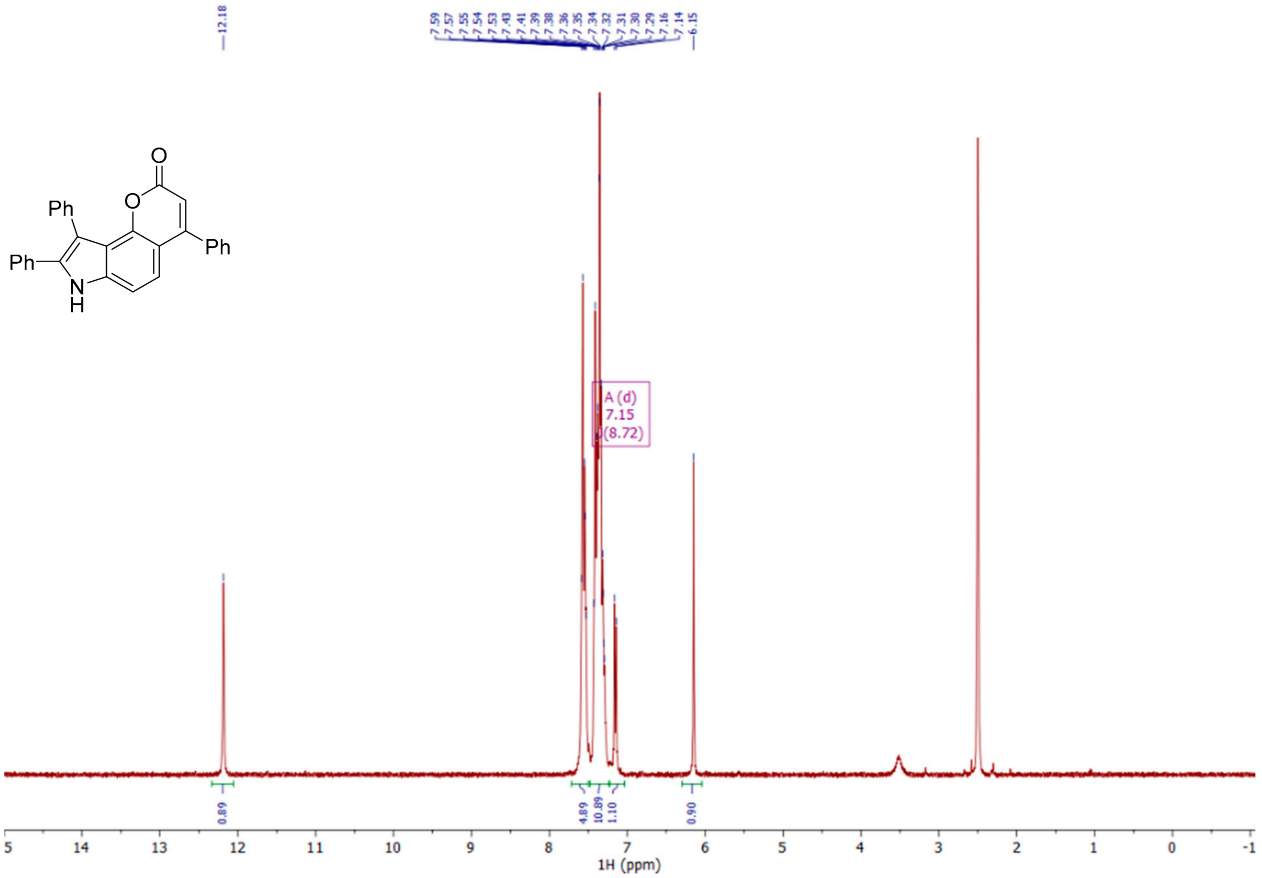

Figure S5. ^{1}H NMR spectrum of **6a**.

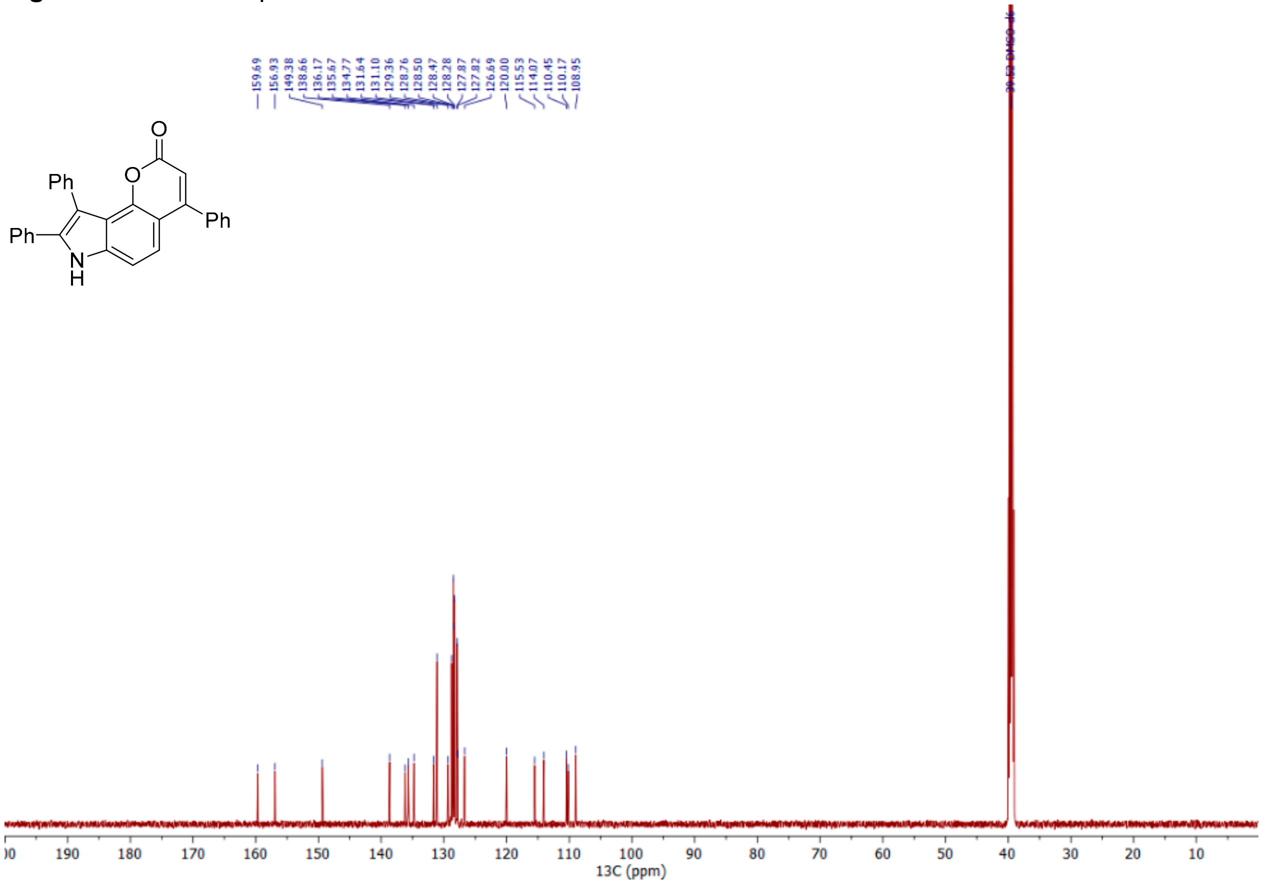

Figure S6. ^{13}C NMR spectrum of **6a**.

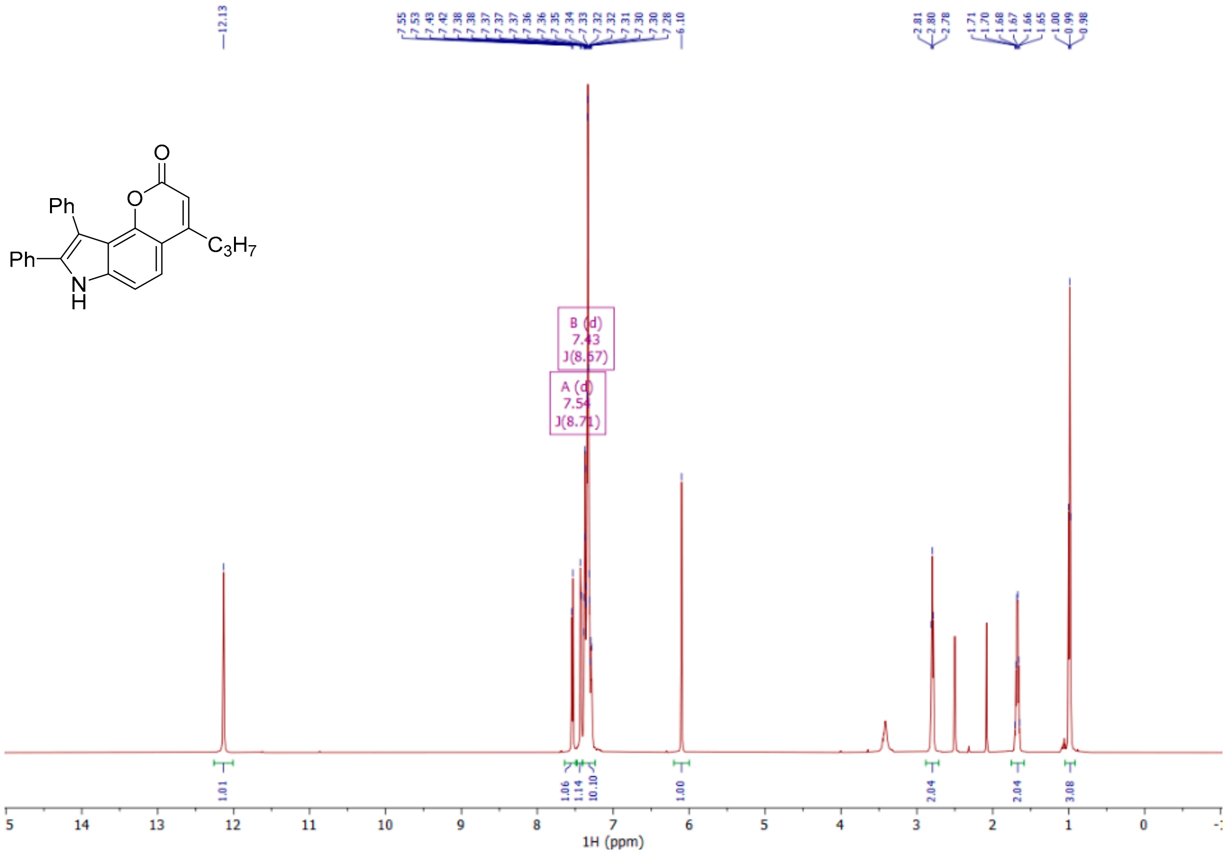

Figure S7. ^{1}H NMR spectrum of **6b**.

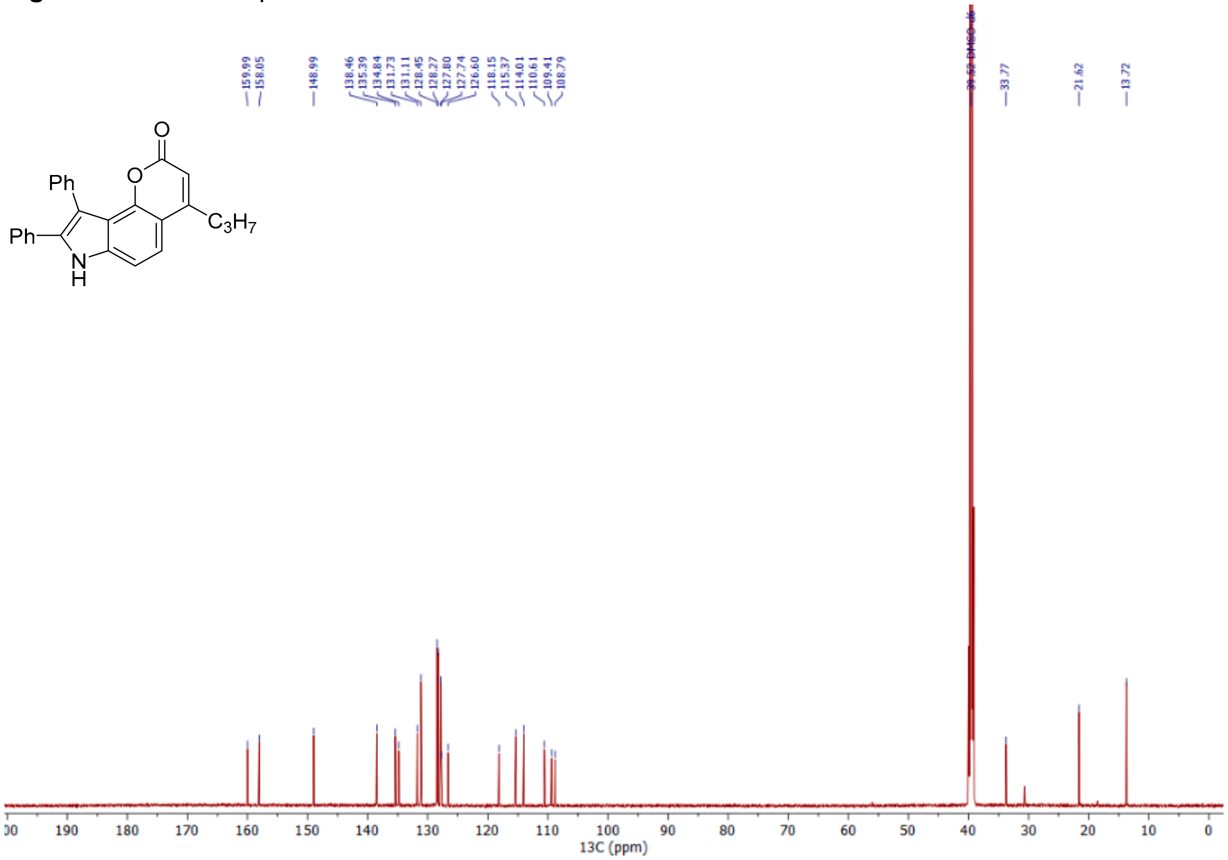

Figure S8. ^{13}C NMR spectrum of **6b**.

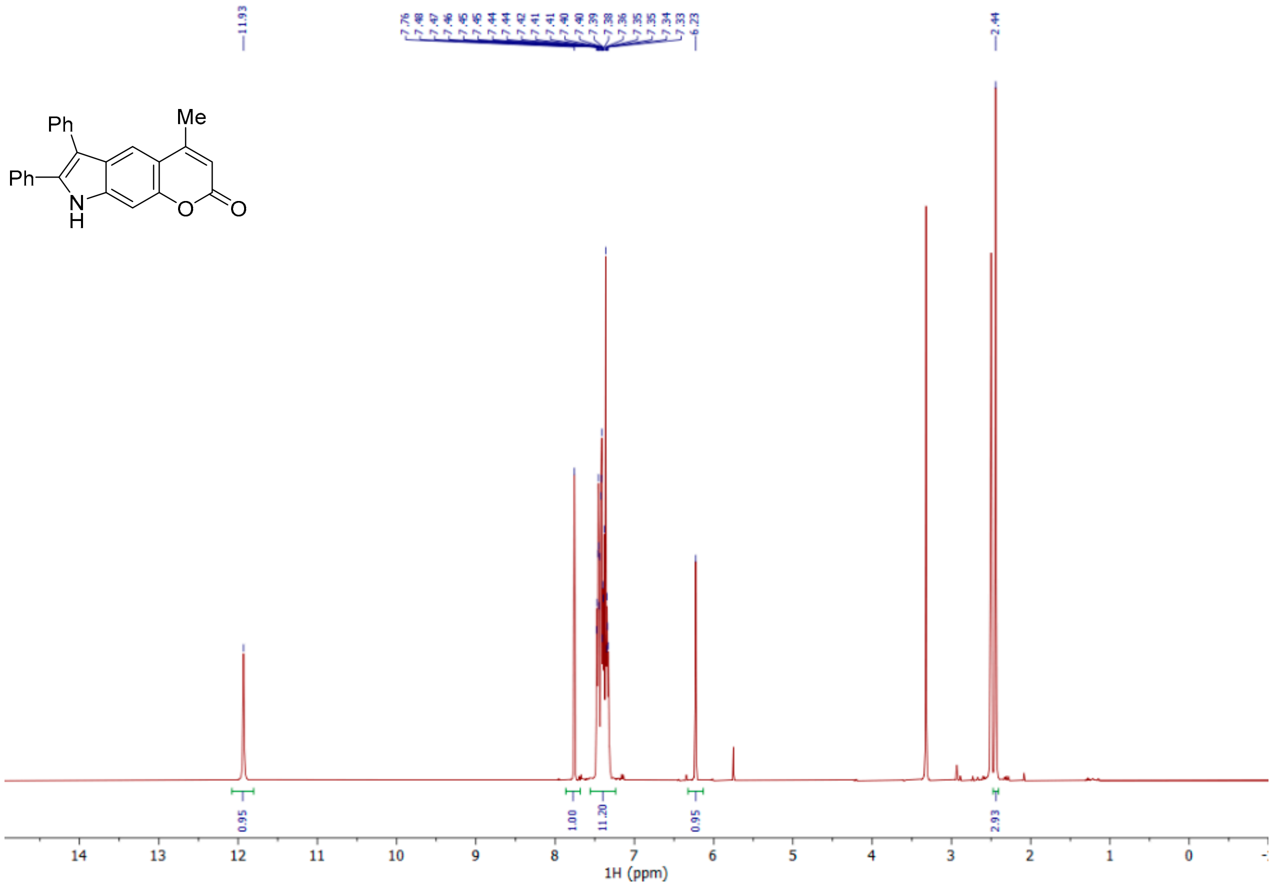

Figure S9. ^{1}H NMR spectrum of **7a**.

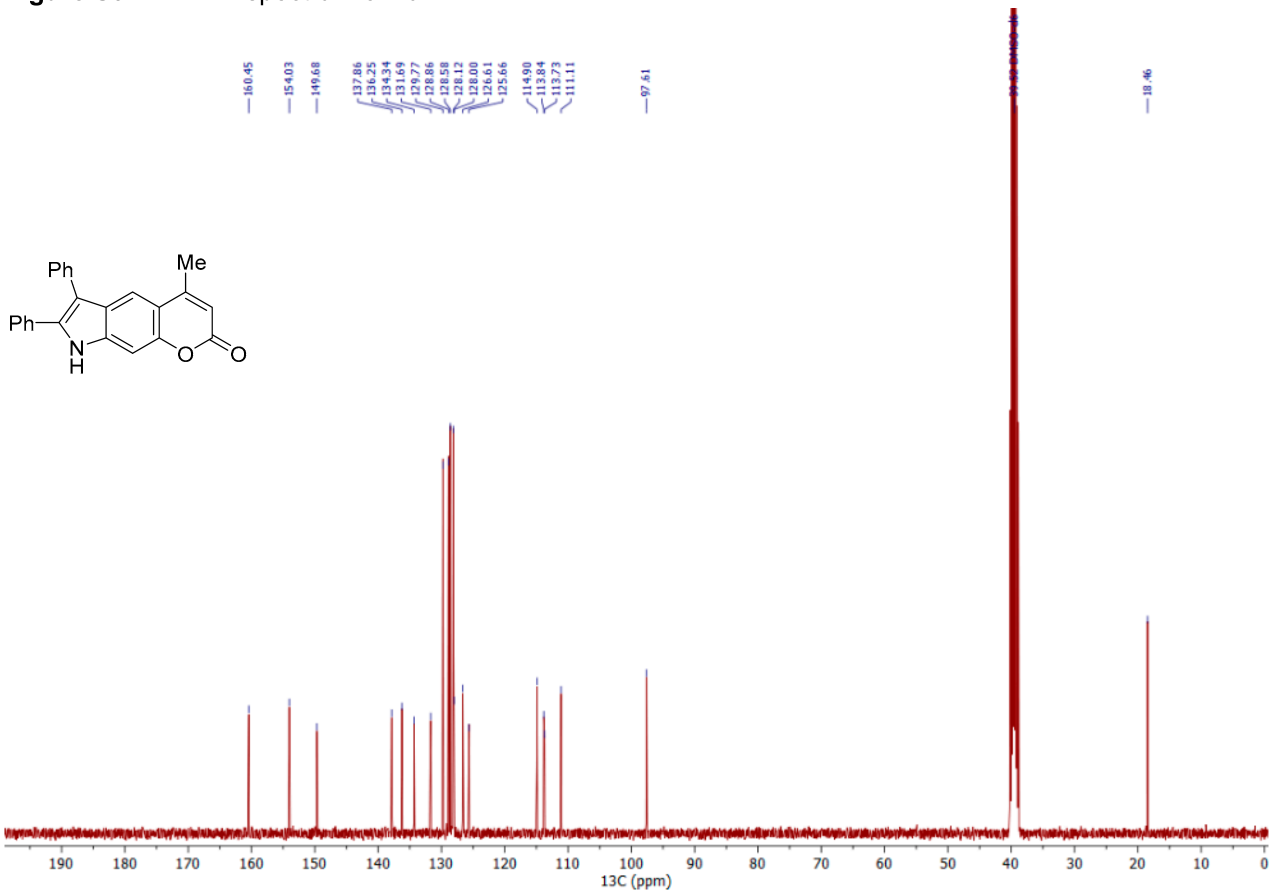

Figure S10. ^{13}C NMR spectrum of **7a.**

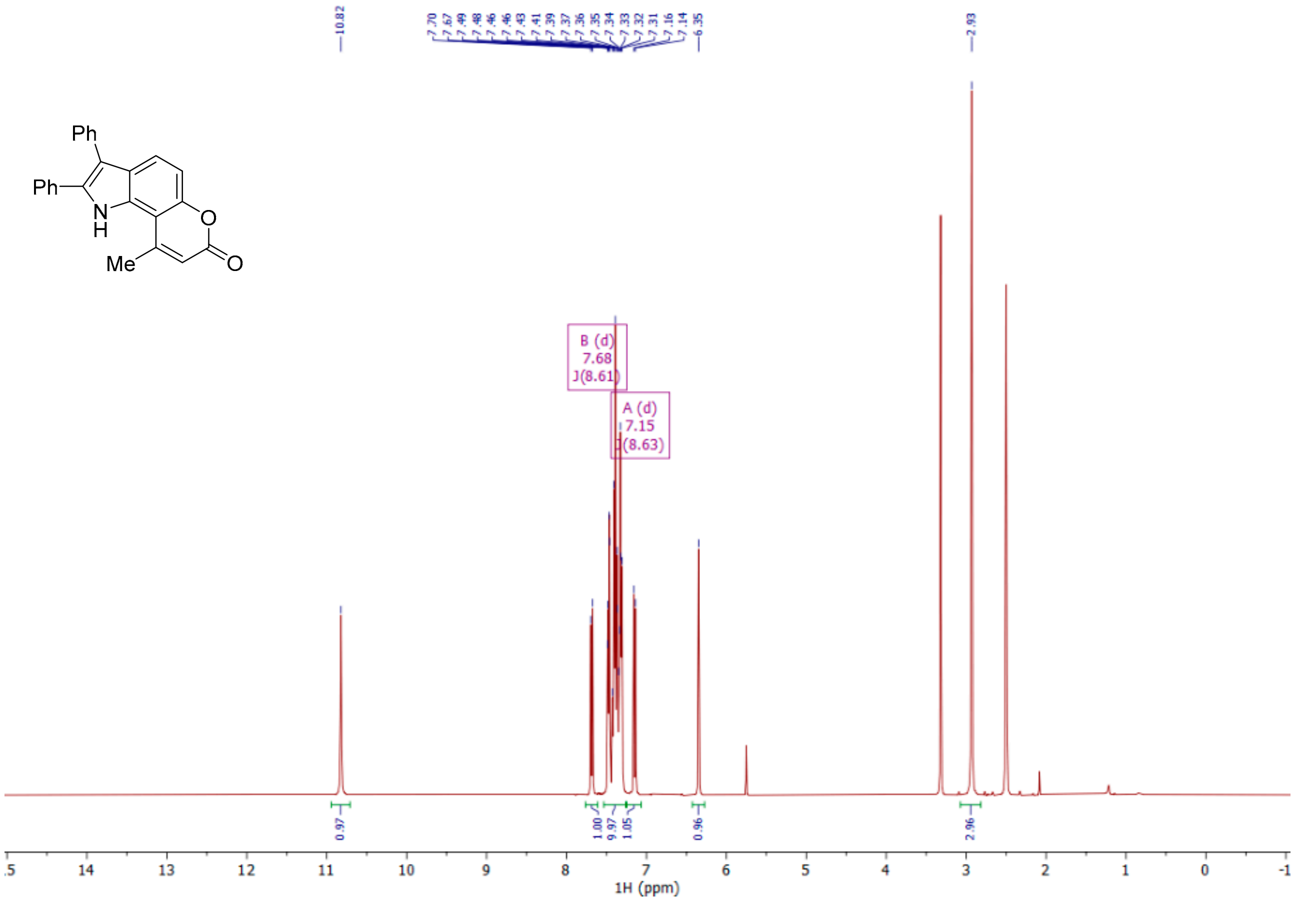

Figure S11. ^{1}H NMR spectrum of **8a**.

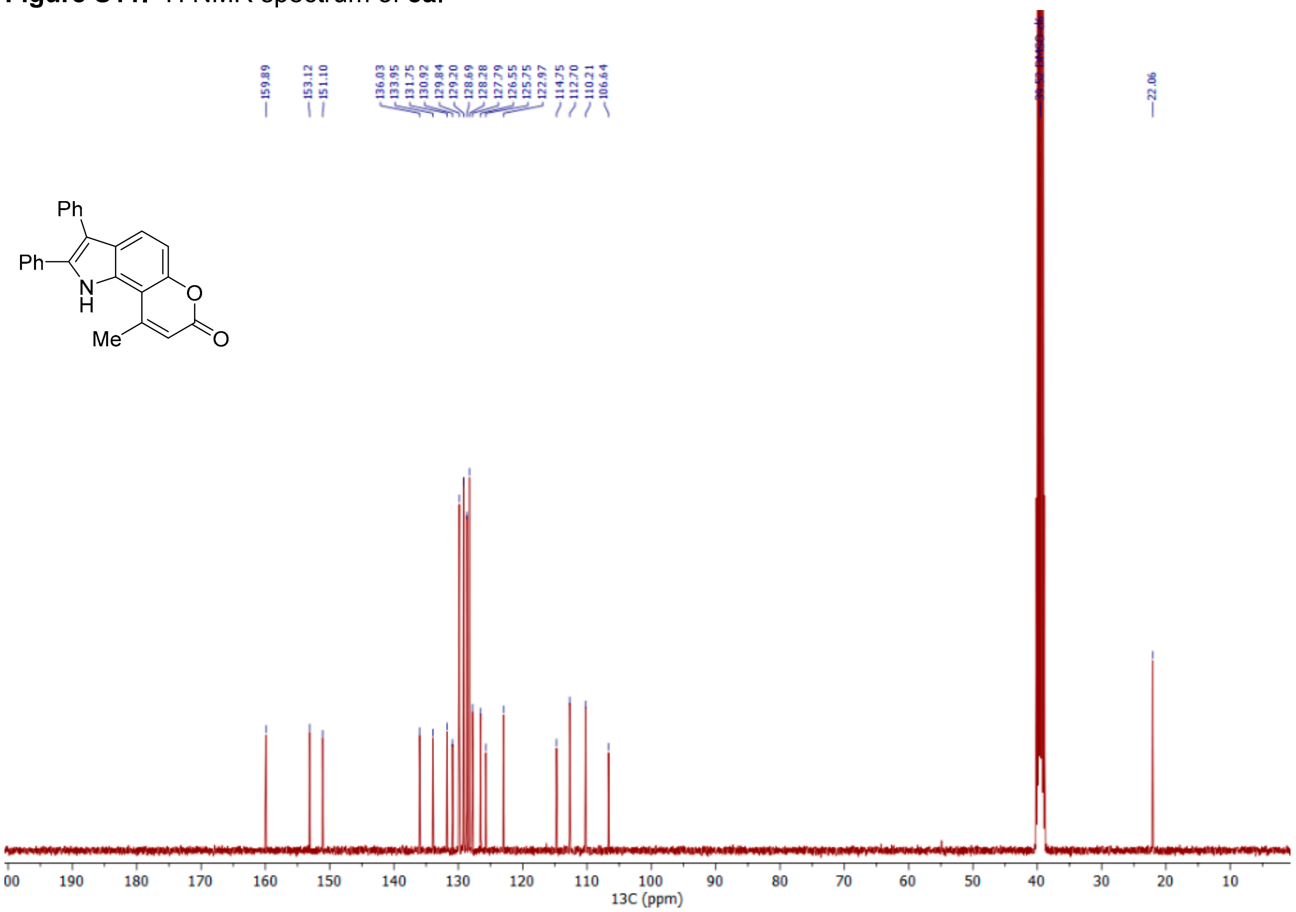

Figure S12. ^{13}C NMR spectrum of **8a**.

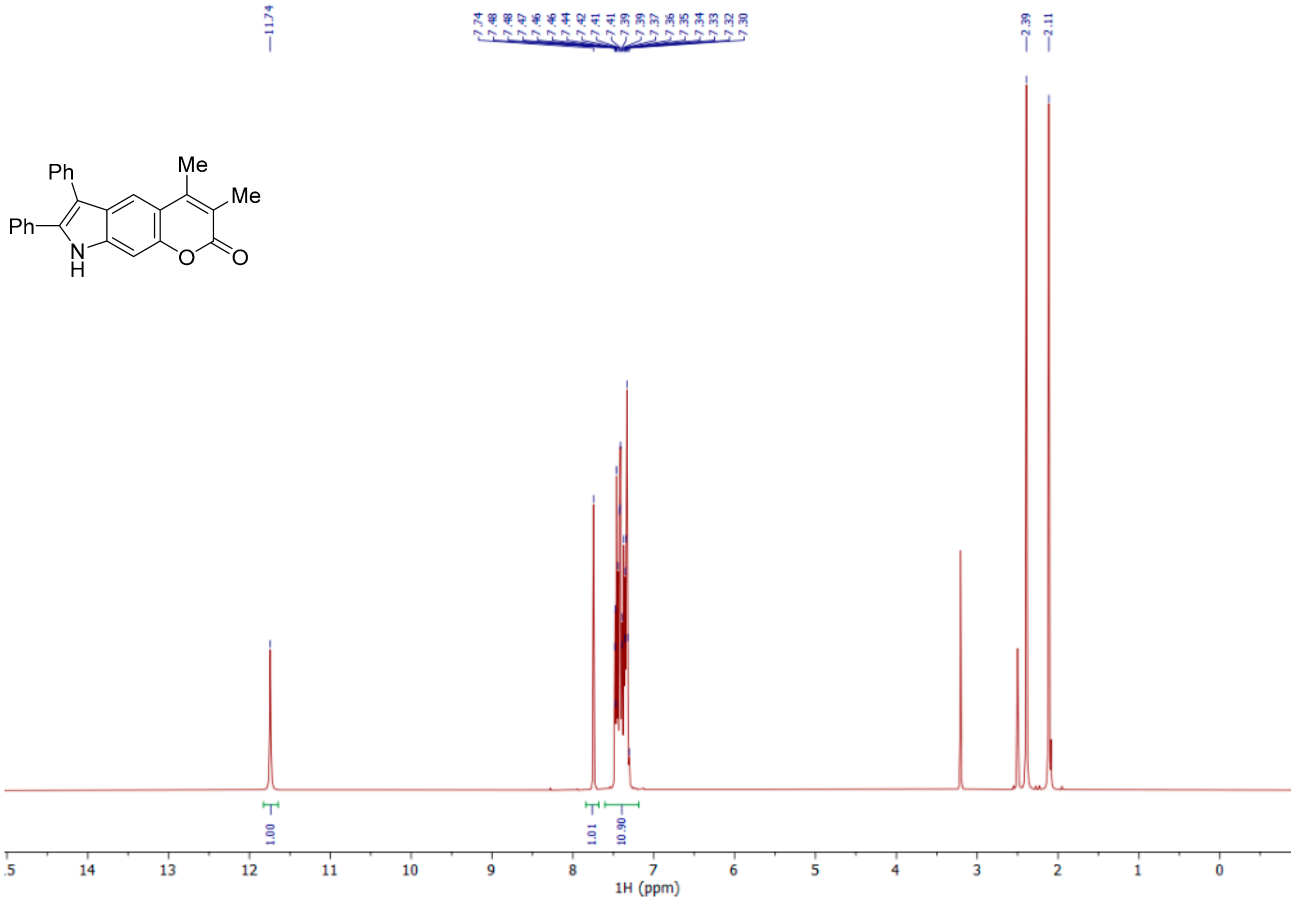

Figure S13. ^{1}H NMR spectrum of **7b**.

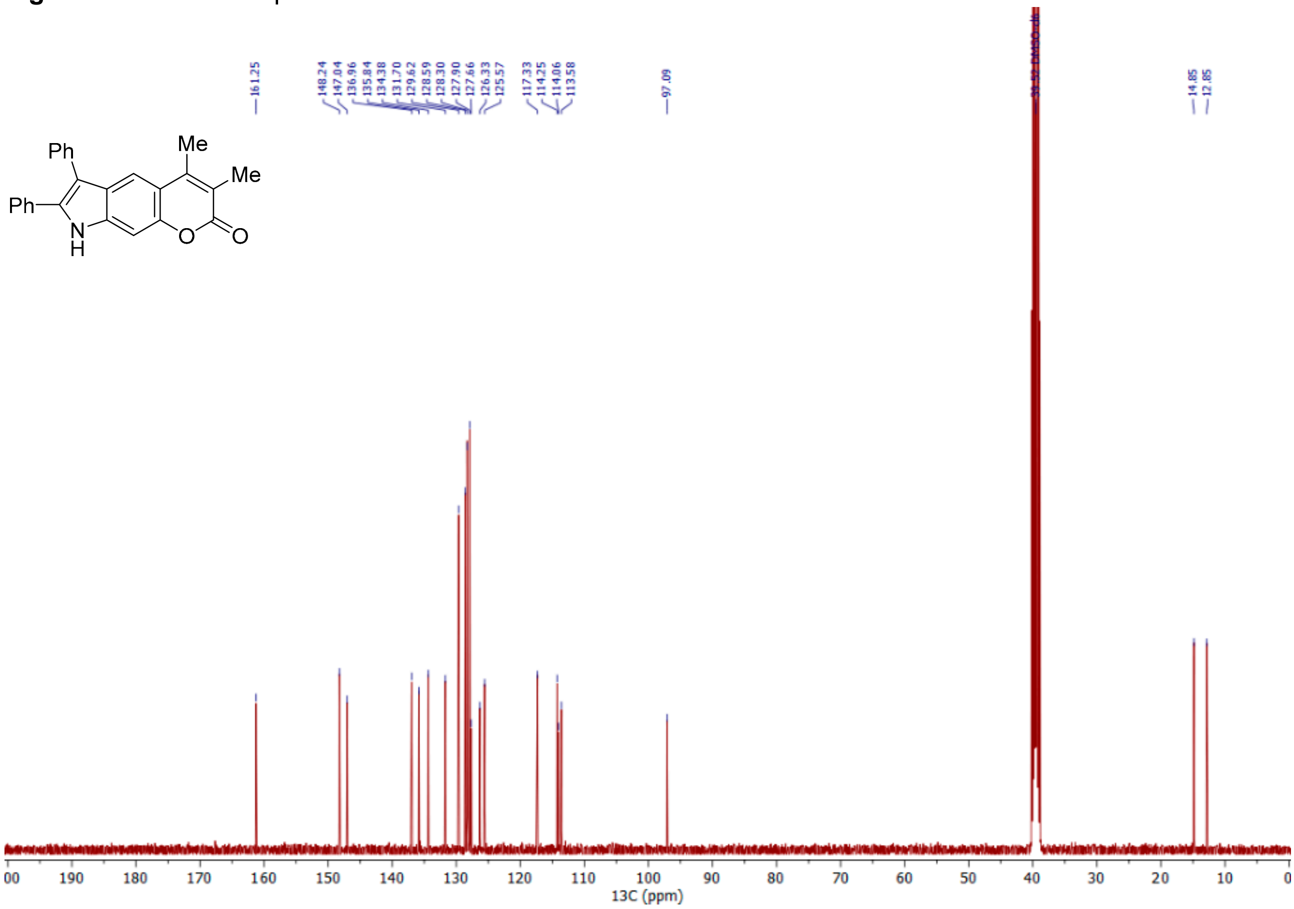

Figure S14. ^{13}C NMR spectrum of **7b**.

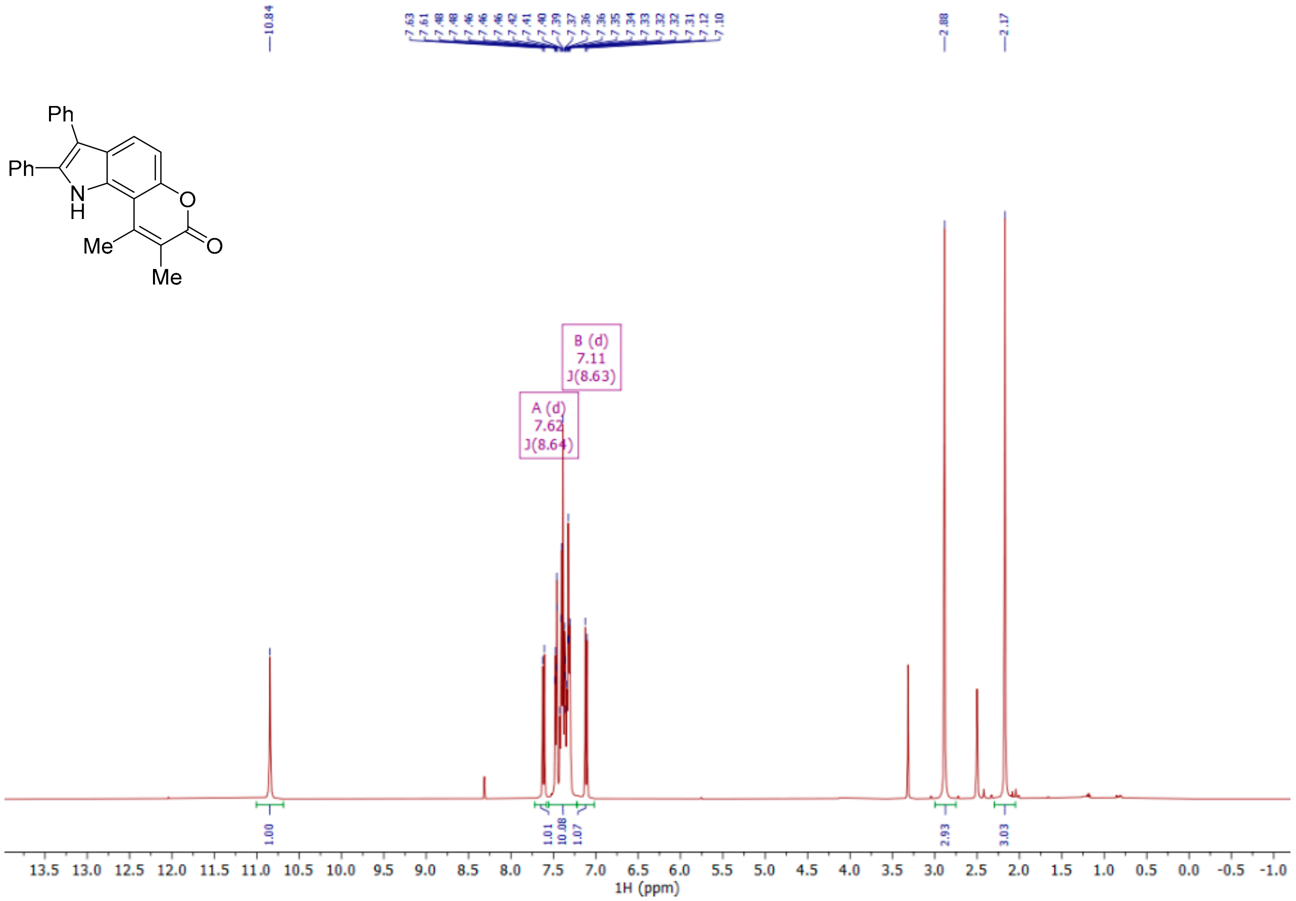

Figure S15. ^{1}H NMR spectrum of **8b**.

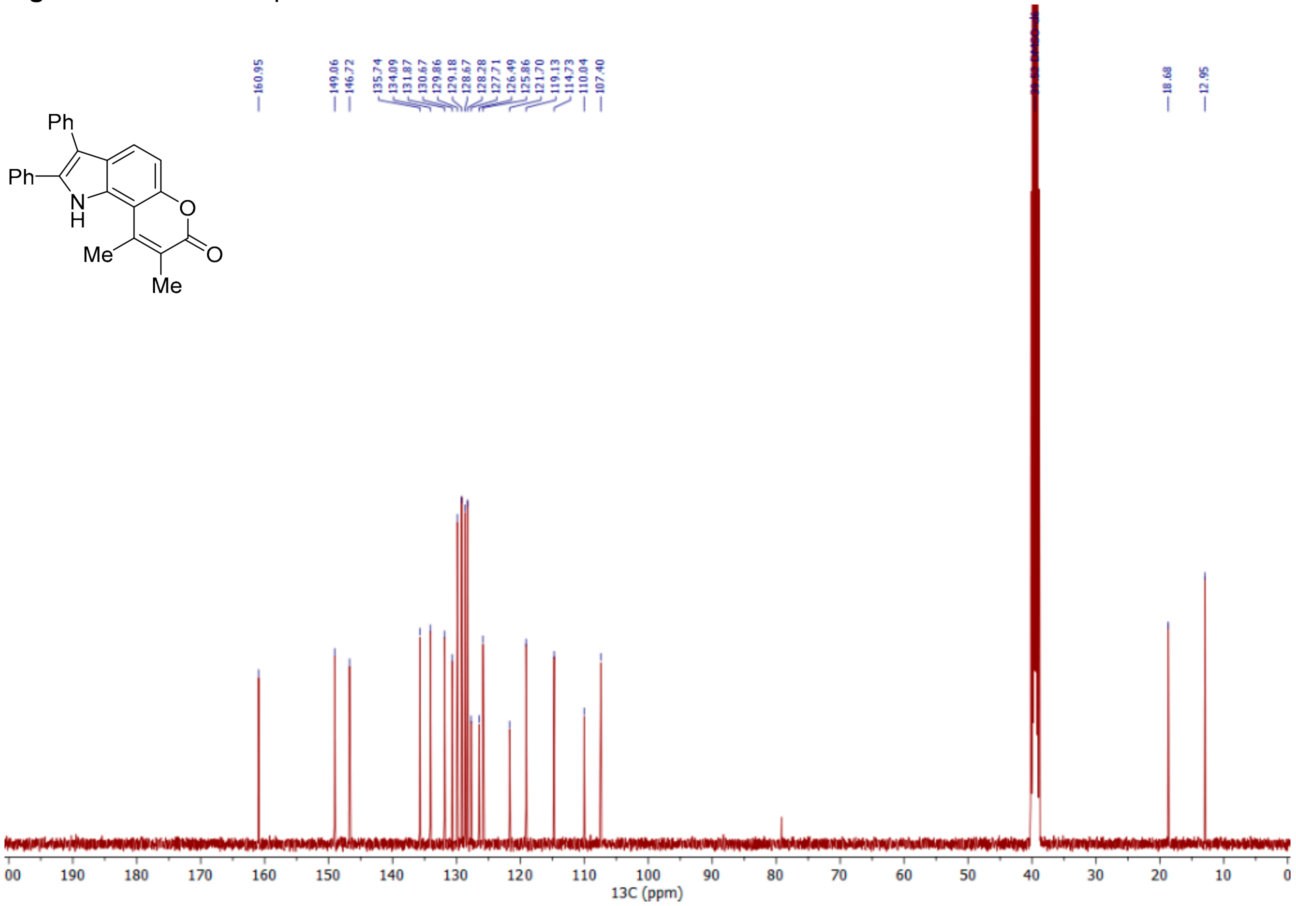

Figure S16. ^{13}C NMR spectrum of **8b**.

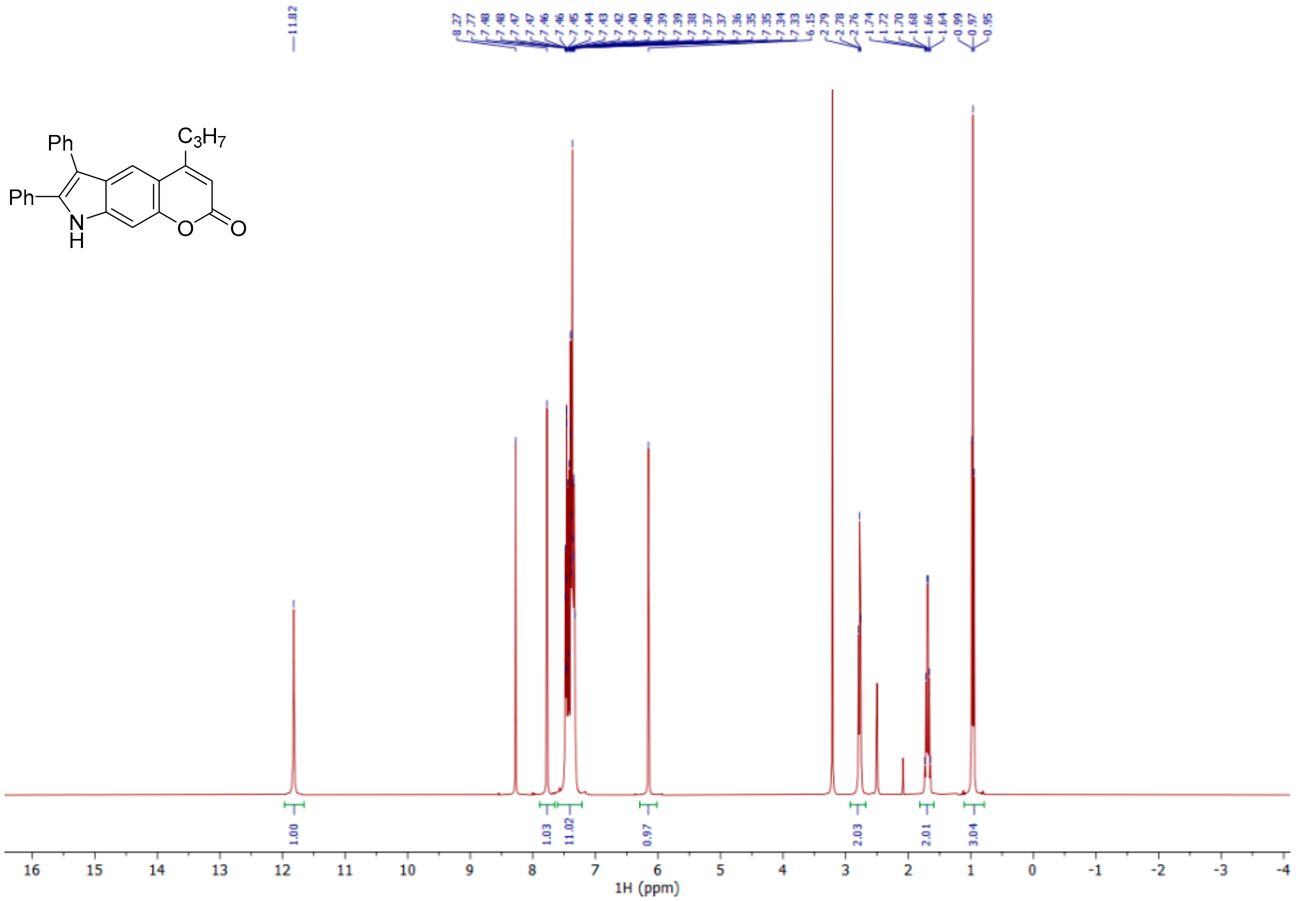

Figure S17. ^{1}H NMR spectrum of **7c**.

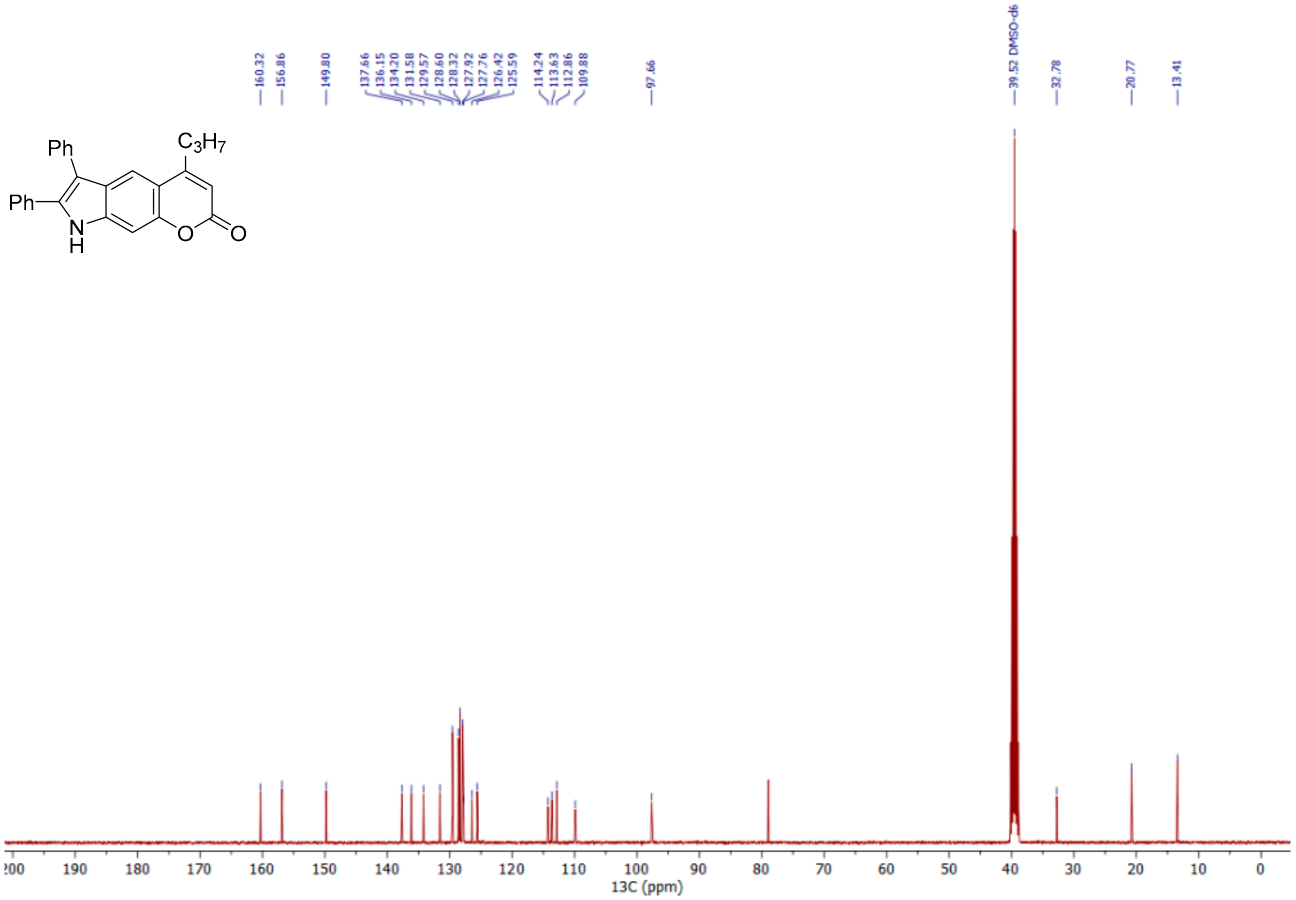

Figure S18. ^{13}C NMR spectrum of **7c**.

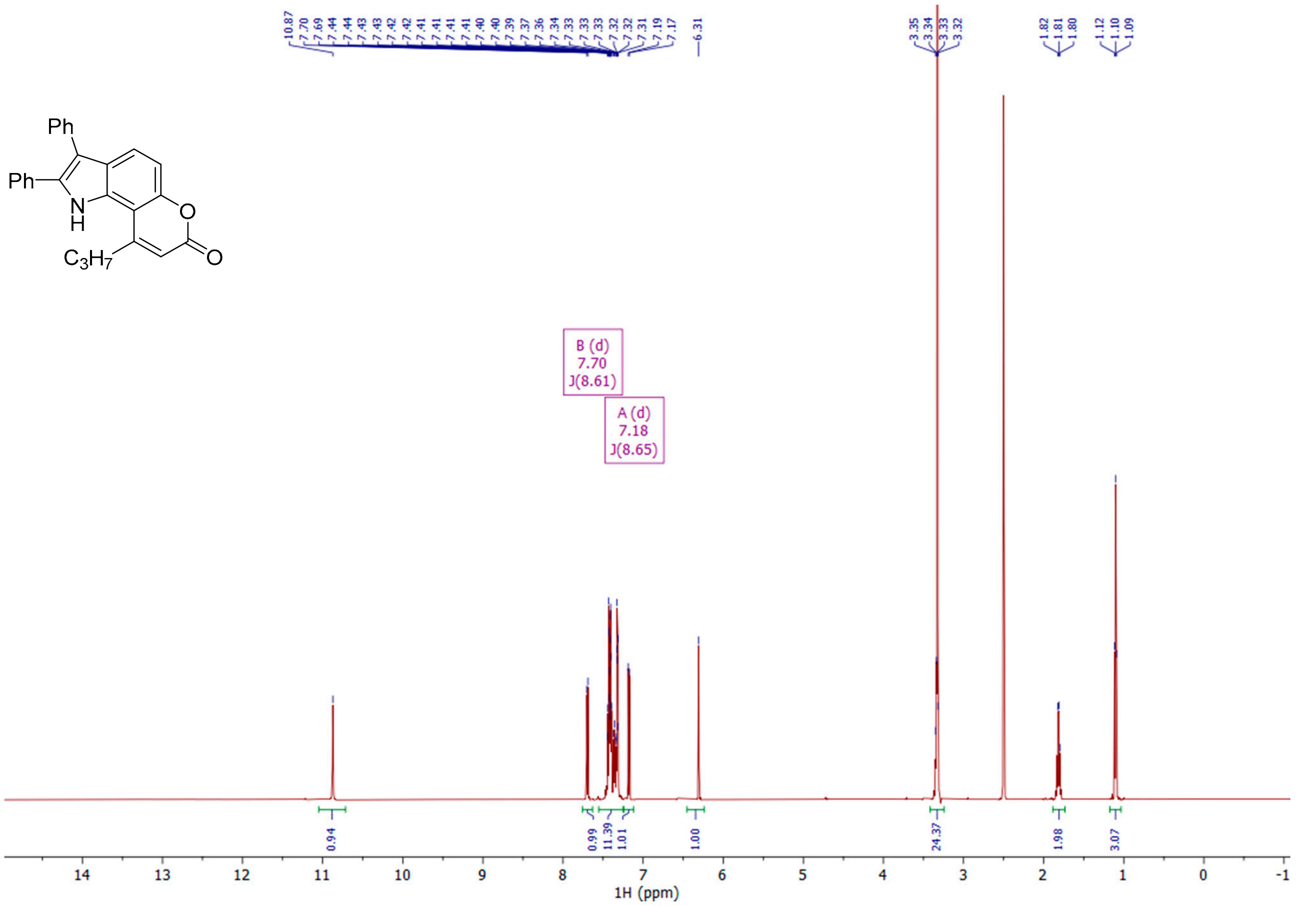

Figure S19. ^{1}H NMR spectrum of **8c**.

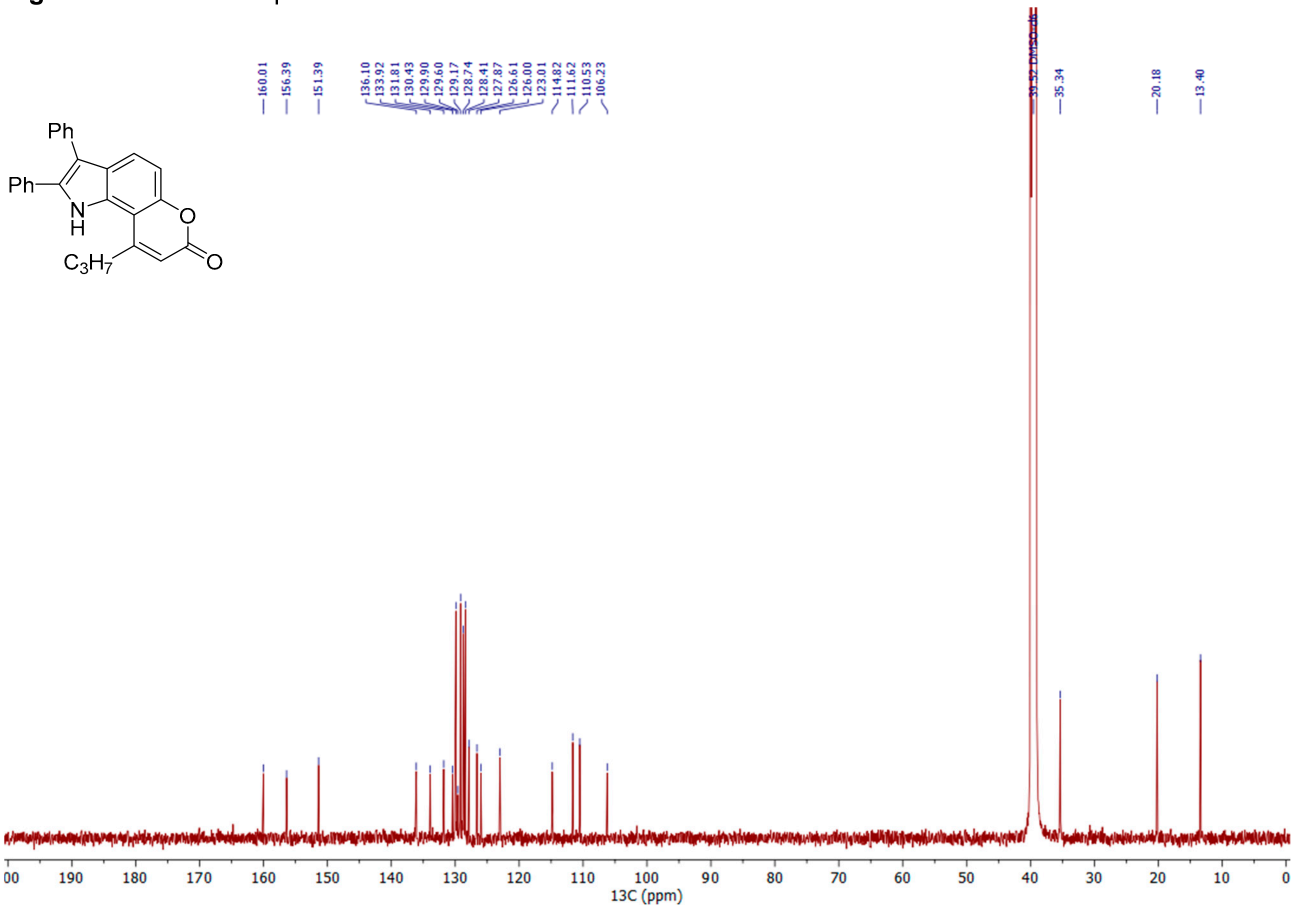

Figure S20. ^{13}C NMR spectrum of **8c**.

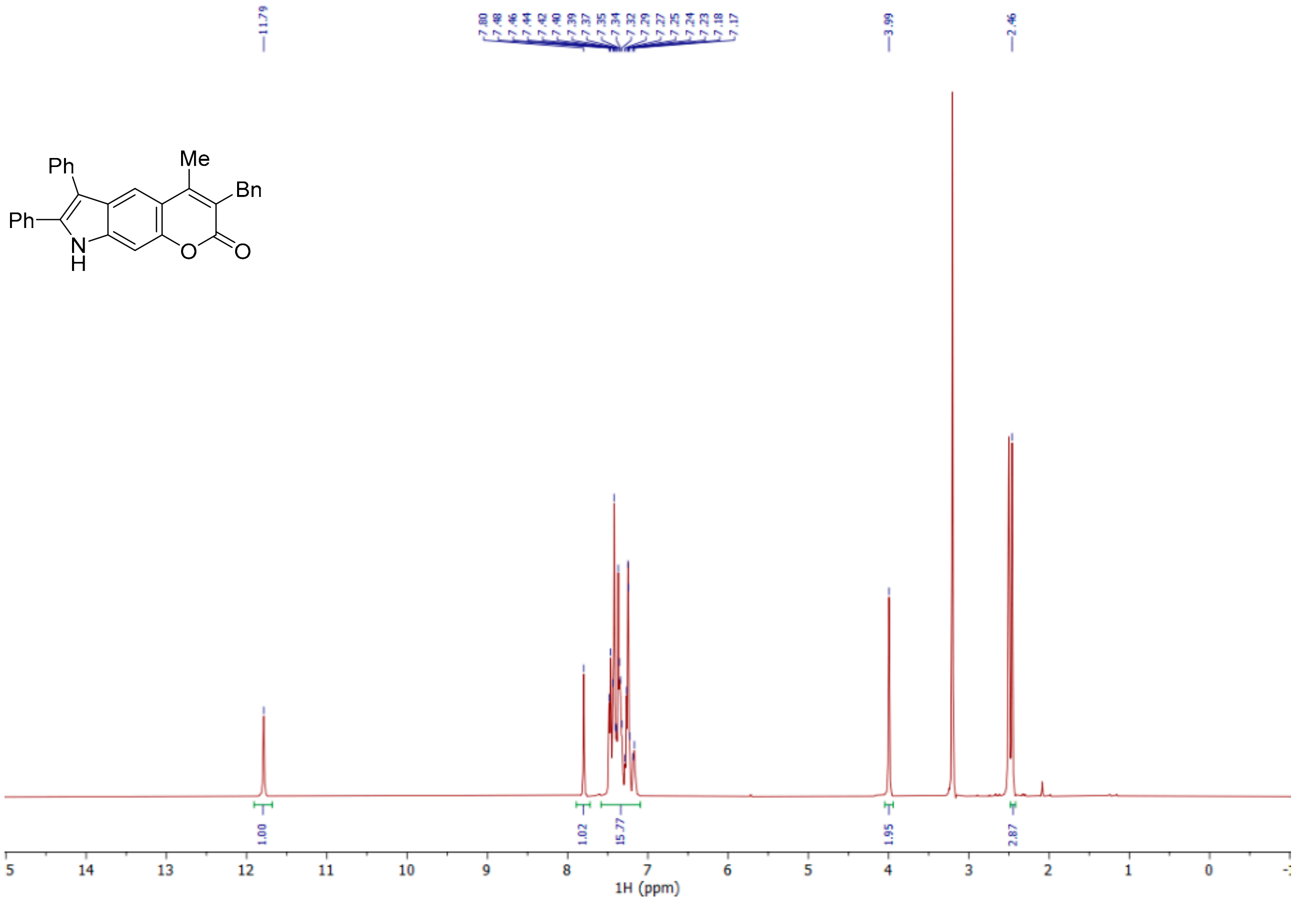

Figure S21. ^{1}H NMR spectrum of **7d**.

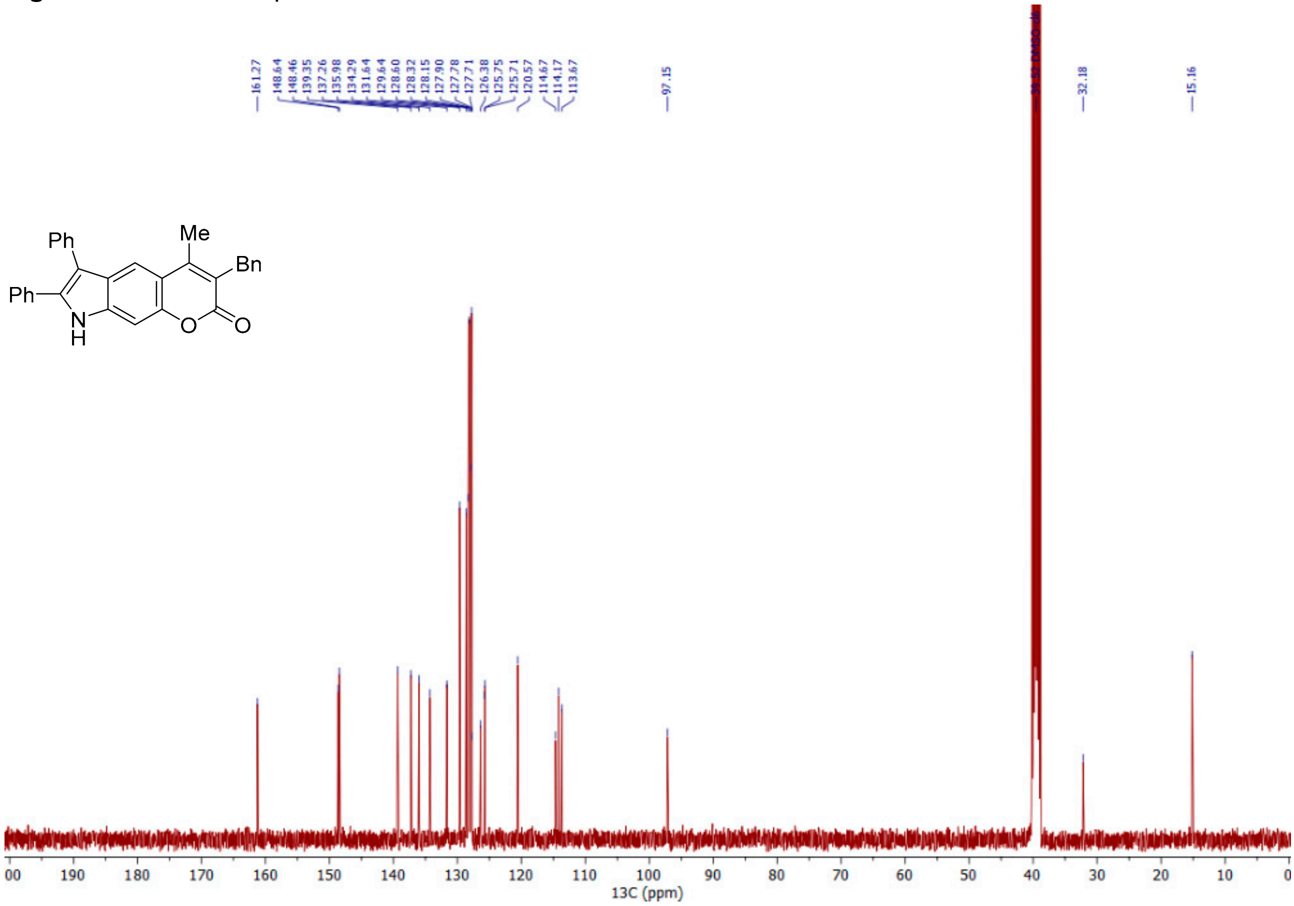

Figure S22. ^{13}C NMR spectrum of **7d**.

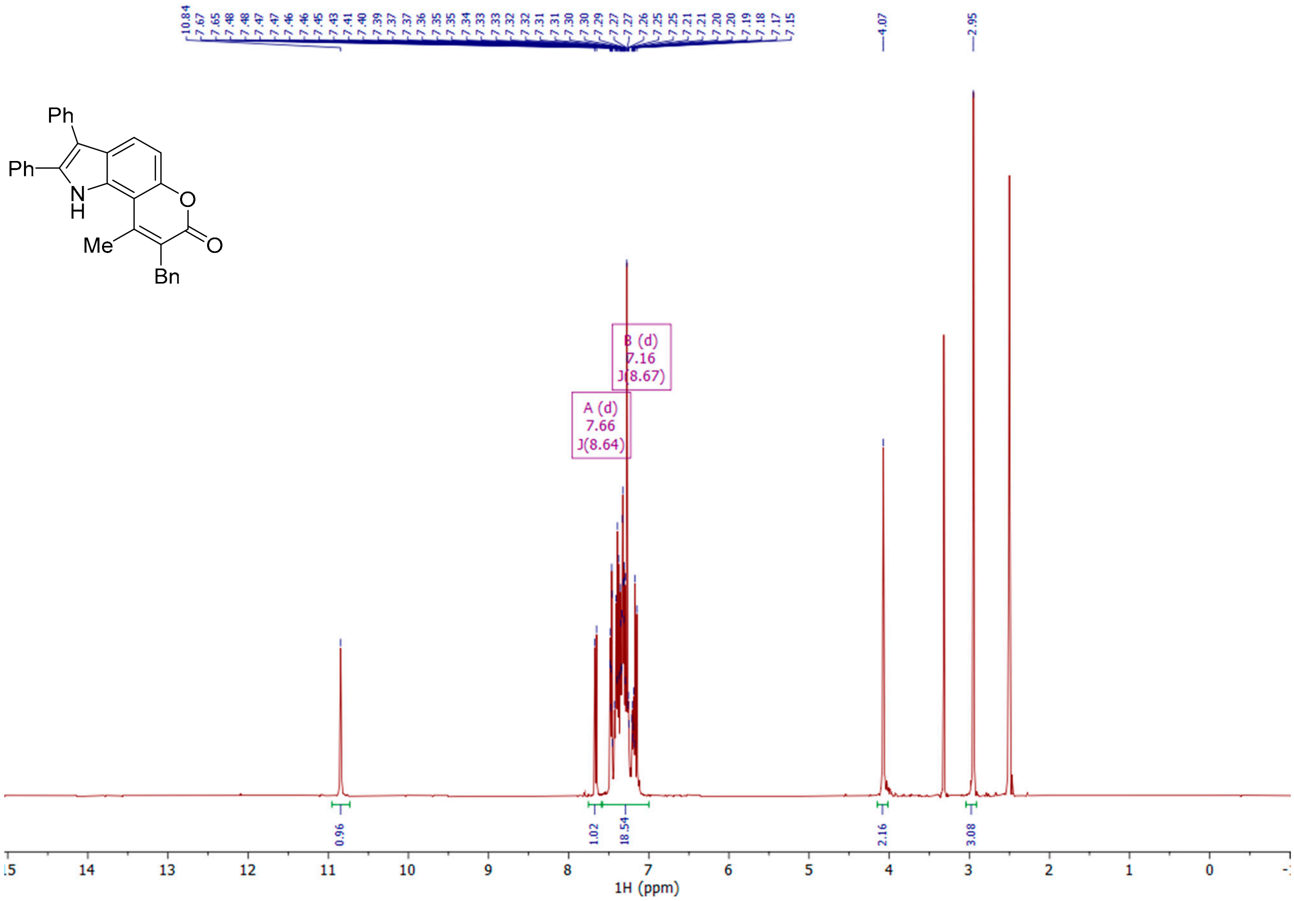

Figure S23. ^{1}H NMR spectrum of **8d**.

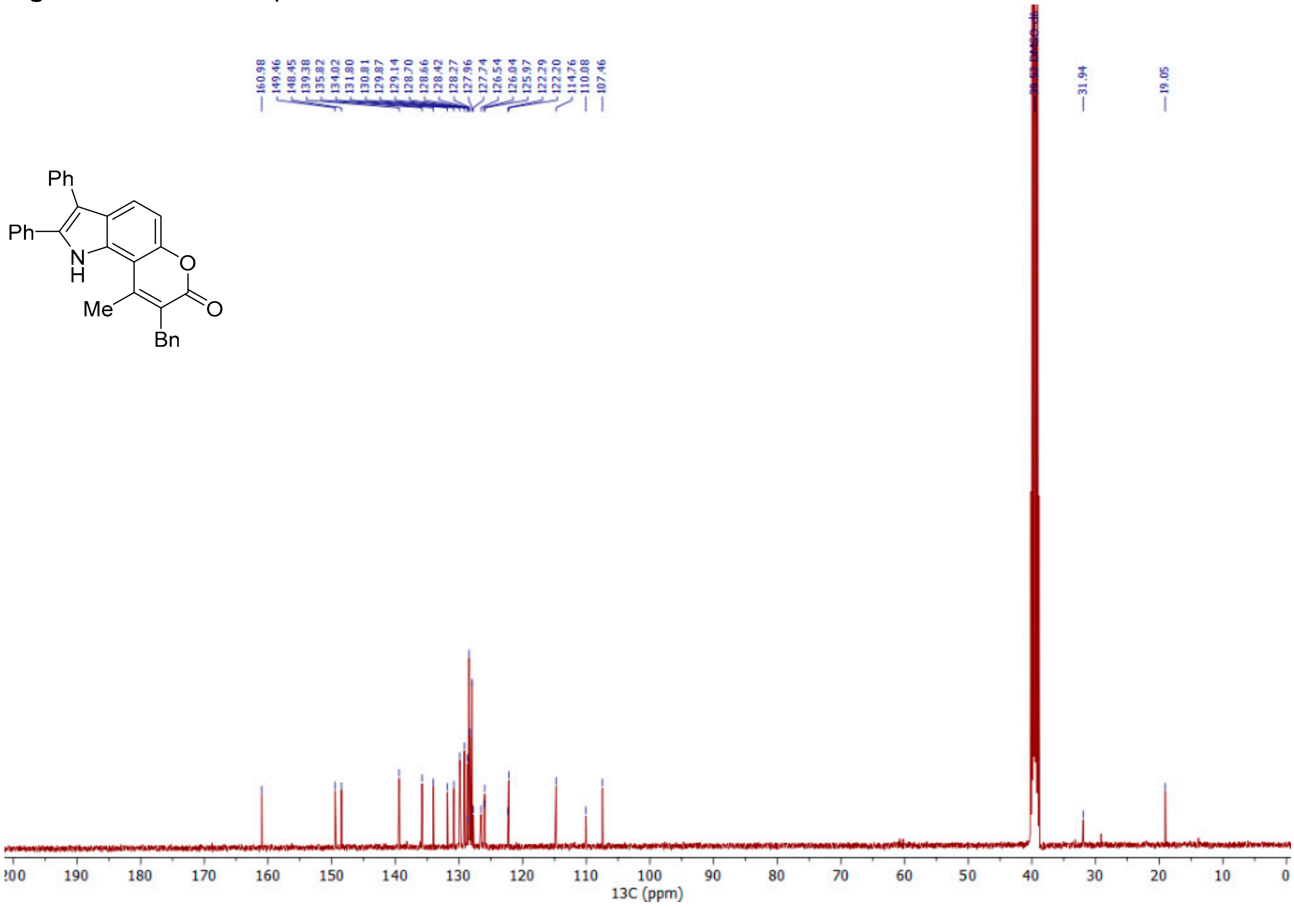

Figure S24. ^{13}C NMR spectrum of **8d**.

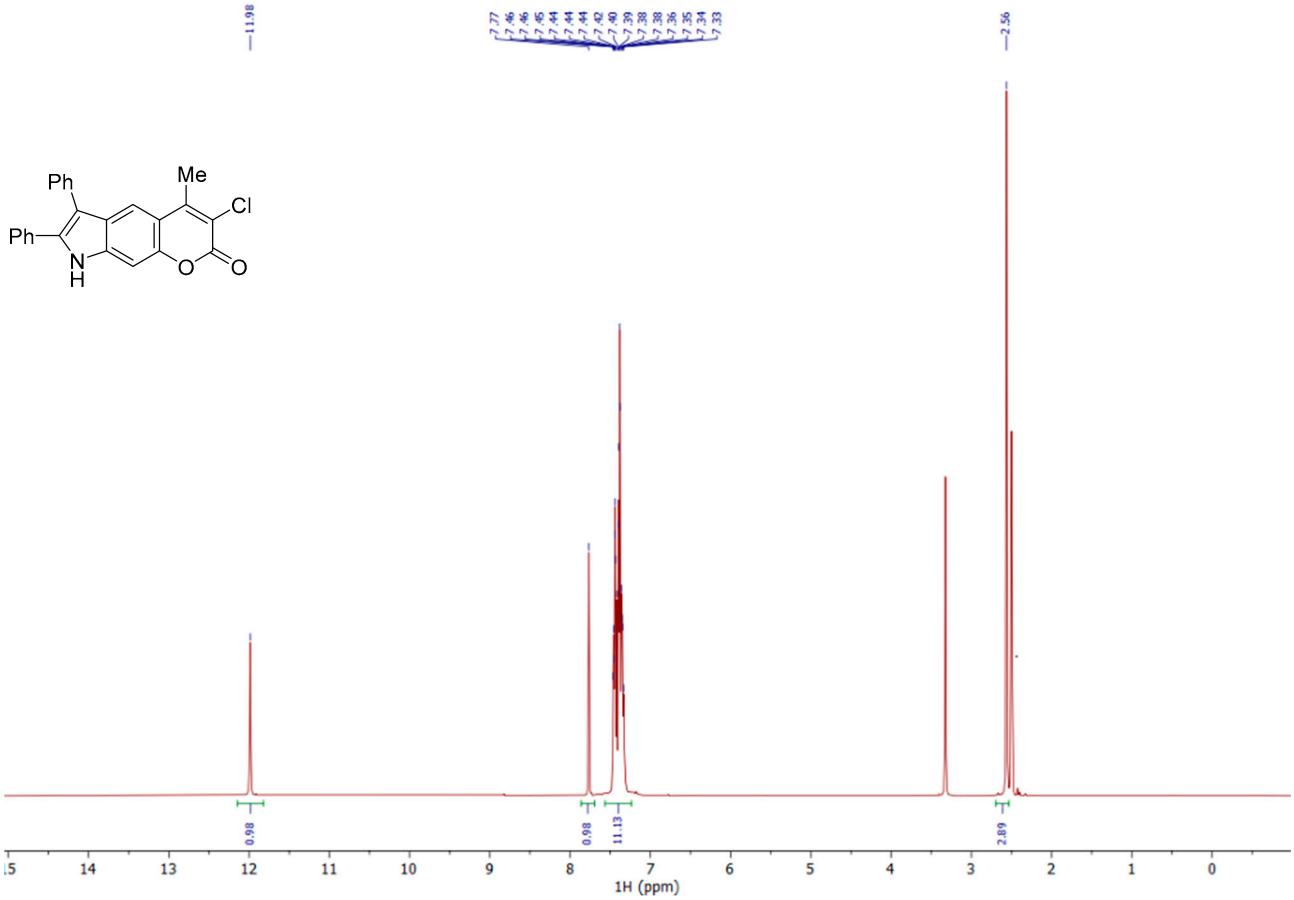

Figure S25. ^{1}H NMR spectrum of **7e**.

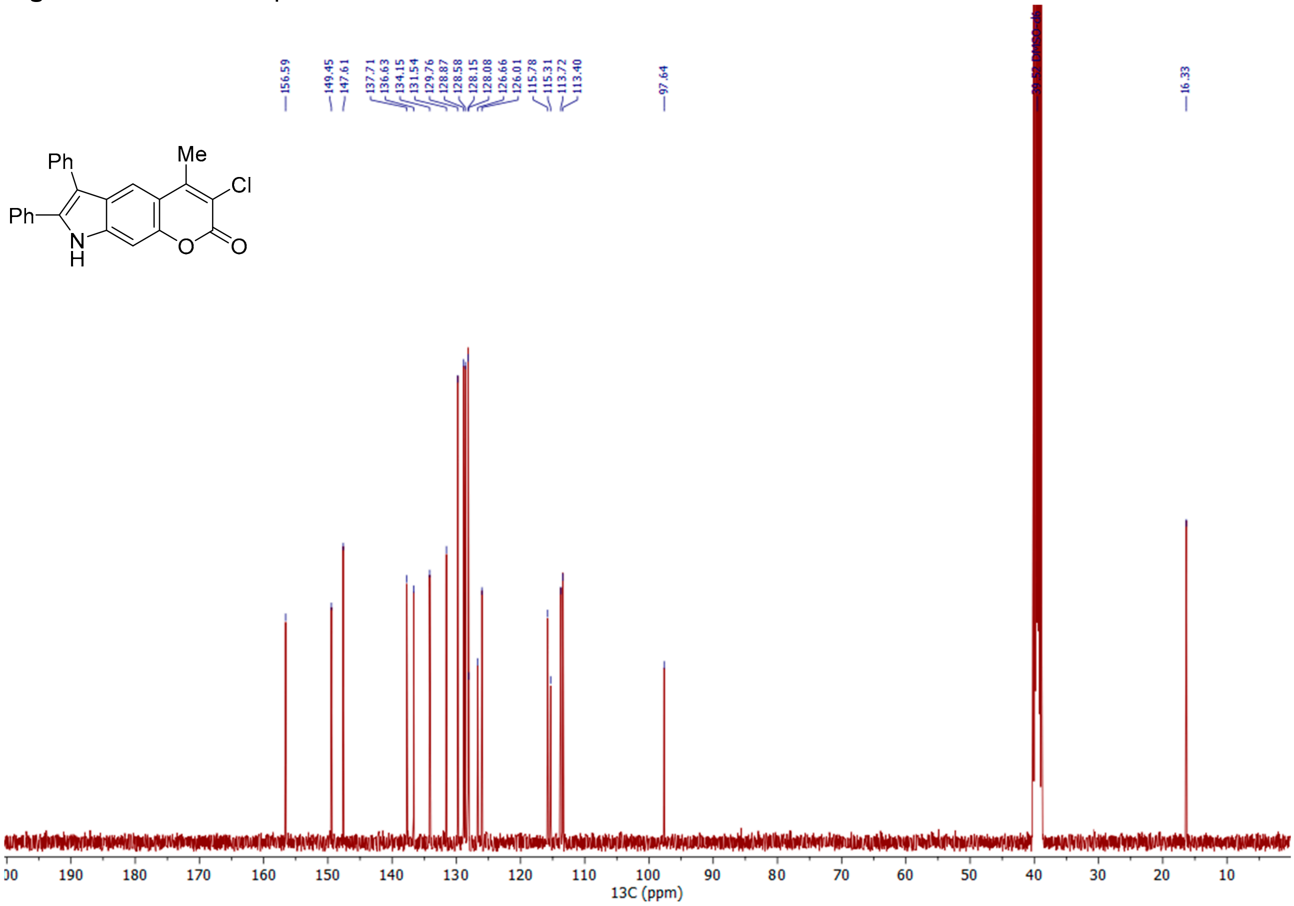

Figure S26. ^{13}C NMR spectrum of **7e**.

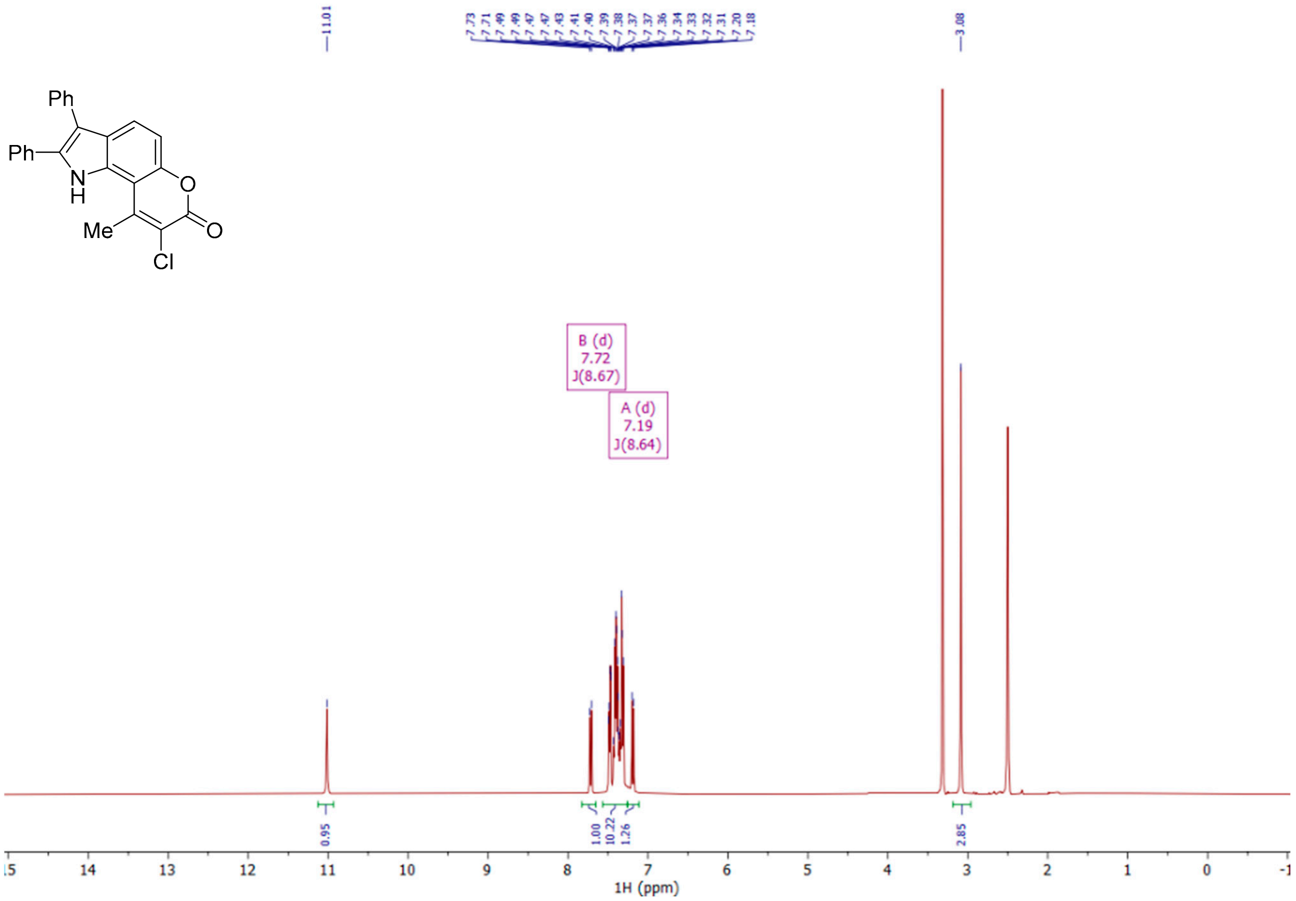

Figure S27. ^{1}H NMR spectrum of **8e**.

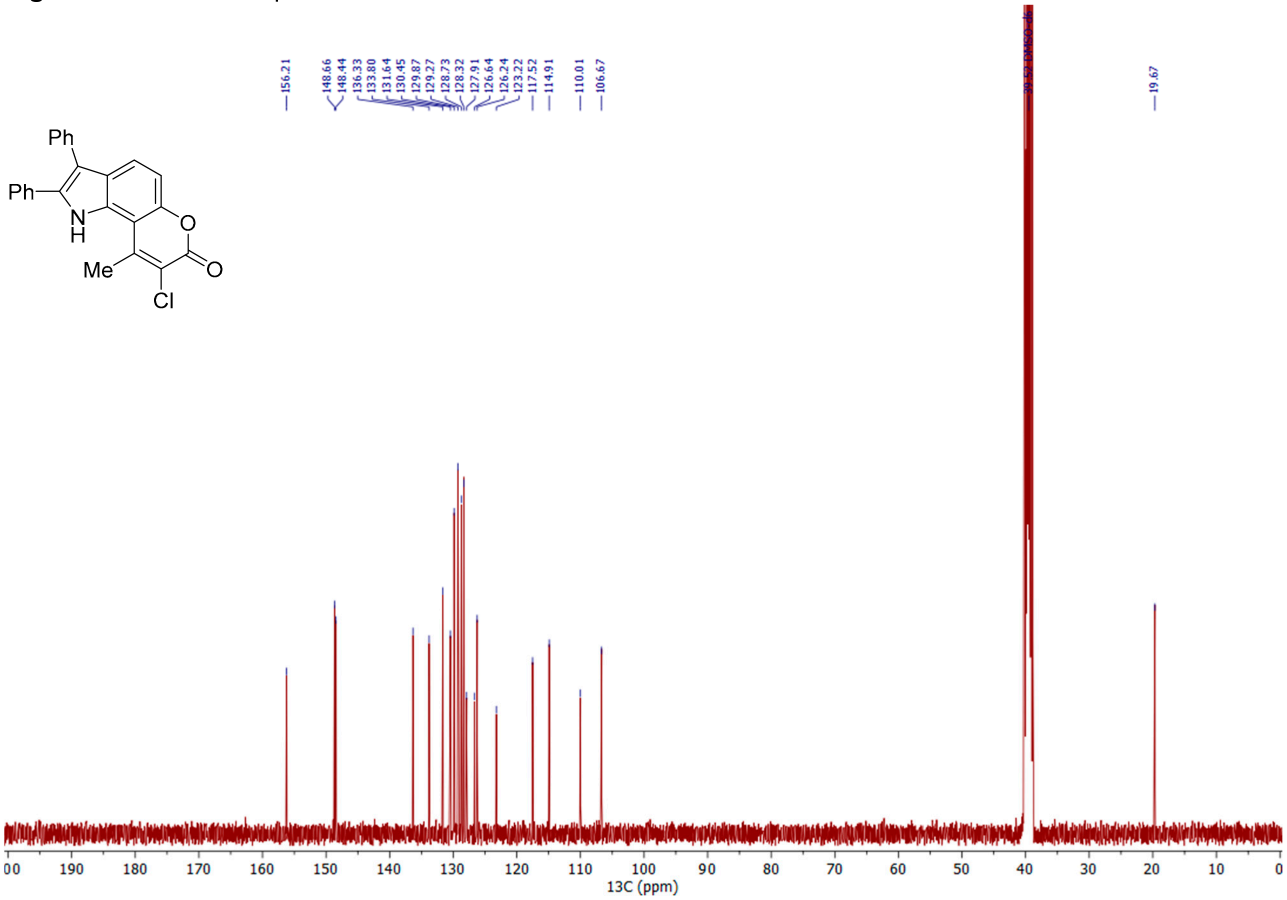

Figure S28. ^{13}C NMR spectrum of **8e**.

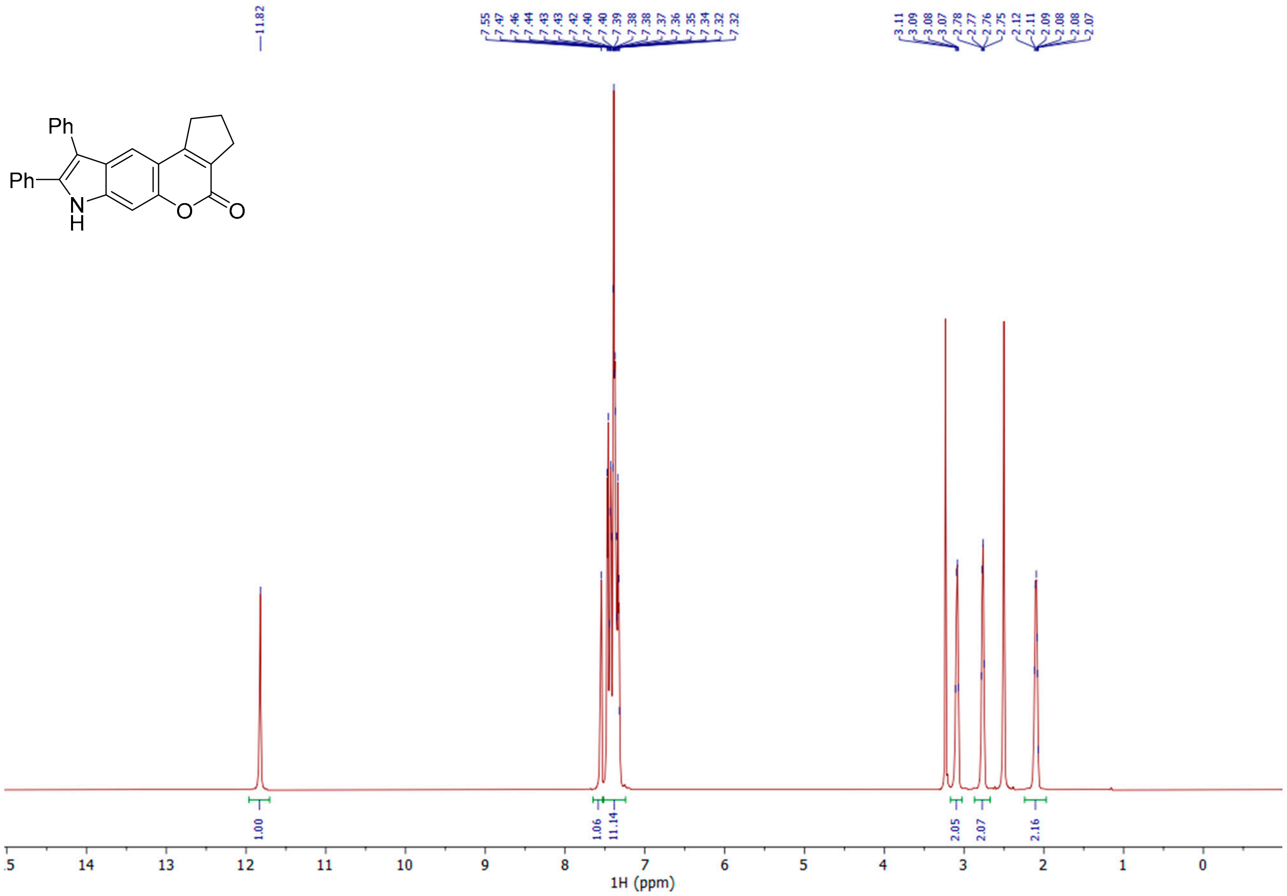

Figure S29. ^{1}H NMR spectrum of **7f**.

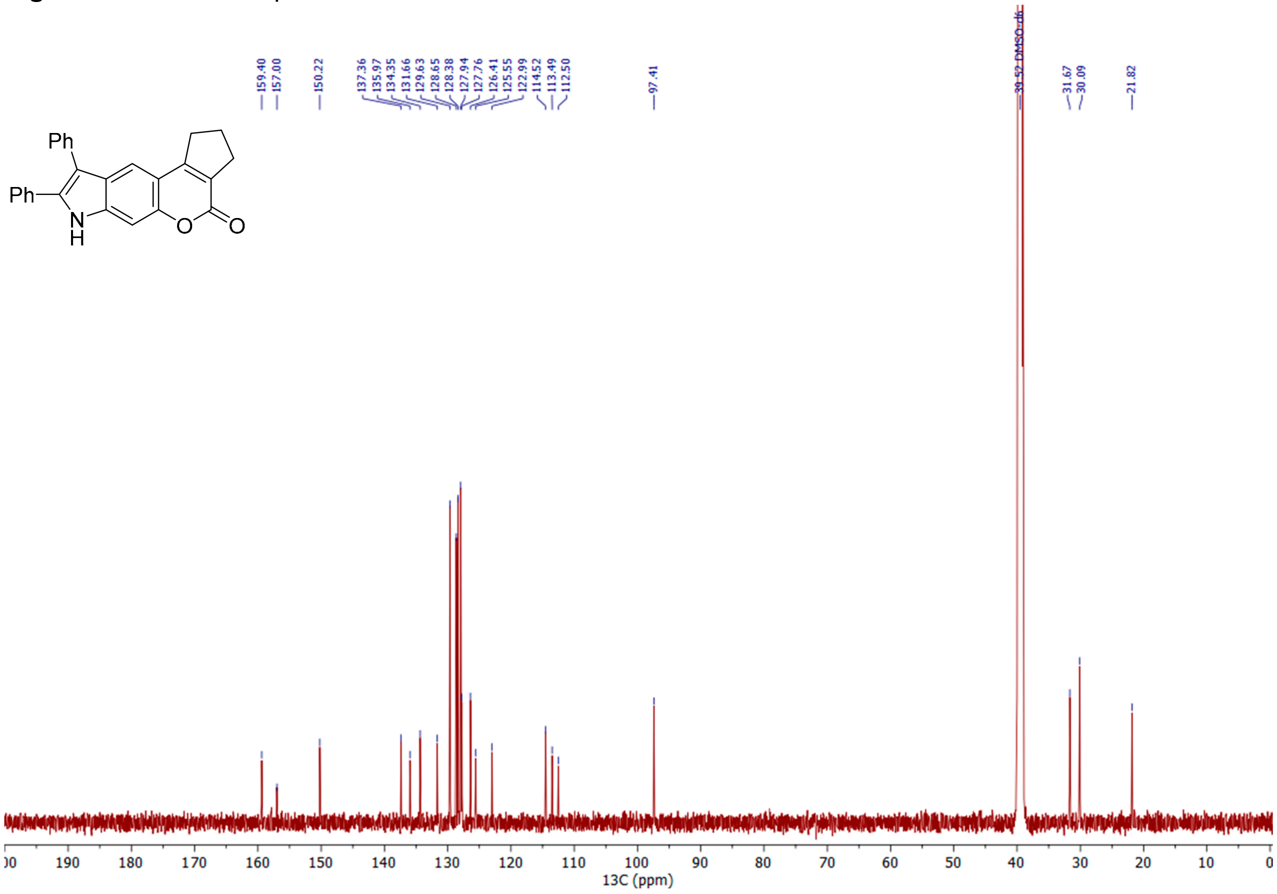

Figure S30. ^{13}C NMR spectrum of **7f**.

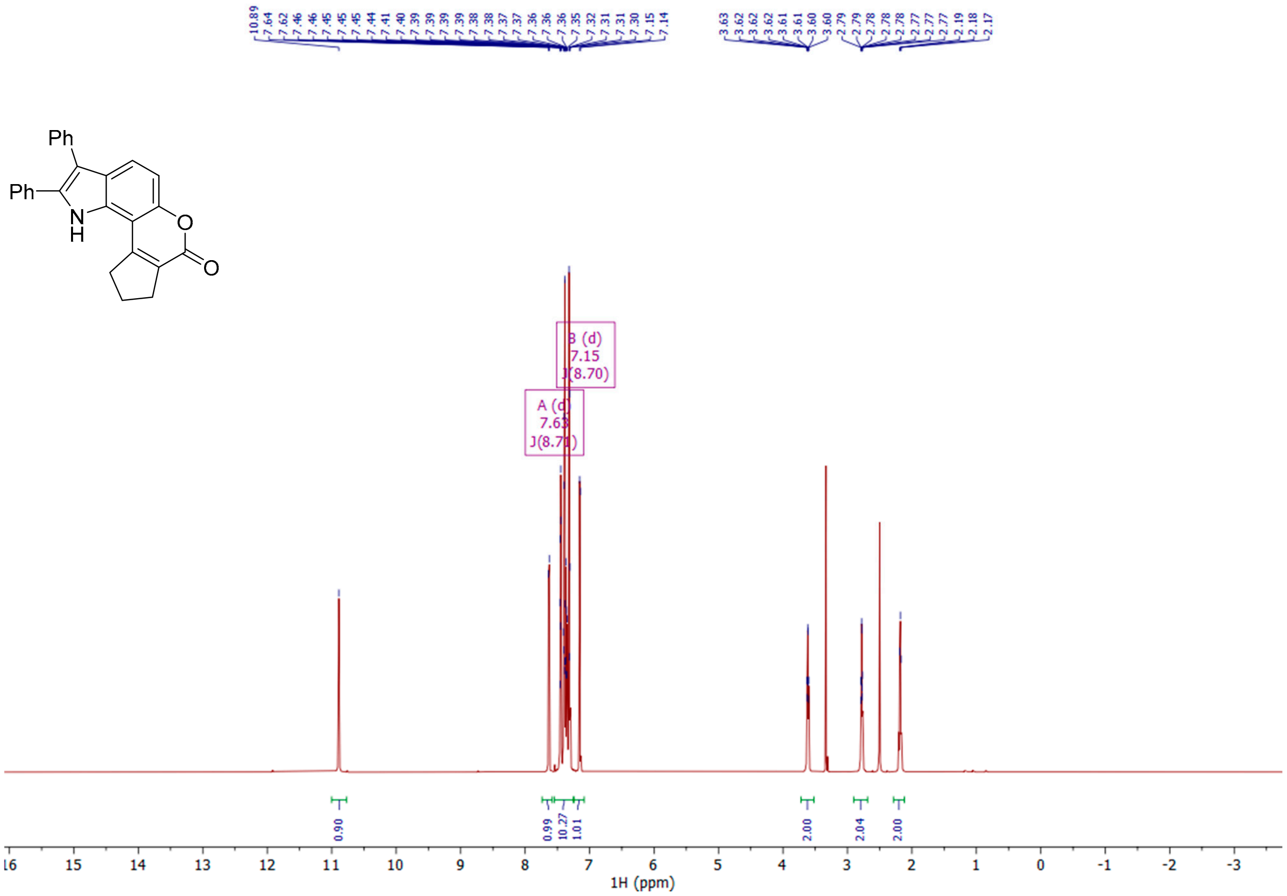

Figure S31. ^{1}H NMR spectrum of **8f**.

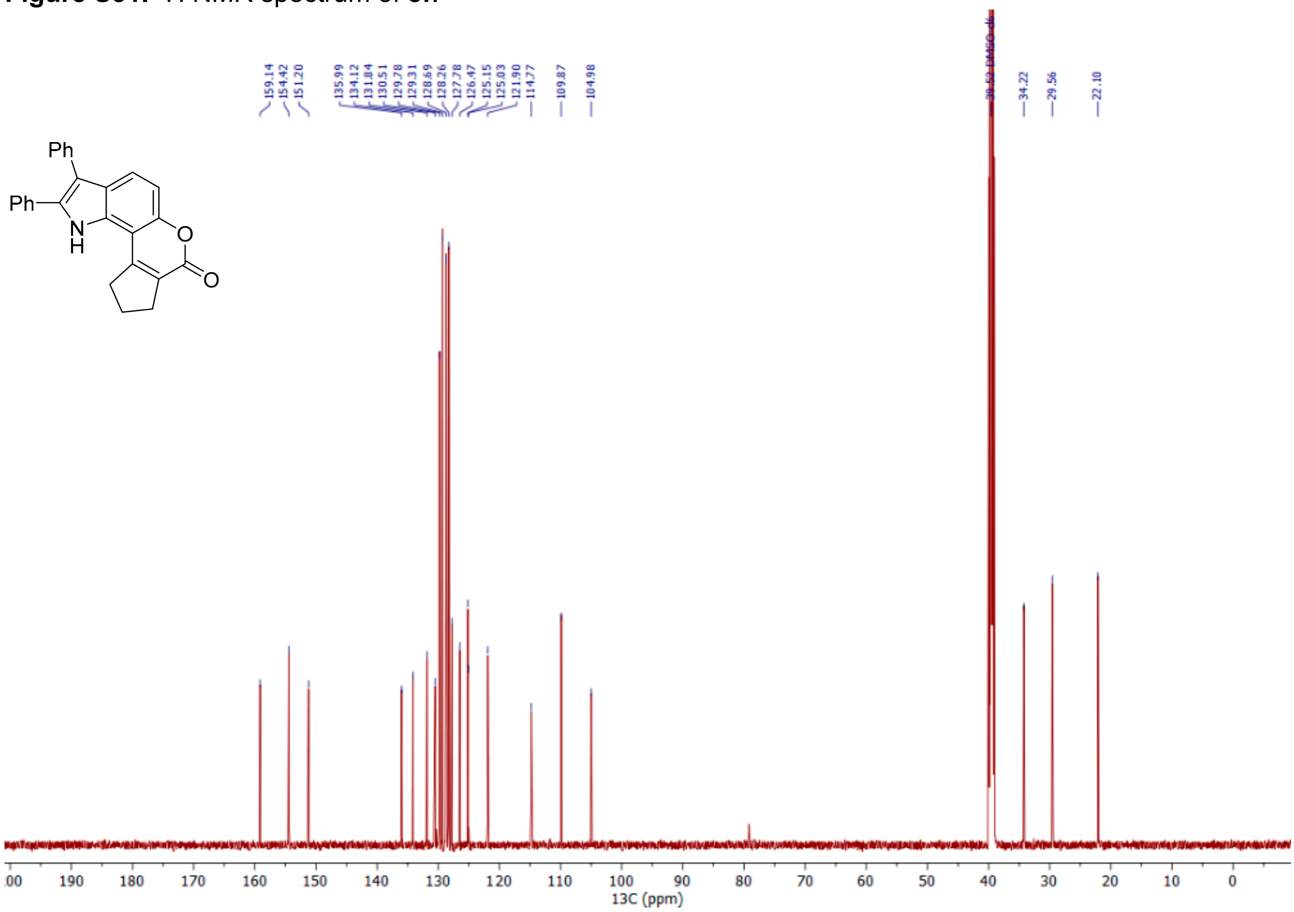

Figure S32. ^{13}C NMR spectrum of **8f**.

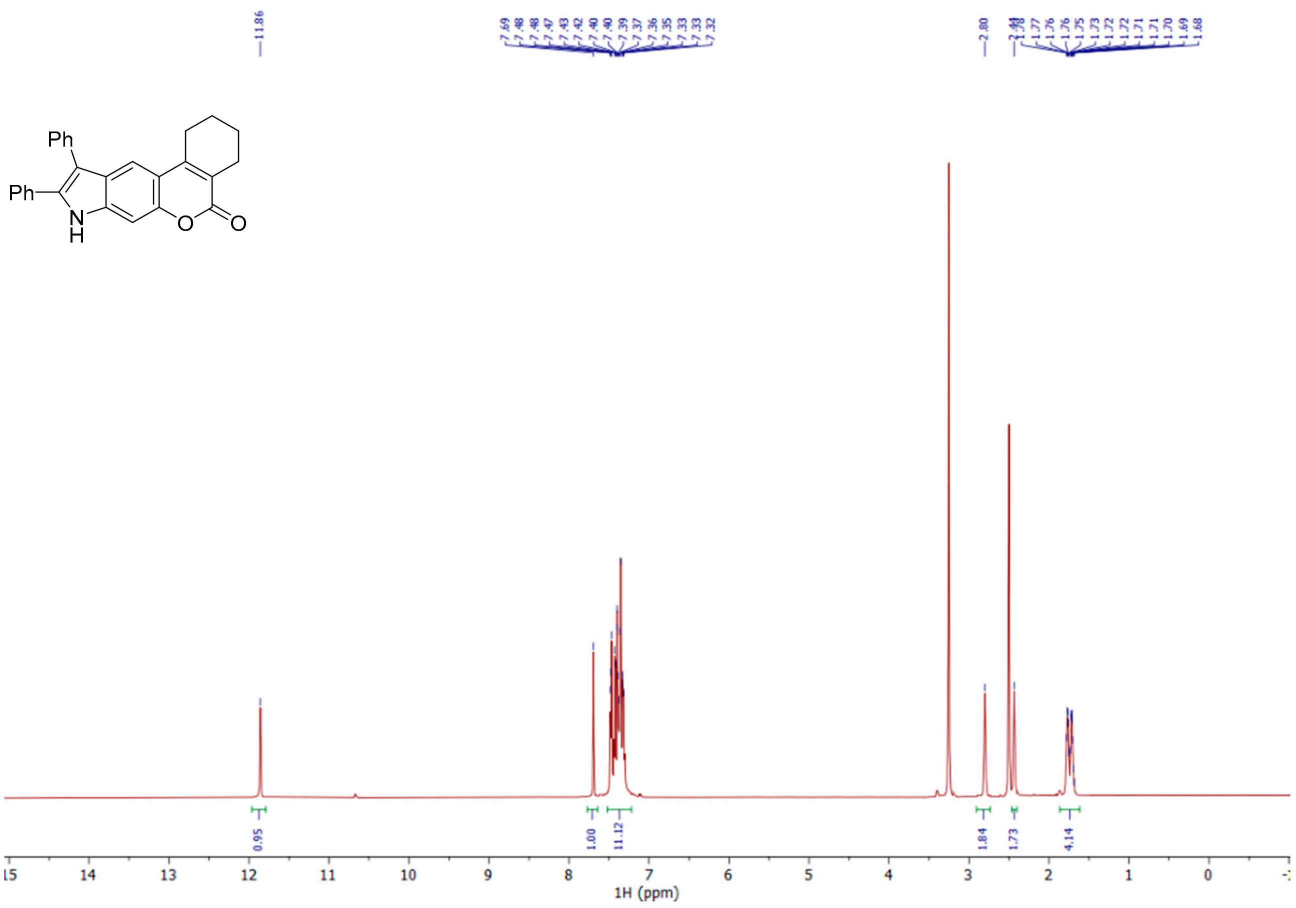

Figure S33. ^{1}H NMR spectrum of **7g**.

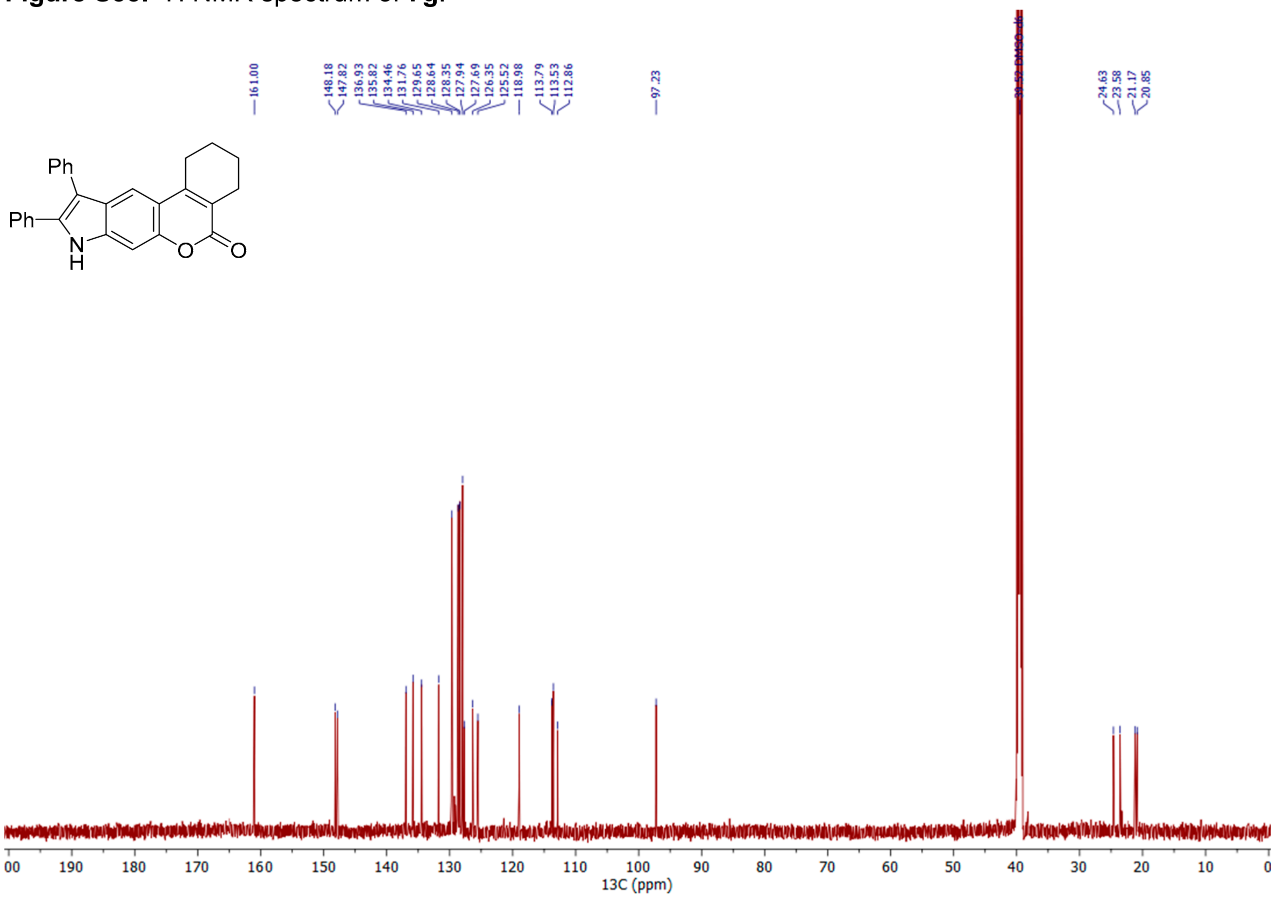

Figure S34. ^{13}C NMR spectrum of **7g**.

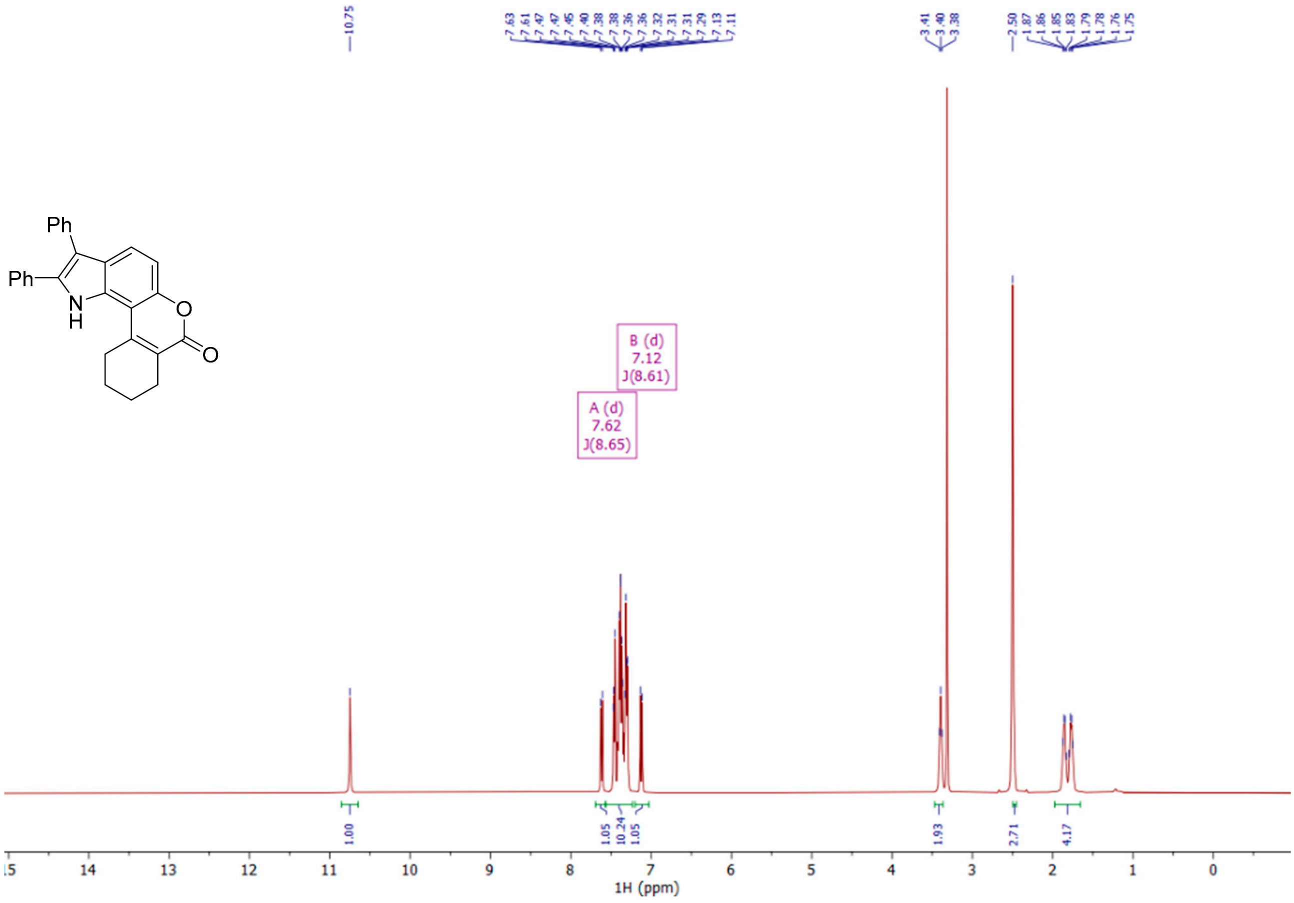

Figure S35. ^{1}H NMR spectrum of **8g**.

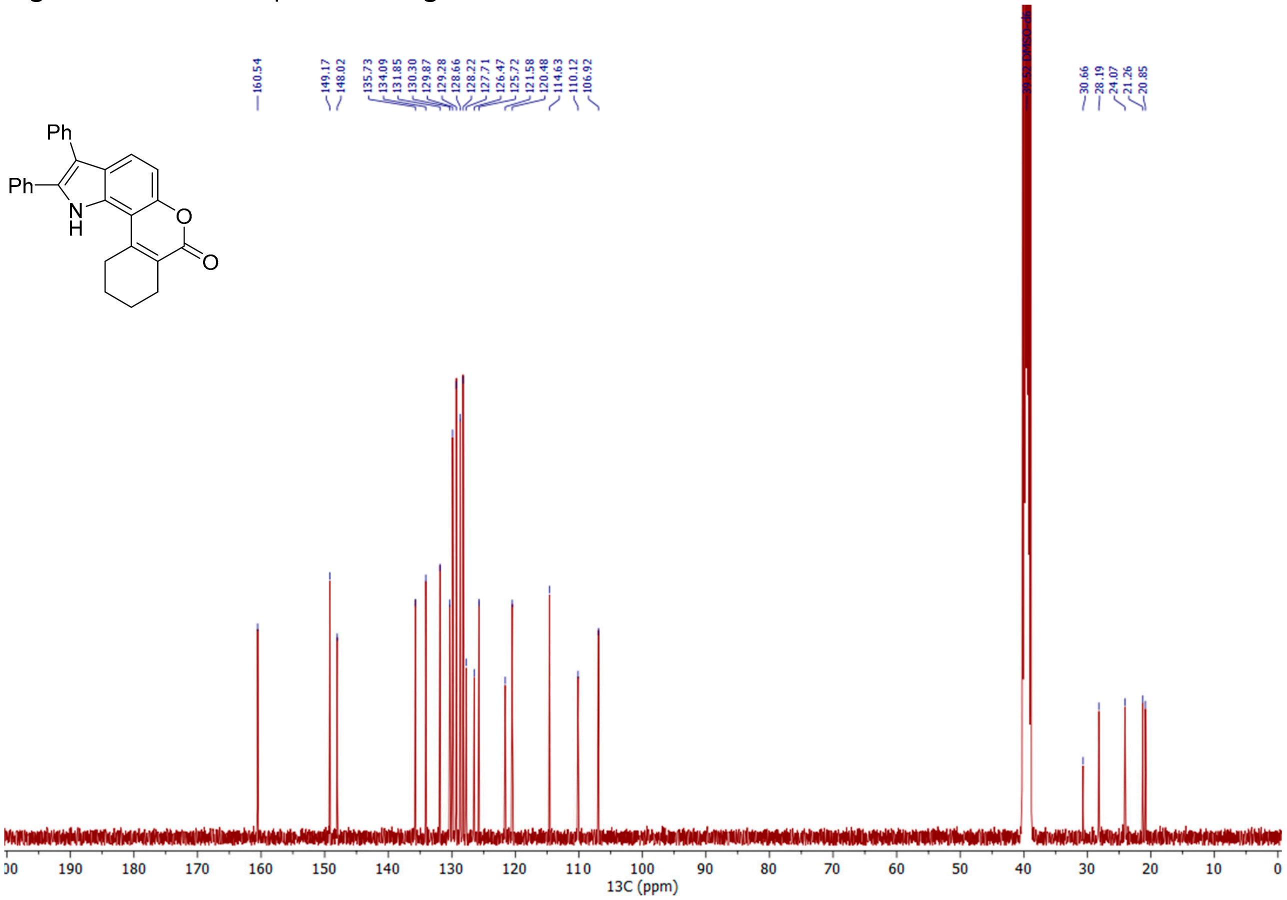

Figure S36. ^{13}C NMR spectrum of **8g**.

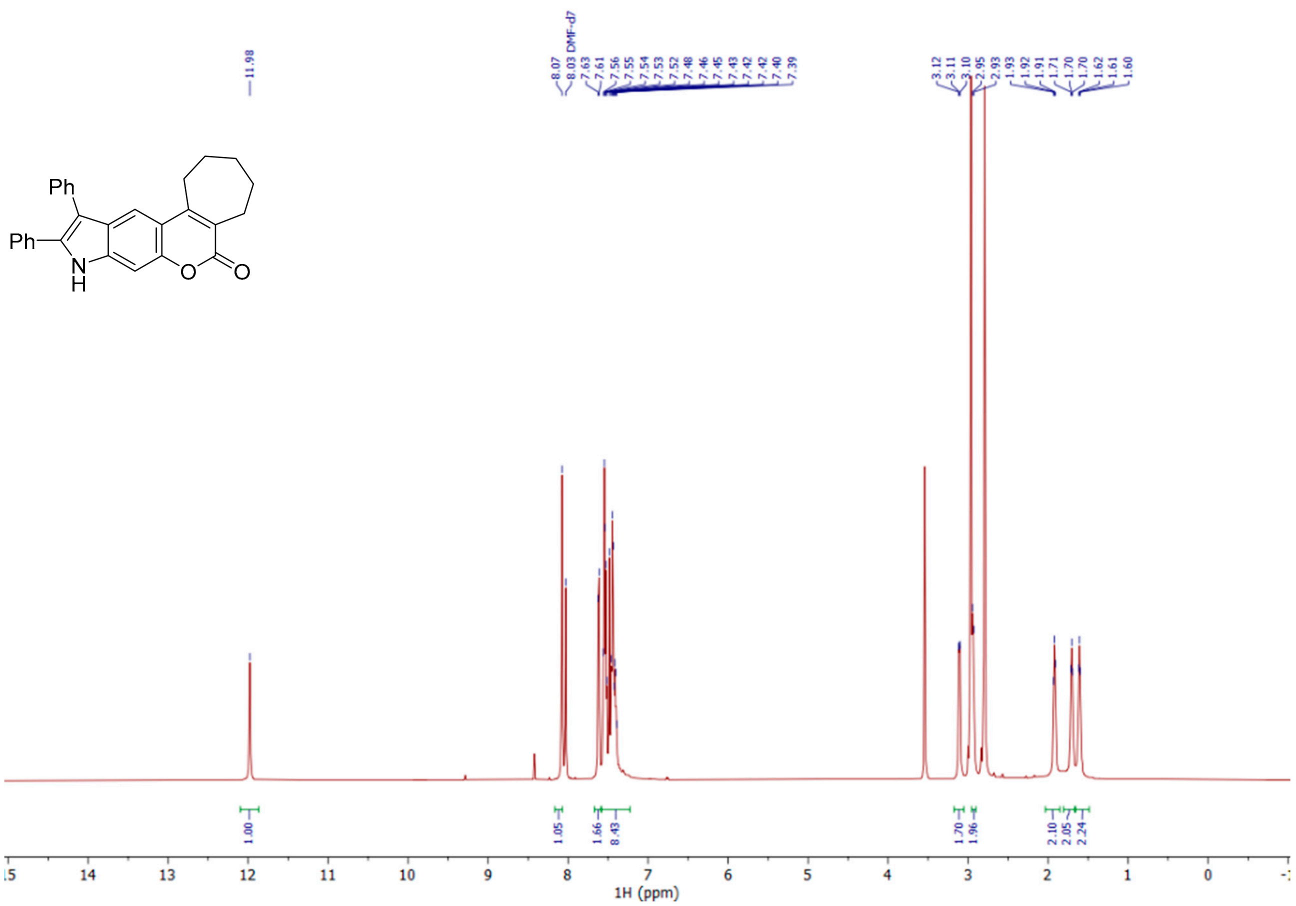

Figure S37. ^{1}H NMR spectrum of **7h**.

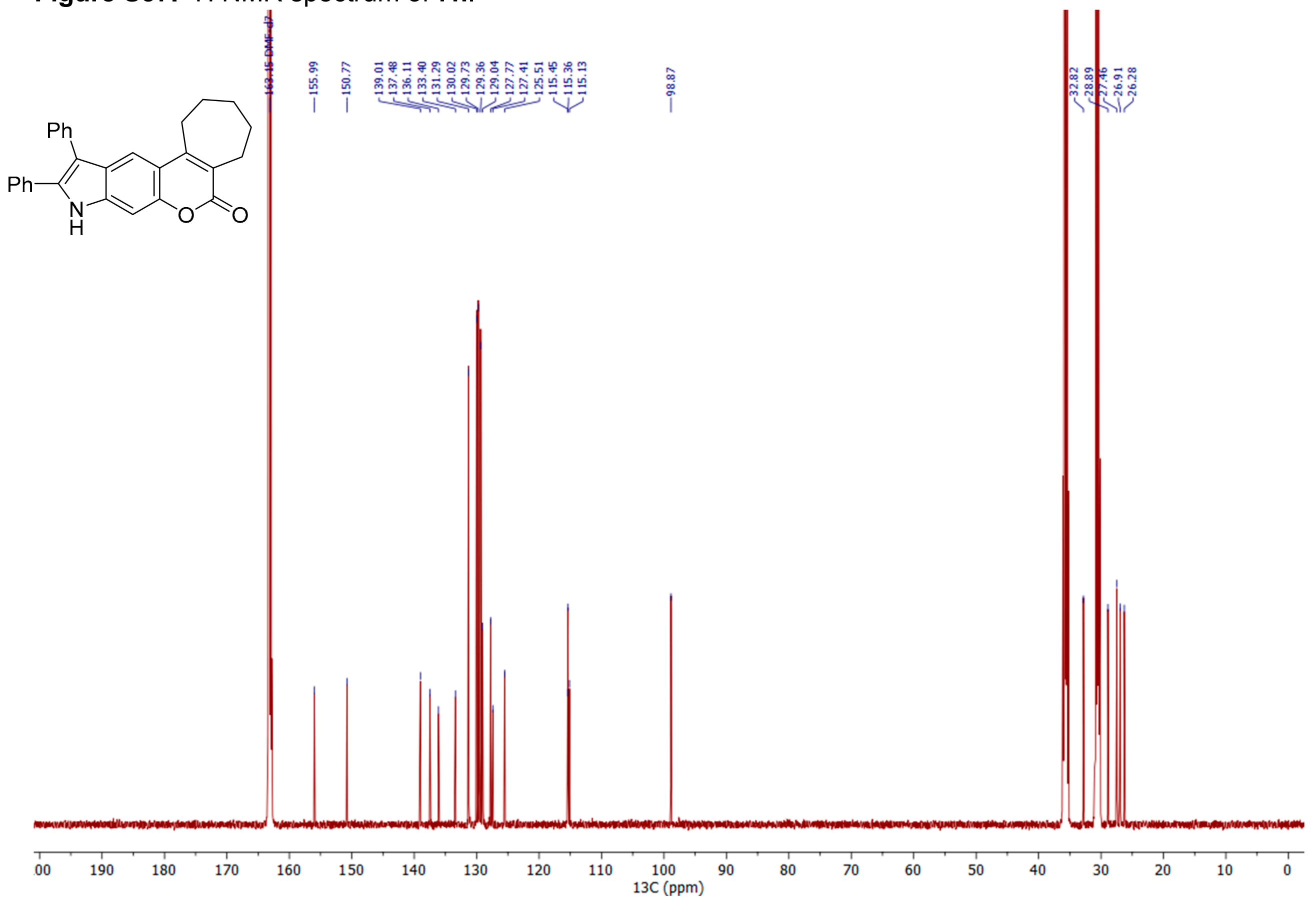

Figure S38. ^{13}C NMR spectrum of **7h**.

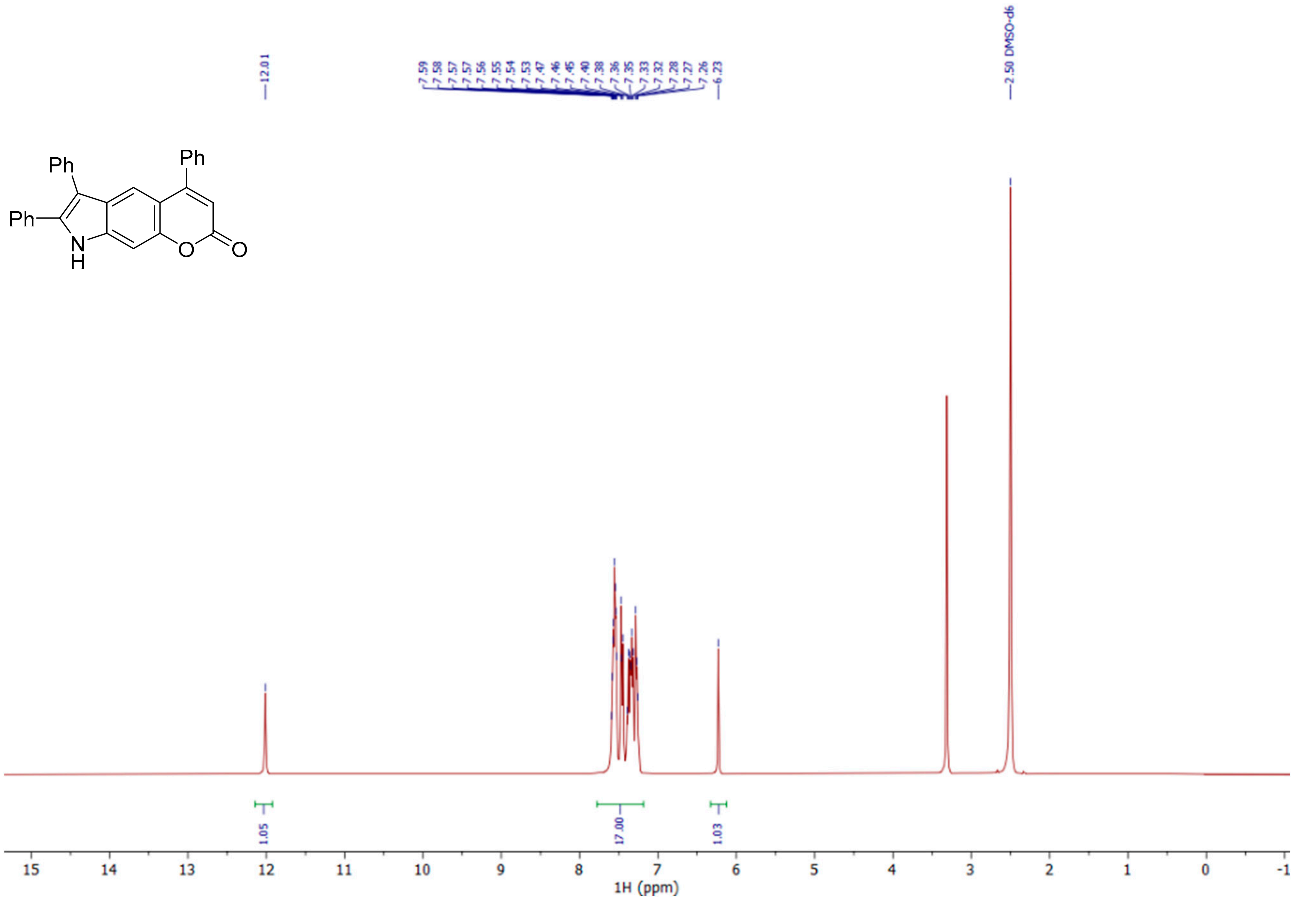

Figure S39. ^{1}H NMR spectrum of **7i**.

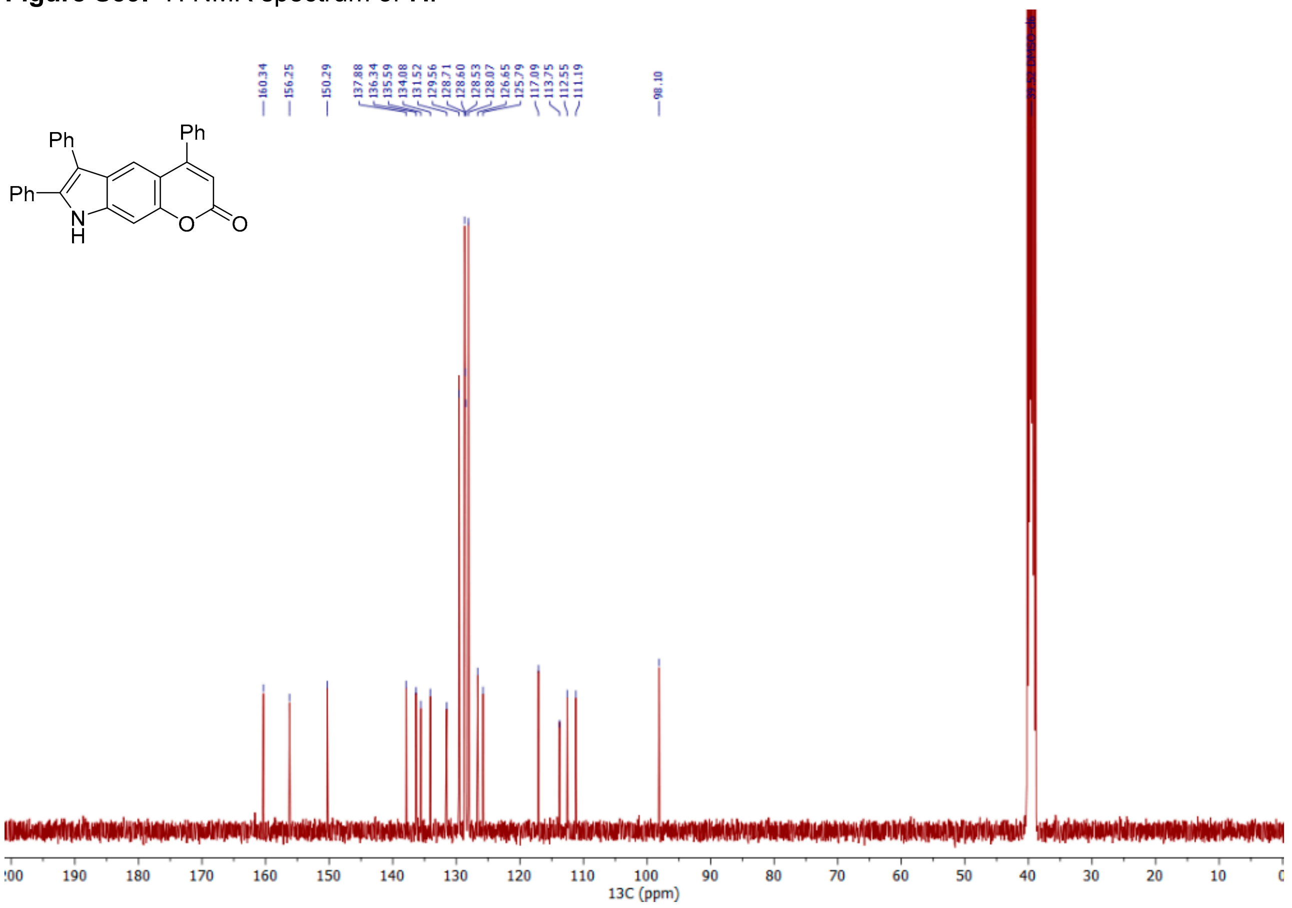

Figure S40. ^{13}C NMR spectrum of **7i**.

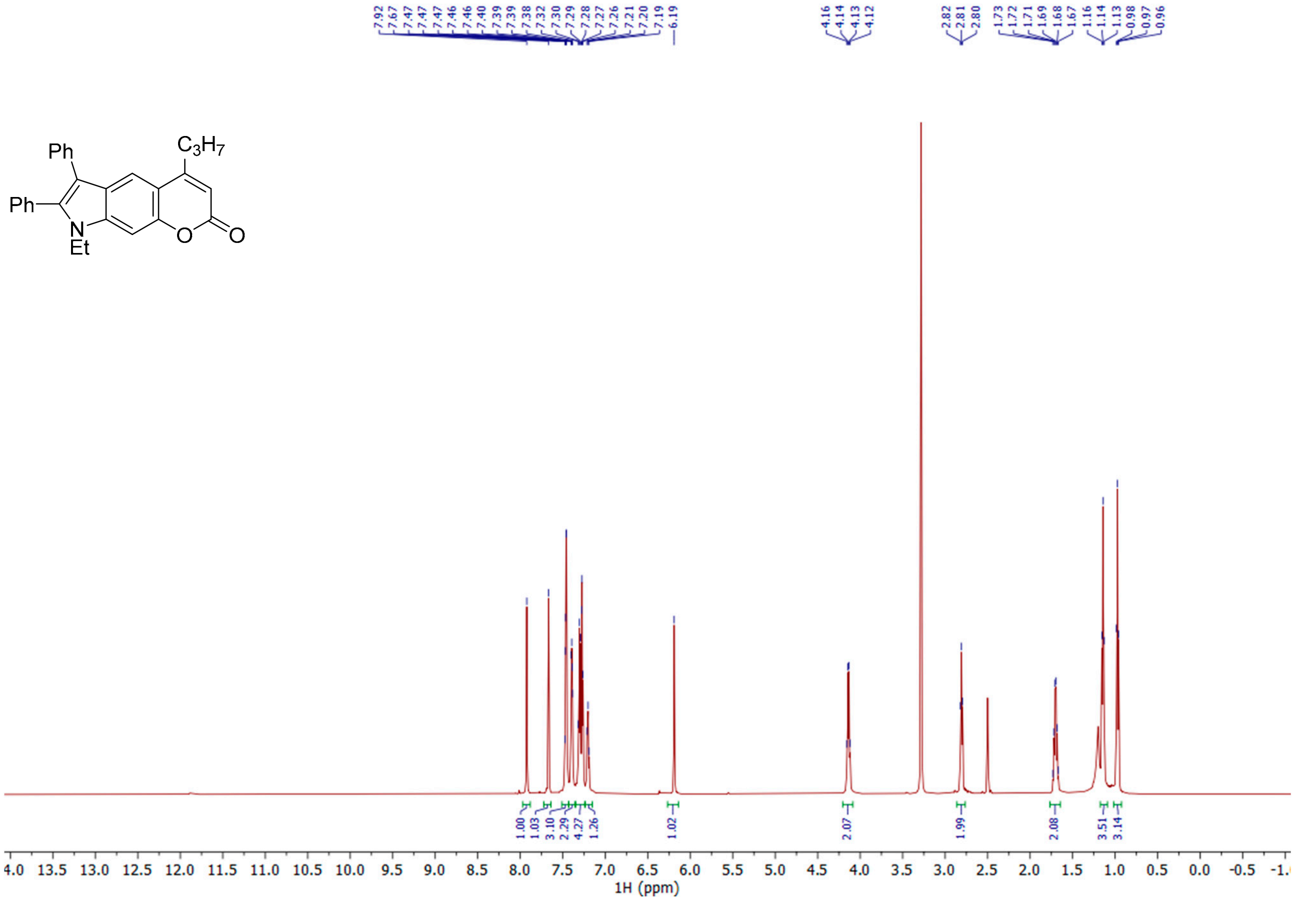

Figure S41. ^{1}H NMR spectrum of **9a**.

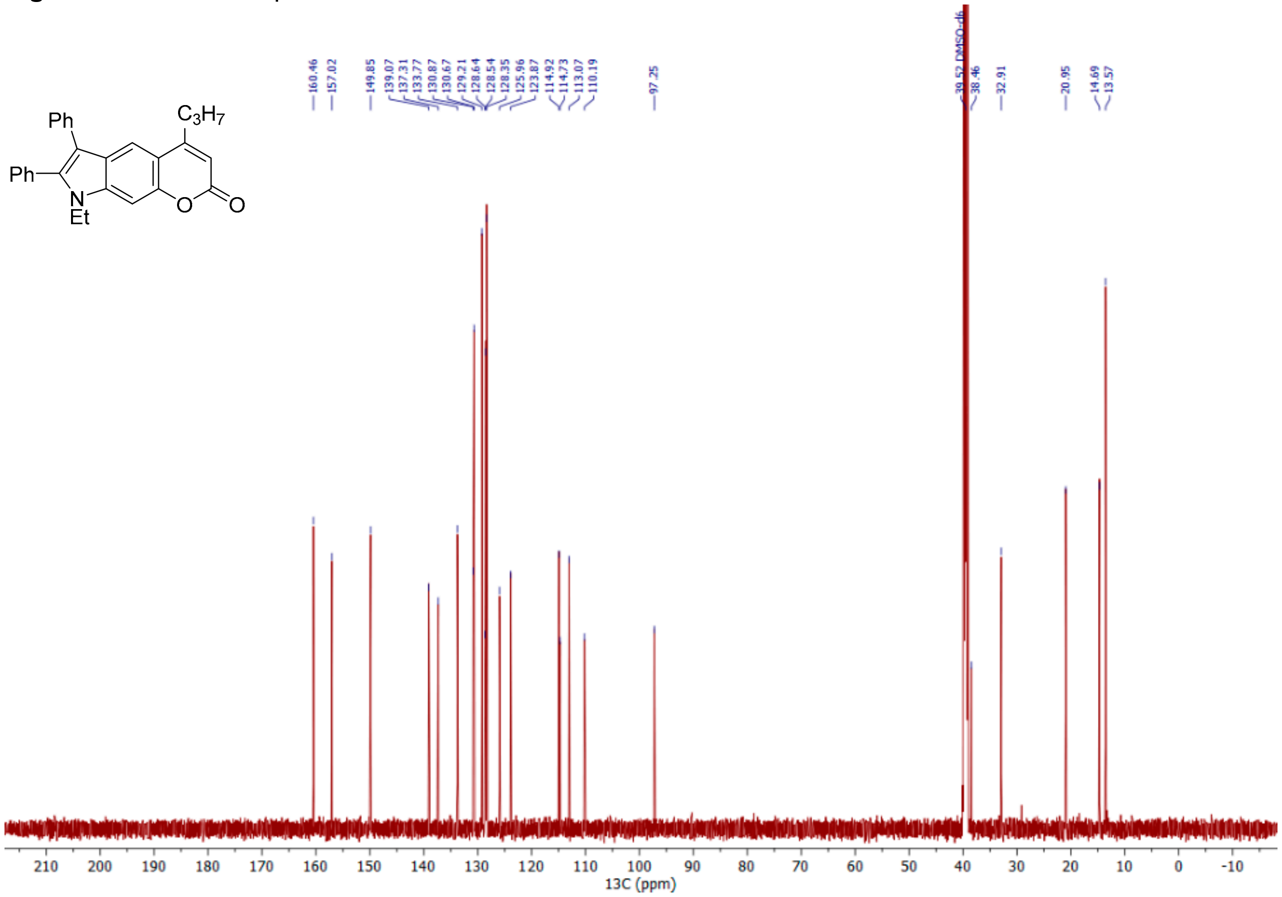

Figure S42. ^{13}C NMR spectrum of **9a**.

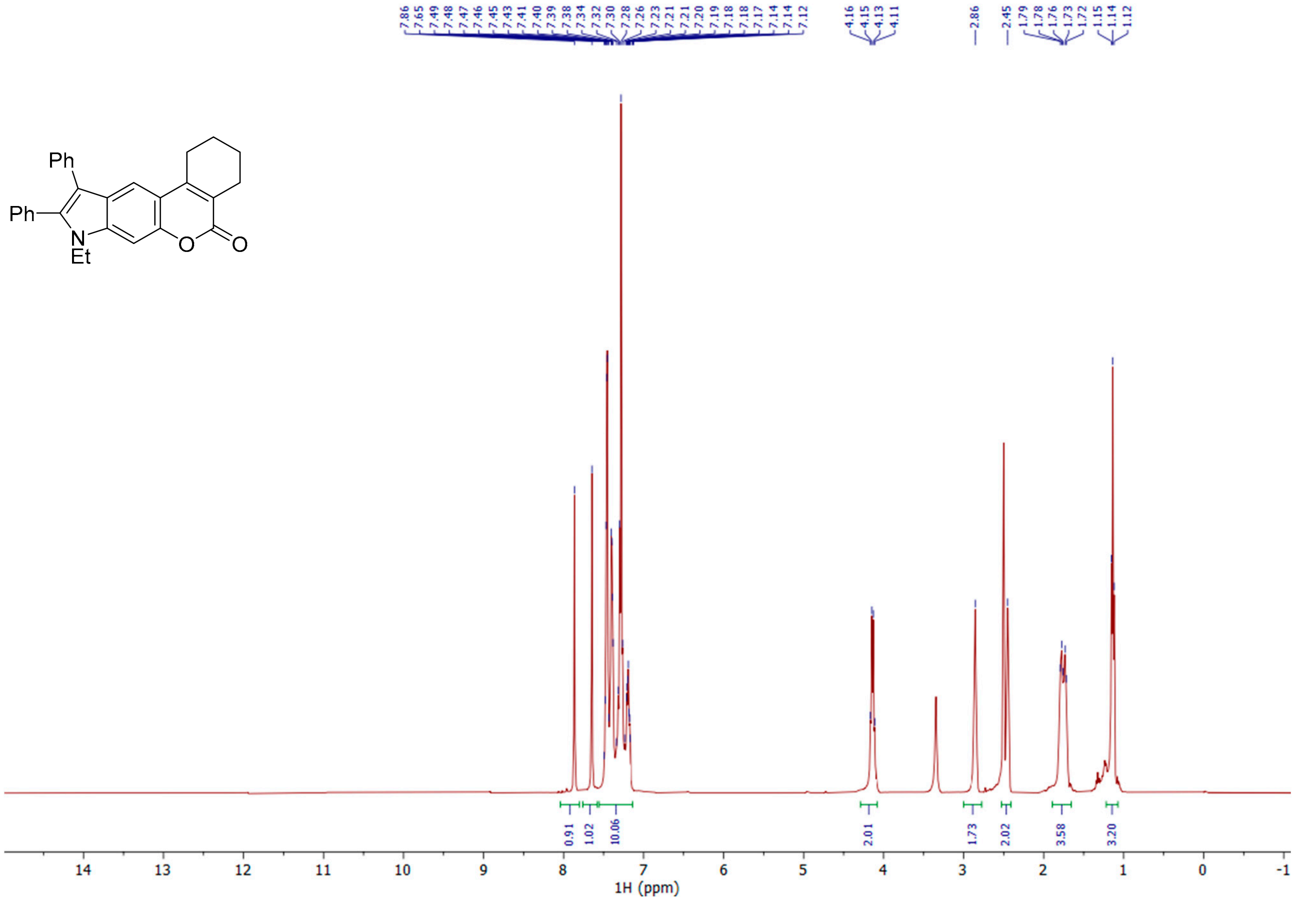

Figure S43. ^{1}H NMR spectrum of **9b**.

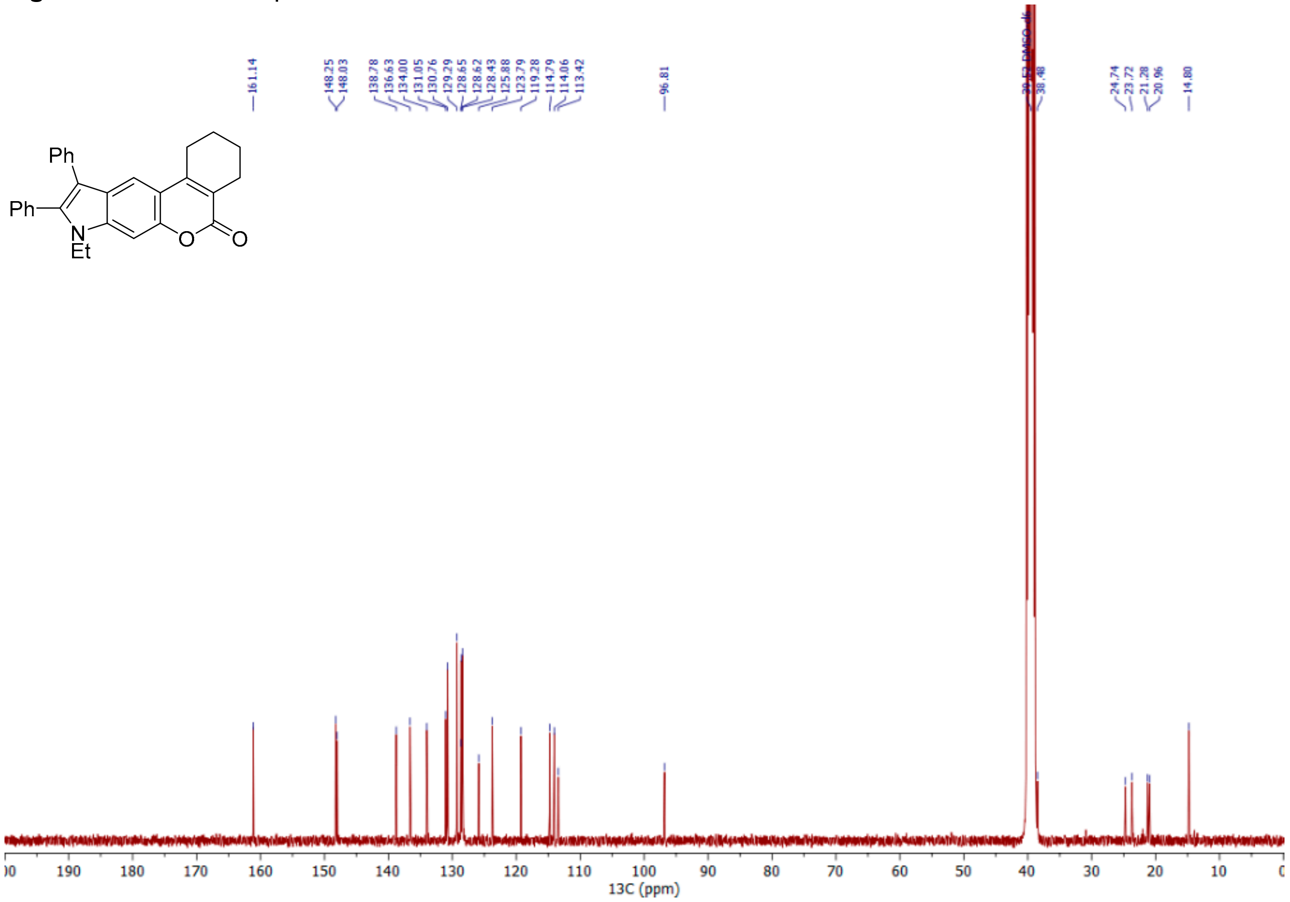

Figure S44. ^{13}C NMR spectrum of **9b**.

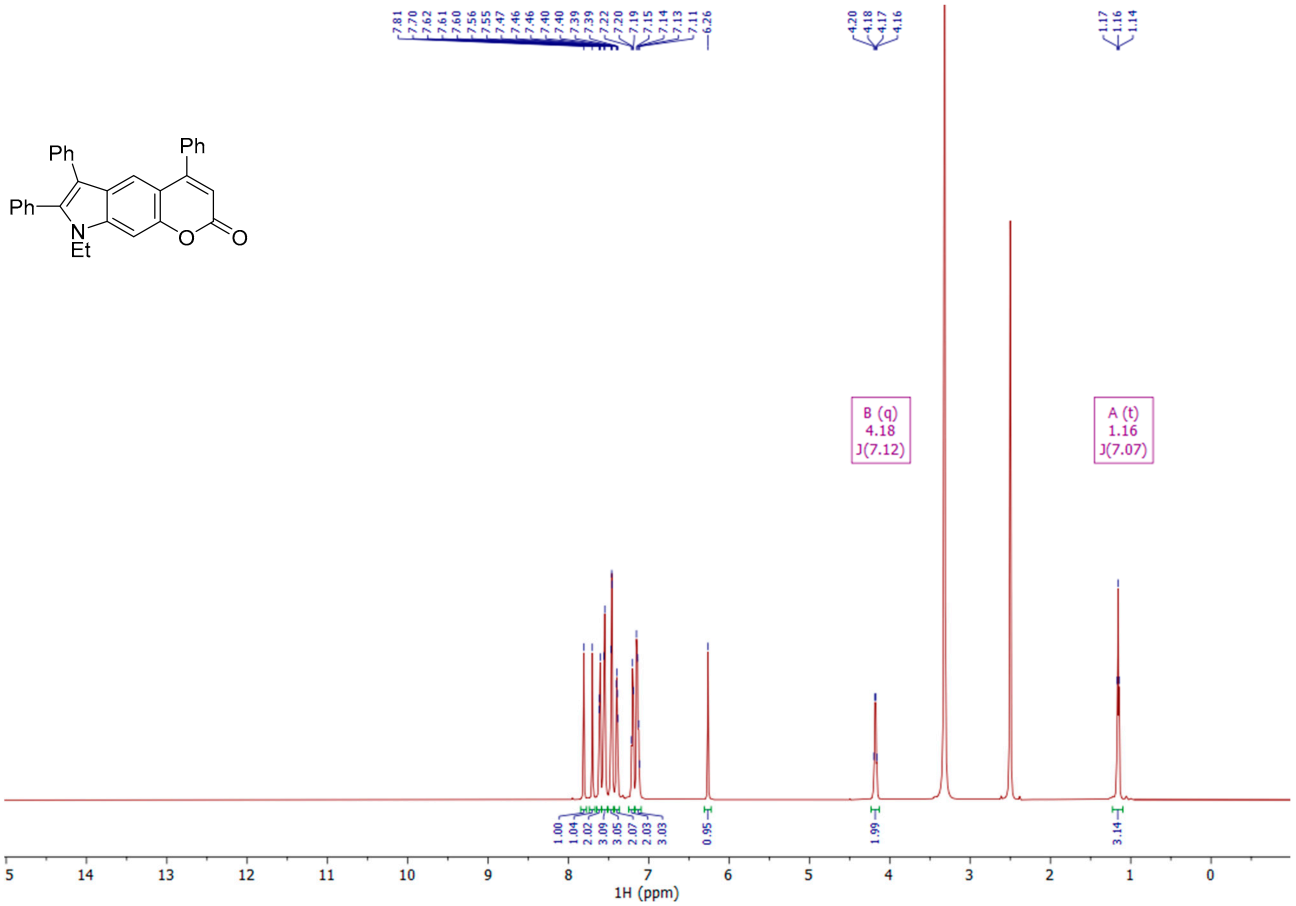

Figure S45. ^{1}H NMR spectrum of **9c**.

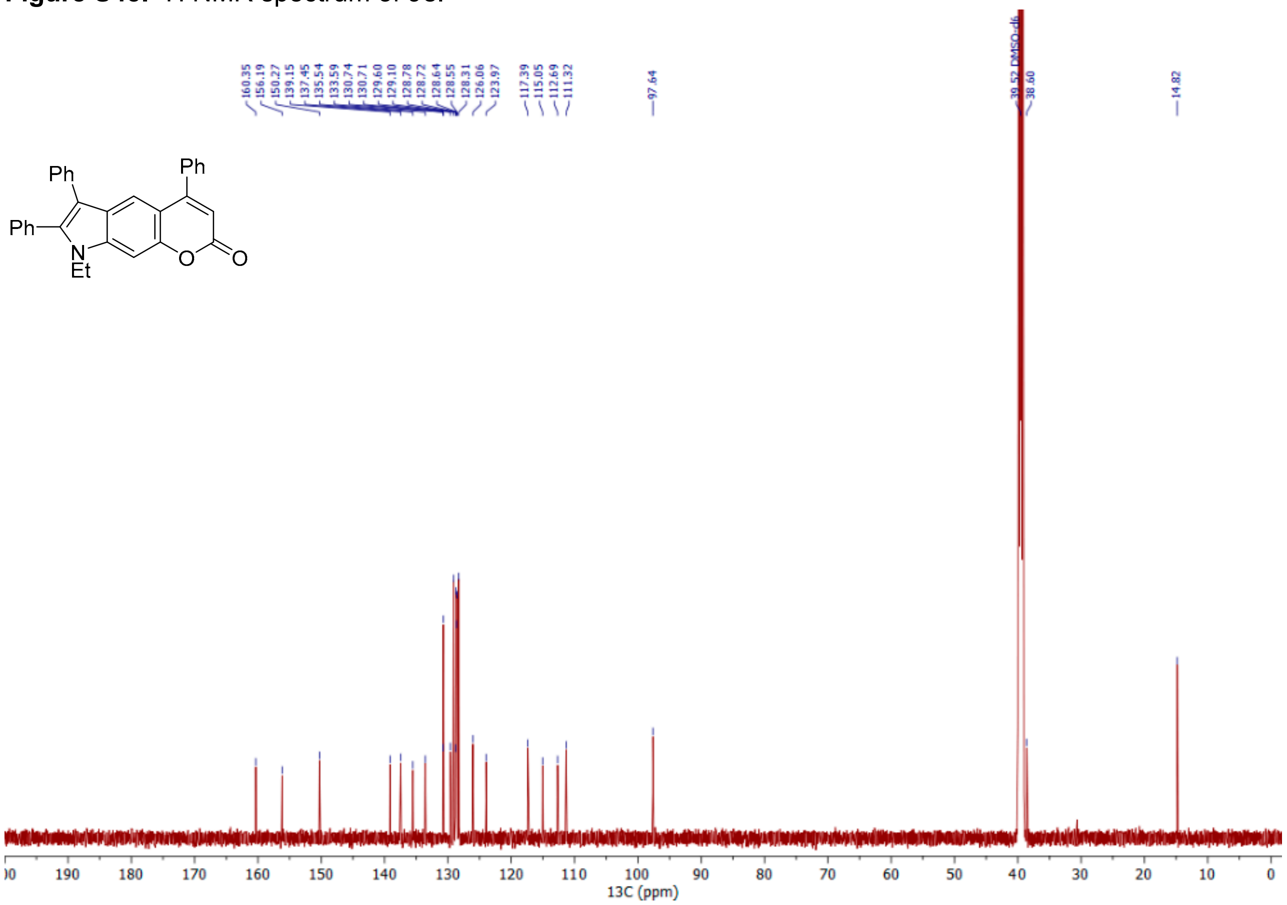

Figure S46. ^{13}C NMR spectrum of **9c**.

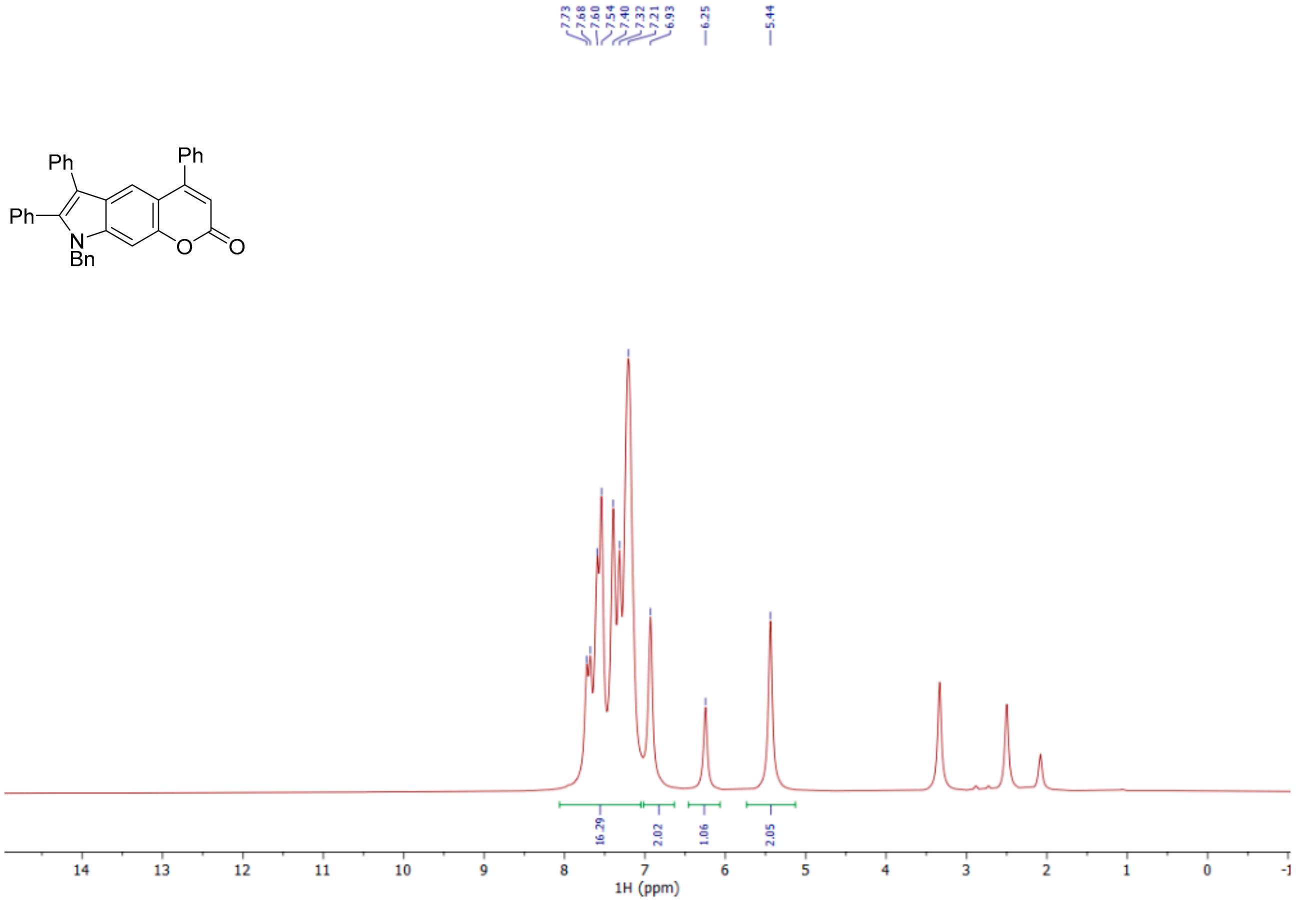

Figure S47. ^{1}H NMR spectrum of **9d.**

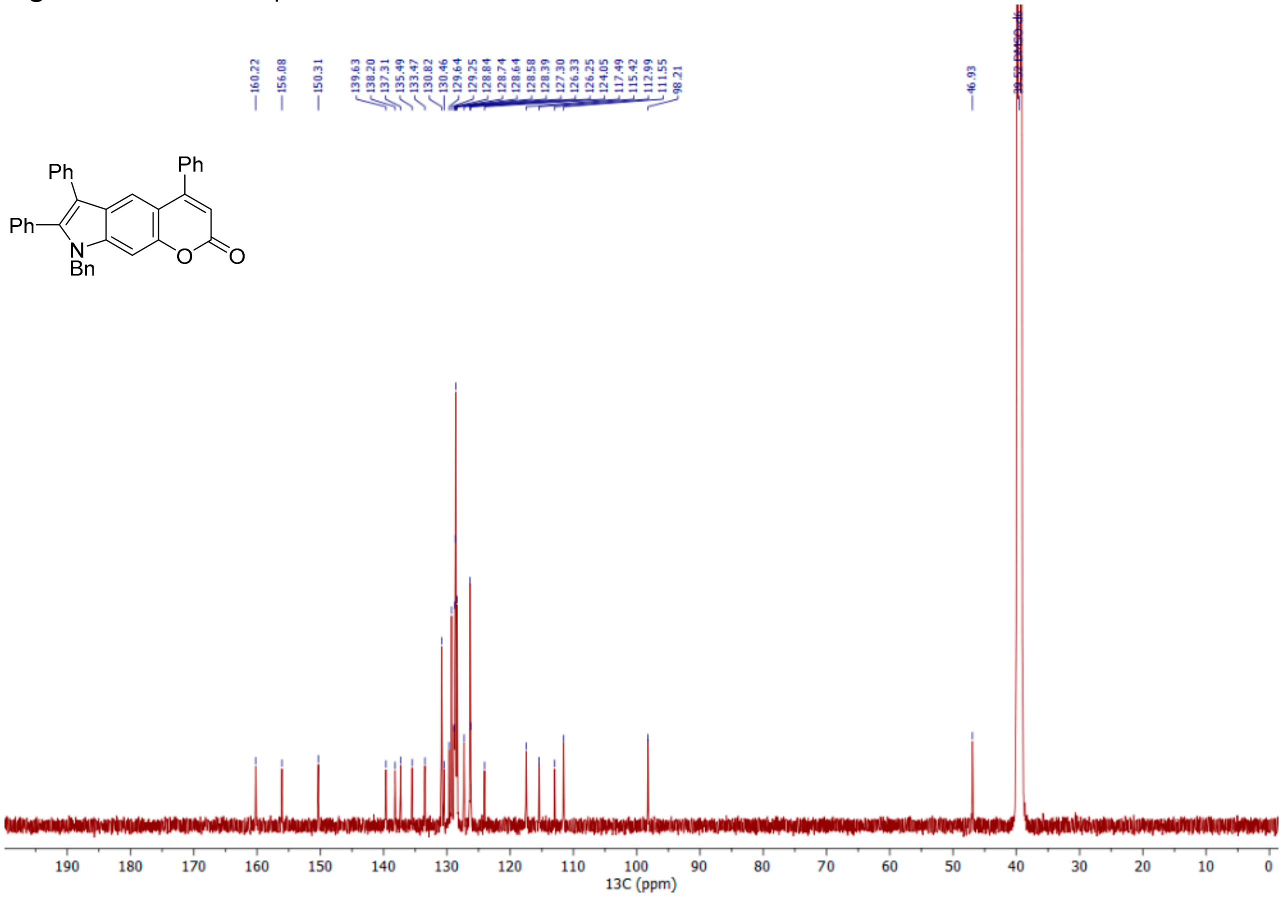

Figure S48. ^{13}C NMR spectrum of **9d.**

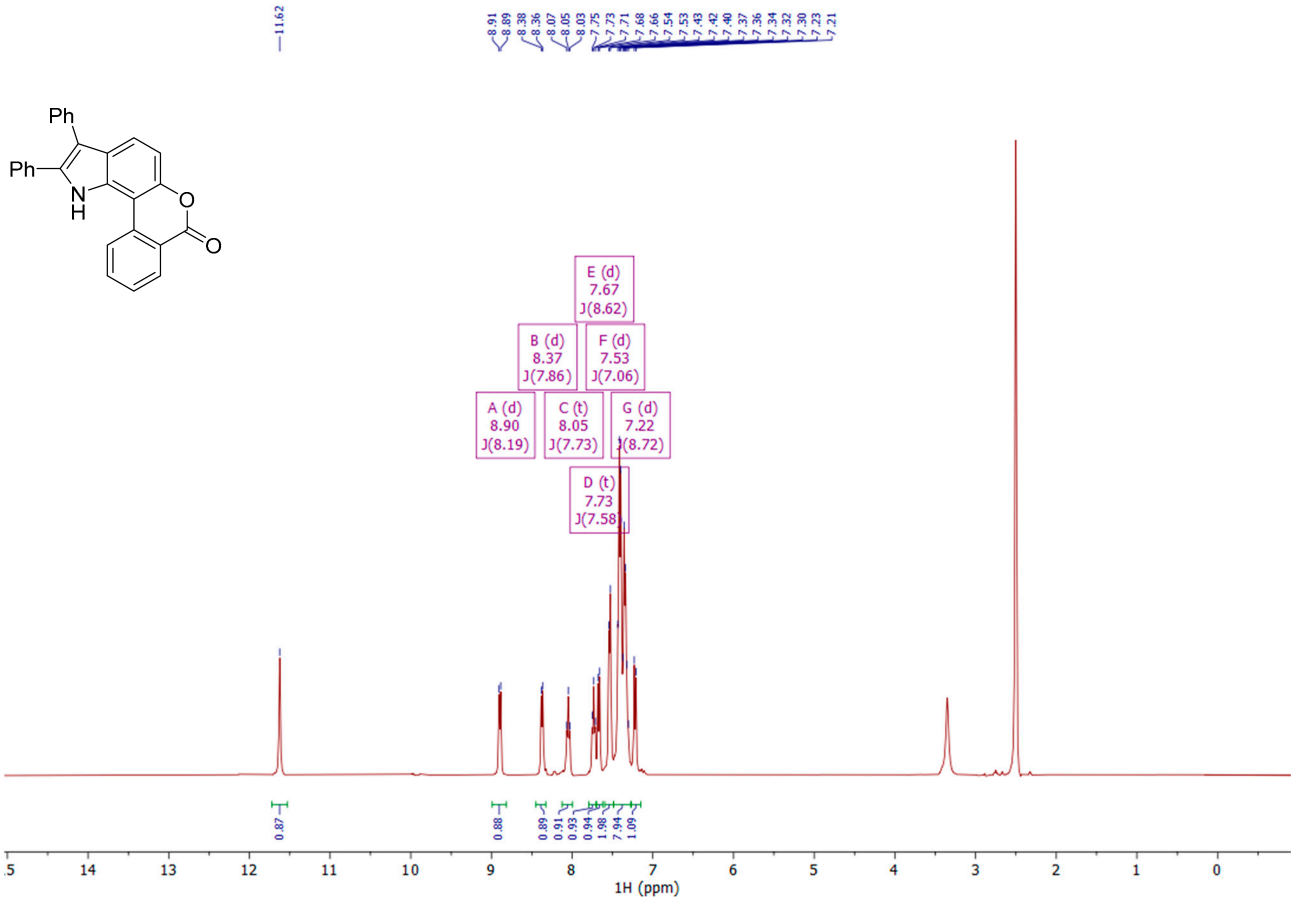

Figure S49. ^{1}H NMR spectrum of **10**.

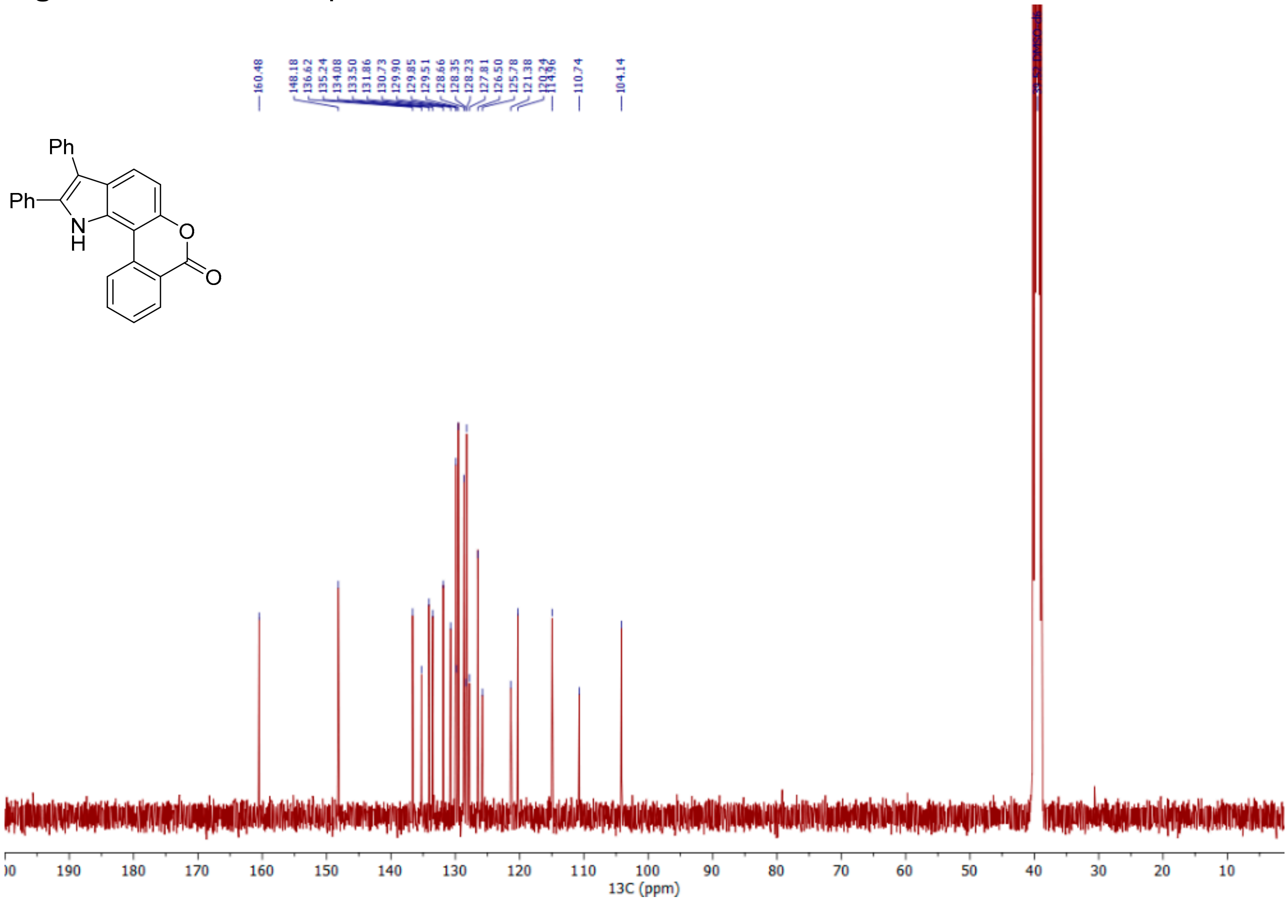

Figure S50. ^{13}C NMR spectrum of **10**.

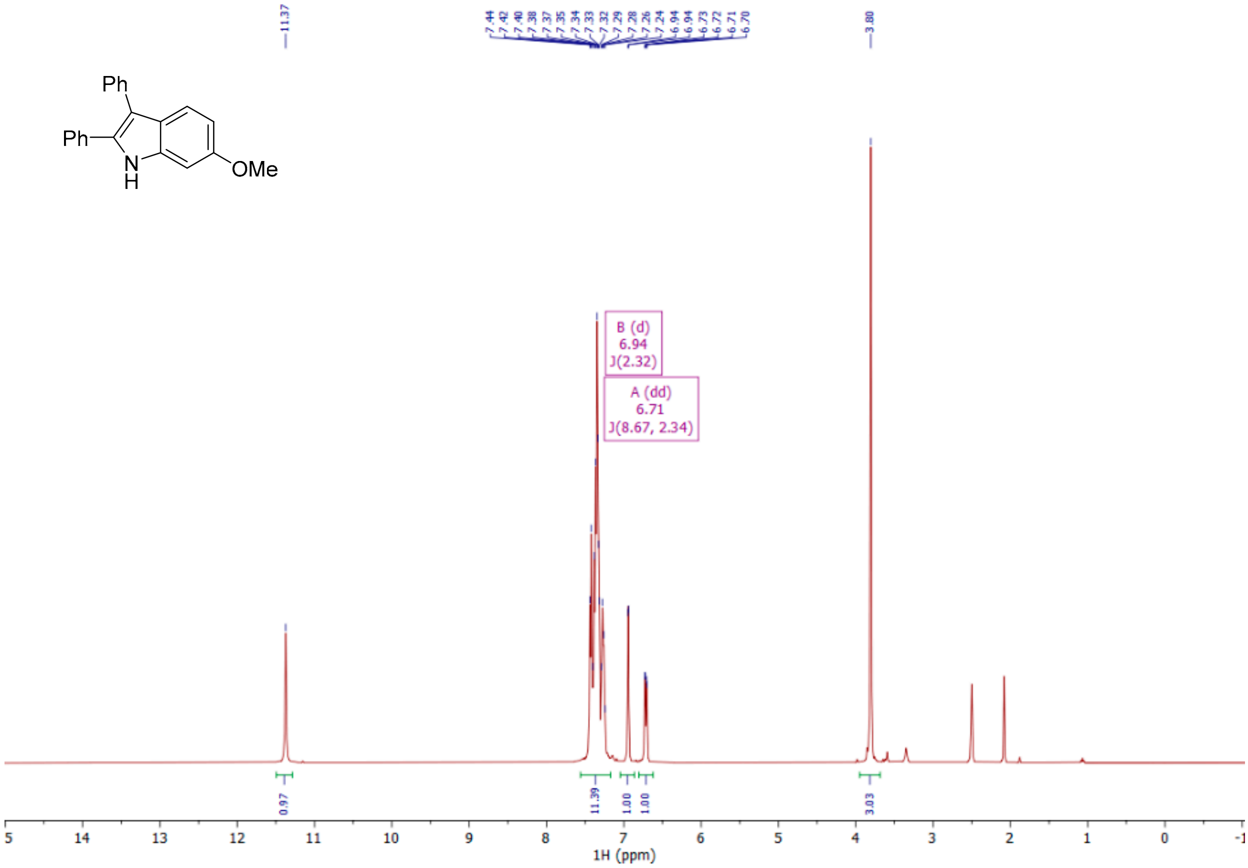

Figure S51. ^{1}H NMR spectrum of 2,3-diphenyl-6-methoxyindole **13**.

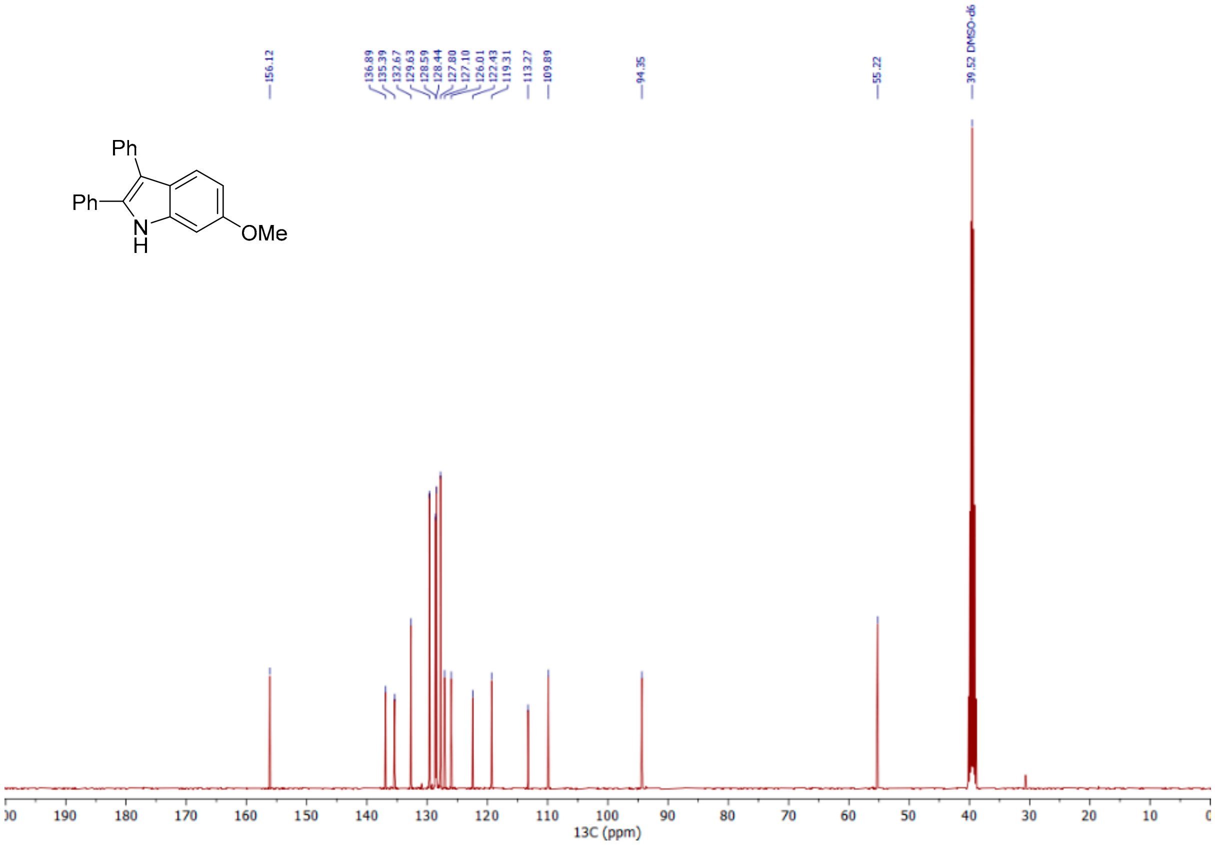

Figure S52. ^{1}H NMR spectrum of 2,3-diphenyl-6-methoxyindole **13**.

Photophysical data

Materials and Methods

Starting materials are commercially available. Water used throughout was Milli-Q water.

Equipment, measurements and characterization methods

UV-Vis absorption spectra were recorded on the spectrophotometer Shimadzu UV-1800. Spectrophotometer using quartz cells with 1 cm path length at room temperature. Emission and excitation spectra were measured on the Horiba FluoroMax-4 using quartz cells with 1 cm path length at room temperature (wavelength range from 350 to 800 nm, slit 2-3 nm, λ_{ex} corresponds to $\lambda_{abs}{}^{max}$). Absolute quantum yields for oxadiazoles were by using the Integrating Sphere Quanta-ϕ of the Horiba-Fluoromax-4. Time-resolved fluorescence measurements were carried out using time-correlated single-photon counting (TCSPC) with a nanosecond LED (370 nm).

Photoluminescence Absolute quantum yield (PLQY) measurement

For each sensor was measured of absorption spectrum so that the concentration of solution was less 0,1 of optical density at selected wavelength to minimize inner-filter effect. For blank (naked solvent) and sensor solution were recorded of emission and Rayleigh scattering spectra by using Integrating Sphere of HORIBA FluoroMax-4. PLQY was calculated by equation: $\phi = (Ec-Ea)/(La-Lc)$, where Ec and Lc are the integrated luminescence of the sensor and blank, Ea and La are the integrated excitation profile of the sensor and blank.

Solvatochromic behavior of pyranoindole compounds

Table S2. Orientation polarizability for solvents (Δf), absorption and fluorescence emission maxima (λ_{abs}, λ_{em}, nm) and Stokes shift (nm, cm^{-1}) of **2** in different solvents.

Solvent	Δf	λ_{abs}, nm	λ_{em}, nm	Stokes shift, nm	Stokes shift, cm^{-1}
n-Heptane	0.0001	335	397	62	4661
Toluene	0.0126	335	401	66	4913
THF	0.21	328	409	81	6038
DCM	0.22	329	410	81	6005
MeCN	0.3	328	420	92	6678

Table S3. Orientation polarizability for solvents (Δf), absorption and fluorescence emission maxima (λ_{abs}, λ_{em}, nm) and Stokes shift (nm, cm^{-1}) of **6b** in different solvents.

Solvent	Δf	λ_{abs}, nm	λ_{em}, nm	Stokes shift, nm	Stokes shift, cm^{-1}
n-Heptane	0.0001	318	424	106	7862
Toluene	0.0126	309	442	133	9738
THF	0.21	304	453	149	10820
DCM	0.22	298	463	165	11959
MeCN	0.3	280	474	194	14617

Table S4. Orientation polarizability for solvents (Δf), absorption and fluorescence emission maxima (λ_{abs}, λ_{em}, nm) and Stokes shift (nm, cm^{-1}) of **7a** in different solvents.

Solvent	Δf	λ_{abs}, nm	λ_{em}, nm	Stokes shift, nm	Stokes shift, cm^{-1}
n-Heptane	0.0001	336	440	104	7034
Toluene	0.0126	340	454	114	7385
THF	0.21	340	477	137	8447
DCM	0.22	340	486	146	8836
MeCN	0.3	335	502	167	9930

Table S5. Orientation polarizability for solvents (Δf), absorption and fluorescence emission maxima (λ_{abs}, λ_{em}, nm) and Stokes shift (nm, cm^{-1}) of **7c** in different solvents.

Solvent	Δf	λ_{abs}, nm	λ_{em}, nm	Stokes shift, nm	Stokes shift, cm^{-1}
n-Heptane	0.0001	307	428	121	9209
Toluene	0.0126	285	439	154	12309
THF	0.21	280	465	185	14209
DCM	0.22	275	478	203	15443
MeCN	0.3	280	499	219	15674

Table S6. Orientation polarizability for solvents (Δf), absorption and fluorescence emission maxima (λ_{abs}, λ_{em}, nm) and Stokes shift (nm, cm^{-1}) of **7e** in different solvents.

Solvent	Δf	λ_{abs}, nm	λ_{em}, nm	Stokes shift, nm	Stokes shift, cm^{-1}
n-Heptane	0.0001	296	438	142	10953
Toluene	0.0126	301	452	151	11099
THF	0.21	306	474	168	11583
DCM	0.22	306	500	194	12680
DMSO	0.276	304	516	212	13514
MeCN	0.3	303	526	223	13992
MeOH	0.31	305	541	236	14302

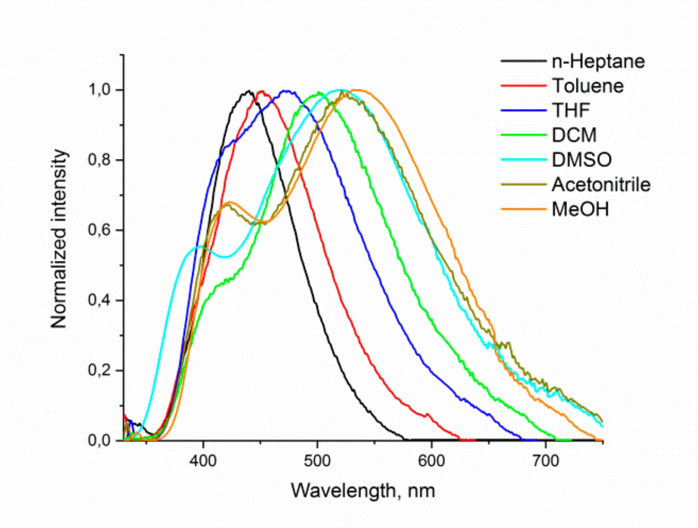

Figure S53. Normalized fluorescence spectra of **7e** in different solvents (C = 10^{-5} M^{-1}).

Table S7. Orientation polarizability for solvents (Δf), absorption and fluorescence emission maxima (λ_{abs}, λ_{em}, nm) and Stokes shift (nm, cm^{-1}) of **8a** in different solvents.

Solvent	Δf	λ_{abs}, nm	λ_{em}, nm	Stokes shift, nm	Stokes shift, cm^{-1}
n-Heptane	0.0001	306	437	131	9796
Toluene	0.0126	301	446	145	10801
THF	0.21	277	462	185	14456
DCM	0.22	276	477	201	15267
MeCN	0.3	287	496	209	14682

Table S8. Orientation polarizability for solvents (Δf), absorption and fluorescence emission maxima (λ_{abs}, λ_{em}, nm) and Stokes shift (nm, cm^{-1}) of **8c** in different solvents.

Solvent	Δf	λ_{abs}, nm	λ_{em}, nm	Stokes shift, nm	Stokes shift, cm^{-1}
n-Heptane	0.0001	306	396	90	7427
Toluene	0.0126	325	432	107	7621
THF	0.21	306	458	152	10846
DCM	0.22	312	478	166	11131
DMF	0.274	318	493	175	11163
MeCN	0.3	300	494	194	13090
MeOH	0.31	302	531	229	14280

Table S9. Orientation polarizability for solvents (Δf), absorption and fluorescence emission maxima (λ_{abs}, λ_{em}, nm) and Stokes shift (nm, cm^{-1}) of **8d** in different solvents.

Solvent	Δf	λ_{abs}, nm	λ_{em}, nm	Stokes shift, nm	Stokes shift, cm^{-1}
n-Heptane	0.0001	308	419	111	8601
Toluene	0.0126	329	441	112	7719
THF	0.21	315	458	143	9912
DCM	0.22	323	470	147	9683
MeCN	0.3	306	489	183	12229

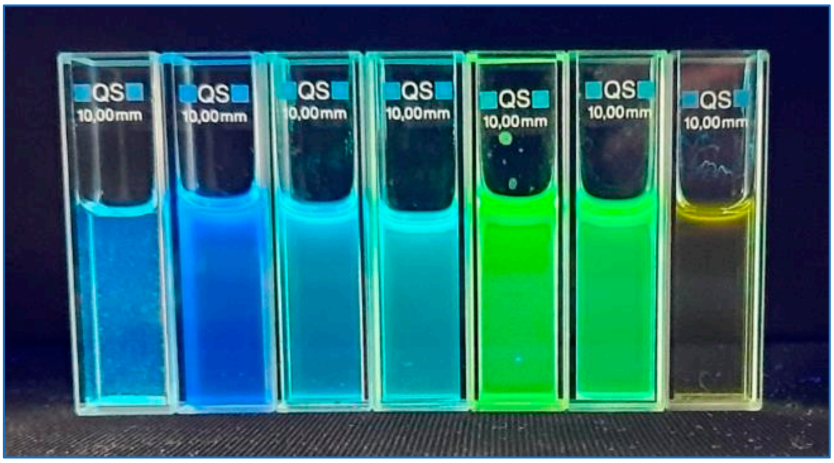

Figure S54. Fluorescence photograph of pyranoindole **7e** (1 mM, excitation with 365 nm Hg lamp) in different solvents (left to right: *n*-heptane, toluene, tetrahydrofuran, dichloromethane, DMSO, acetonitrile, methanol).

Table S10. Orientation polarizability for solvents (Δf), absorption and fluorescence emission maxima (λ_{abs}, λ_{em}, nm) and Stokes shift (nm, cm^{-1}) of **8e** in different solvents.

Solvent	Δf	λ_{abs}, nm	λ_{em}, nm	Stokes shift, nm	Stokes shift, cm^{-1}
n-Heptane	0.0001	308	431	123	9266
Toluene	0.0126	314	448	134	9526
THF	0.21	306	478	172	11759
DCM	0.22	305	499	194	12747
DMSO	0.276	303	523	218	13598
MeCN	0.3	305	524	219	13703
MeOH	0.31	306	530	224	13812

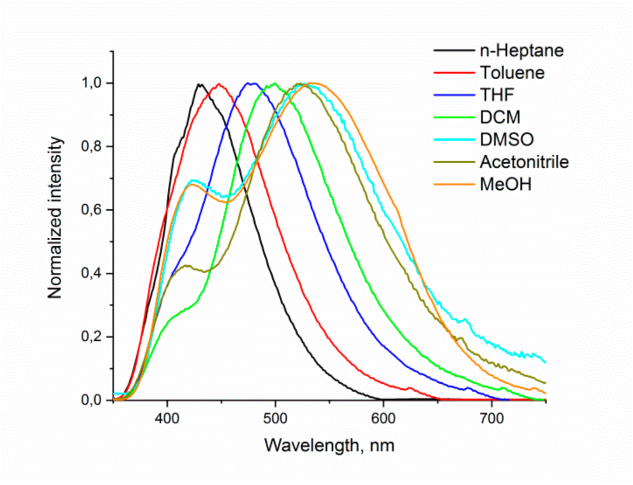

Figure S55. Normalized fluorescence spectra of **8e** in different solvents (C = 10^{-5} M^{-1}).

Table S11. Orientation polarizability for solvents (Δf), absorption and fluorescence emission maxima (λ_{abs}, λ_{em}, nm) and Stokes shift (nm, cm^{-1}) of **8f** in different solvents.

Solvent	Δf	λ_{abs}, nm	λ_{em}, nm	Stokes shift, nm	Stokes shift, cm^{-1}
n-Heptane	0.0001	305	364	59	5314
Toluene	0.0126	323	432	109	7812
THF	0.21	319	449	130	9076
DCM	0.22	321	465	144	9647
MeCN	0.3	294	473	179	12872

Table S12. Orientation polarizability for solvents (Δf), absorption and fluorescence emission maxima (λ_{abs}, λ_{em}, nm) and Stokes shift (nm, cm^{-1}) of **8g** in different solvents.

Solvent	Δf	λ_{abs}, nm	λ_{em}, nm	Stokes shift, nm	Stokes shift, cm^{-1}
n-Heptane	0.0001	320	406	86	6619
Toluene	0.0126	320	426	106	7776
THF	0.21	306	444	138	10157
DCM	0.22	310	459	149	10472
DMF	0.274	318	472	154	10260
MeCN	0.3	304	474	170	11798
MeOH	0.31	305	522	217	13629

Table S13. Orientation polarizability for solvents (Δf), absorption and fluorescence emission maxima (λ_{abs}, λ_{em}, nm) and Stokes shift (nm, cm^{-1}) of **9a** in different solvents.

Solvent	Δf	λ_{abs}, nm	λ_{em}, nm	Stokes shift, nm	Stokes shift, cm^{-1}
n-Heptane	0.0001	332	442	110	7496
Toluene	0.0126	335	455	120	7873
THF	0.21	334	475	141	8888
DCM	0.22	336	499	163	9722
DMF	0.274	337	516	179	10294
MeCN	0.3	334	522	188	10783
MeOH	0.31	339	550	211	11317

Table S14. Orientation polarizability for solvents (Δf), absorption and fluorescence emission maxima (λ_{abs}, λ_{em}, nm) and Stokes shift (nm, cm^{-1}) of **9b** in different solvents.

Solvent	Δf	λ_{abs}, nm	λ_{em}, nm	Stokes shift, nm	Stokes shift, cm^{-1}
n-Heptane	0.0001	335	423	88	6210
Toluene	0.0126	333	429	96	6720
THF	0.21	330	429	99	6993
DCM	0.22	329	432	103	7247
MeCN	0.3	329	435	106	7406

Table S15. Orientation polarizability for solvents (Δf), absorption and fluorescence emission maxima (λ_{abs}, λ_{em}, nm) and Stokes shift (nm, cm^{-1}) of **10** in different solvents.

Solvent	Δf	λ_{abs}, nm	λ_{em}, nm	Stokes shift, nm	Stokes shift, cm^{-1}
n-Heptane	0.0001	330	454	124	8277
Toluene	0.0126	327	460	133	8842
THF	0.21	328	477	149	9523
DCM	0.22	325	499	174	10729
MeCN	0.3	322	516	194	11676

Table S16. Orientation polarizability for solvents (Δf), absorption and fluorescence emission maxima (λ_{abs}, λ_{em}, nm) and Stokes shift (nm, cm^{-1}) of **12** in different solvents.

Solvent	Δf	λ_{abs}, nm	λ_{em}, nm	Stokes shift, nm	Stokes shift, cm^{-1}
n-Heptane	0.0001	320	506	186	11487
Toluene	0.0126	314	504	190	12006
THF	0.21	320	542	222	12799
DCM	0.22	314	563	249	14085
MeCN	0.3	315	593	278	14883

Table S17. Lippert-Mataga plot for compounds **2**, **6**, **7**, **8**, **9**, **10**, and **12**.

compound	Slopes	R^2	Δμ, D
2	7792	0.89	9.9
6b	17884	0.87	15.0
7a	10606	0.87	11.6
7c	21179	0.86	16.4
7e	9806	0.87	11.1
8a	20984	0.89	16.3
**8c**	**19962**	**0.97**	**15.9**
8d	15421	0.89	14.0
**8e**	**14906**	**0.97**	**13.8**
8f	18727	0.87	15.4
**8g**	**17850**	**0.93**	**15.1**
**9a**	**9814**	**0.90**	**11.2**
9b	3879	0.86	7.0
10	11239	0.87	11.9
12	10707	0.89	11.7

Table S18. The photophysical properties of compounds in different solvents.

Compound	*n*-Heptane	Toluene	THF	DCM	DMSO	MeCN	MeOH
**2**	397	401	409	410	424	420	450
**6b**	424	442	453	463	477	474	526
**7a**	440	454	477	486	520	502	553
**7c**	428	439	465	478	505	499	532
**7e**	438	452	474	500	516	526	541
**8a**	437	446	462	477	500	496	531
**8c**	396	432	458	478	501	494	531
**8d**	419	441	458	470	495	489	524
**8e**	431	448	478	499	527	524	530
**8f**	364	432	449	465	485	473	519
**8g**	406	426	444	459	479	474	522
**9a**	442	455	475	499	521	522	550
**9b**	423	429	429	432	450	435	450
**10**	454	460	477	499	525	516	549
**12**	506	504	542	563	598	593	598

Lippert-Mataga equation:

$$\nu_A - \nu_F = \frac{2}{hc}\left(\frac{\varepsilon-1}{2\varepsilon+1} - \frac{n^2-1}{2n^2+1}\right)\frac{(\mu_E-\mu_G)^2}{a^3}$$ (**formula 1**)

ν_A и ν_F – the wavenumbers (cm^{-1}) of the absorption and emission, respectively
h = 6.6256 × 10^{-27} – Planck's constant,
c = 2.9979 × 10^{10} cm/s – speed of light,
a – the radius of the cavity in which the fluorophore resides,
a^3 = $Å^3$ – the van der Waals volume,
ε – Relative permittivity of the solvent,
n – Refractive index of the solvent.
According to Abraham and co-workers, the van der Waals volume ($Å^3$/molecule) can be calculated from the following formula [1]:
V_{vdW} = (all atom contributions) − 5.92N_B; −14.7R_A −3.8R_{NA} (**formula 2**)
where, N_B is the number of bonds, R_A is the number of aromatic rings, and R_{NA} is the number of non-aromatic rings.

Table S19. Fluorescence lifetime of probe 8c, 8g, 9a (C = 2×10^{-6} M) in MeOH.

Compound	τ_1, ns[a]	α_1[b]	τ_2, ns[a]	α_2[b]	τ_3, ns[a]	α_3[b]	τ, ns[a]	χ^2[d]
**8c (420 nm)**	1.190024	34.70	3.754074	65.30	-	-	**2.86**	1.178404
**8c (531 nm)**	3.506346	80.70	6.982067	19.30	-	-	**4.18**	1.118359
**8g (522 nm)**	6.070119	100.00	-	-	-	-	**6.07**	1.062046
**9a (398 nm)**	1.684421	36.47	1.281684	41.96	3.776949	21.57	**1.15**	1.091145
**9a (564 nm)**	0.764980	76.30	4.506832	23.70	-	-	**1.64**	1.163146

[a] Decay time, [b] Fractional contribution, [c] Weighted average decay time $\tau_{av} = \Sigma (\tau_i \times \alpha_i)$, [d] Quality of fitting

Table S20. Energies of HOMO and LUMO, and energy gap for compounds **7** and **8**.

Compound	E(HOMO), eV	E(LUMO), eV	HOMO-LUMO energy gap, eV
**7a**	-6.99	-0.74	6.25
**7b**	-6.93	-0.66	6.27
**7c**	-6.96	-0.70	6.26
**7d**	-6.96	-0.72	6.24
**7e**	-7.10	-0.91	6.19
**7f**	-6.94	-0.65	6.29
**7g**	-6.91	-0.61	6.30
**7h**	-6.92	-0.63	6.29
**8a**	-6.99	-0.76	6.23
**8b**	-6.90	-0.65	6.25
**8c**	-6.96	-0.69	6.27
**8d**	-6.93	-0.71	6.22
**8e**	-7.09	-0.94	6.15
**8f**	-6.91	-0.65	6.26
**8g**	-6.88	-0.59	6.29
**7e_MeCN**	-6.84	-0.81	6.03
**8e_MeCN**	-6.86	-0.81	6.05

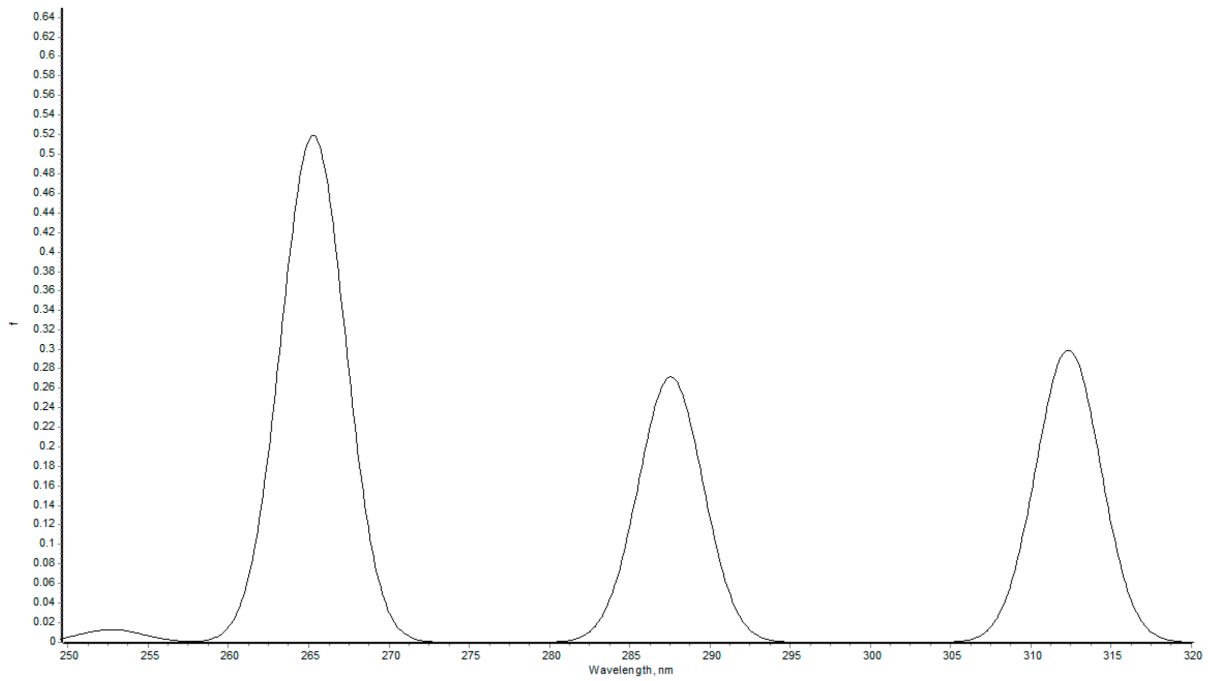

Figure S56. Calculated UV-Vis spectra for optimized equilibrium model structure **7c** (CAM-B3LYP/6-31+G* level of theory).

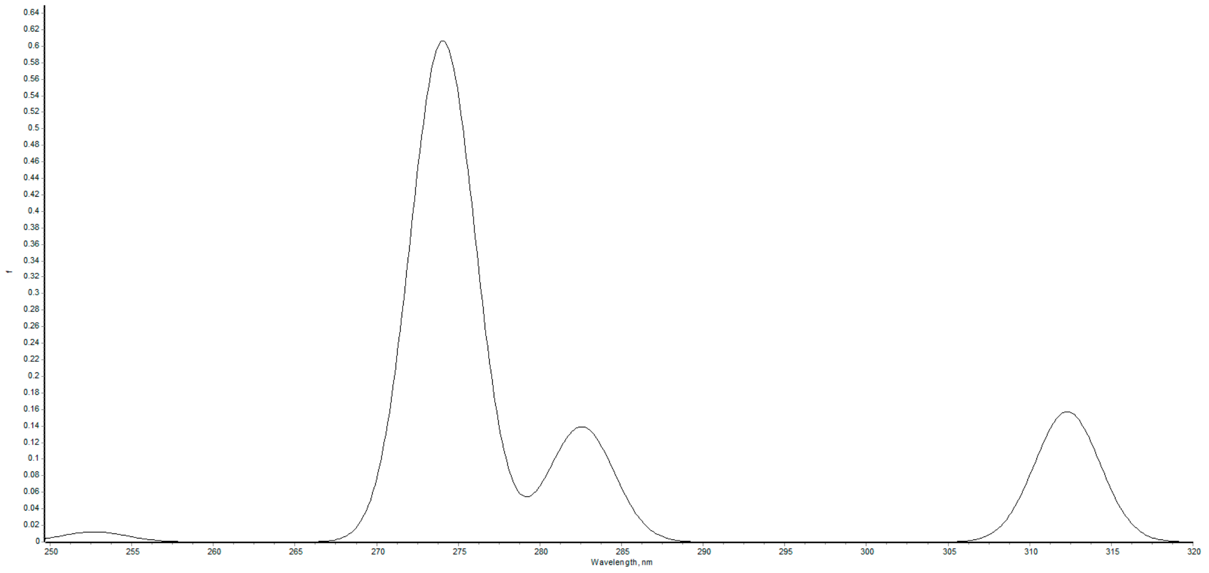

Figure S57. Calculated UV-Vis spectra for optimized equilibrium model structure **8c** (CAM-B3LYP/6-31+G* level of theory).

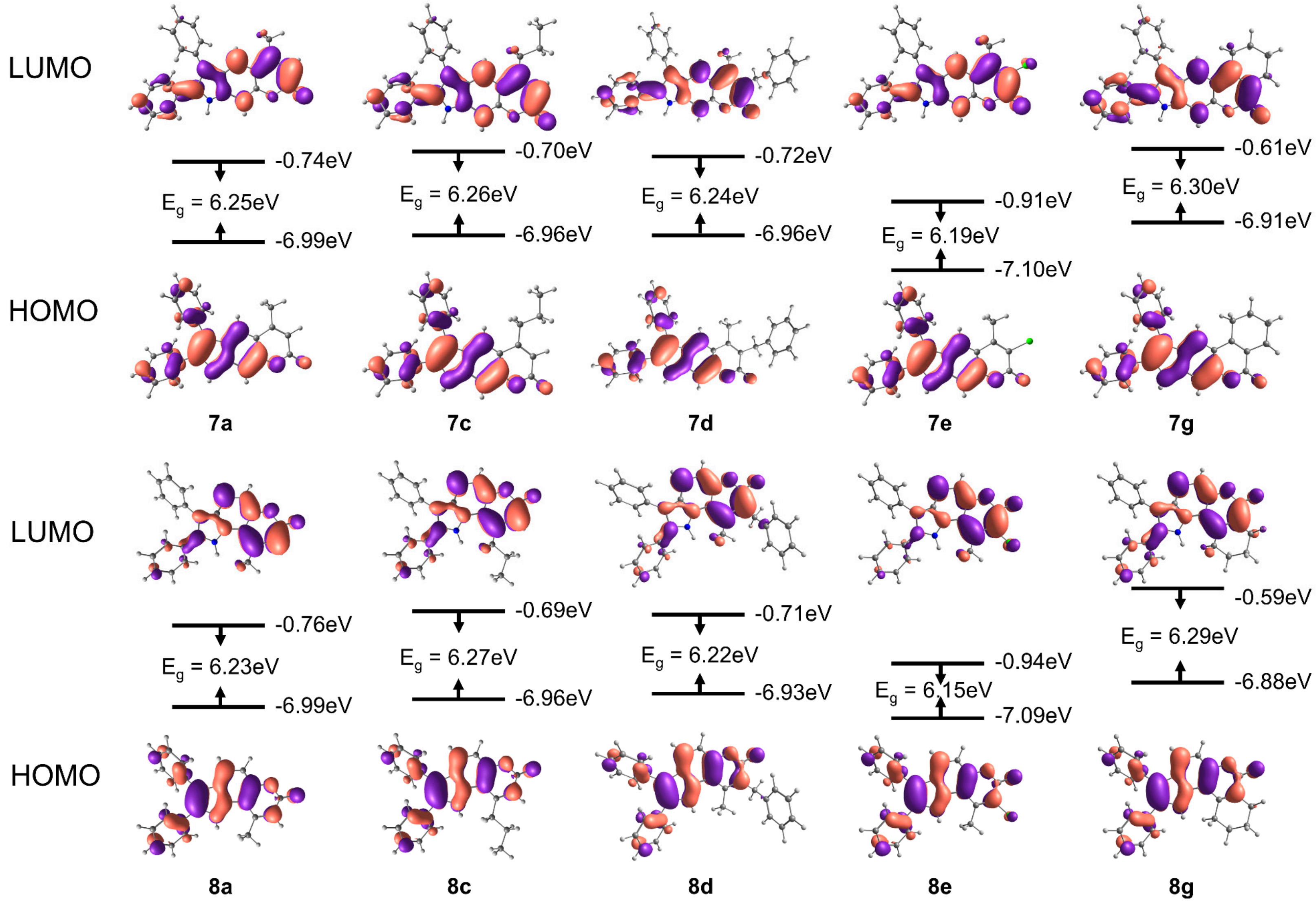

Figure S58. Molecular orbital and energy levels of **7a-8a**, **7c-8c**, **7d-8d**, **7e-8e**, **7g-8g** pyranoindole fluorophores, calculated **at** the CAM-B3LYP/6-31+G* level of theory.

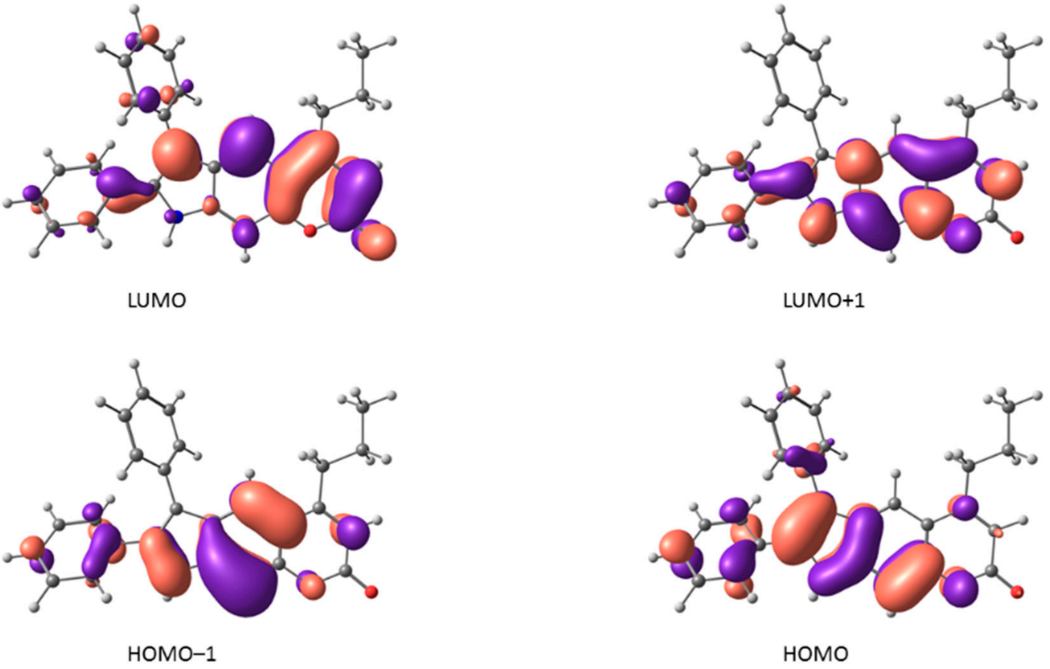

Figure S59. Visualization of HOMOs and LUMOs responsible for observed UV-Vis spectra in optimized equilibrium model structure **7c** (CAM-B3LYP/6-31+G* level of theory).

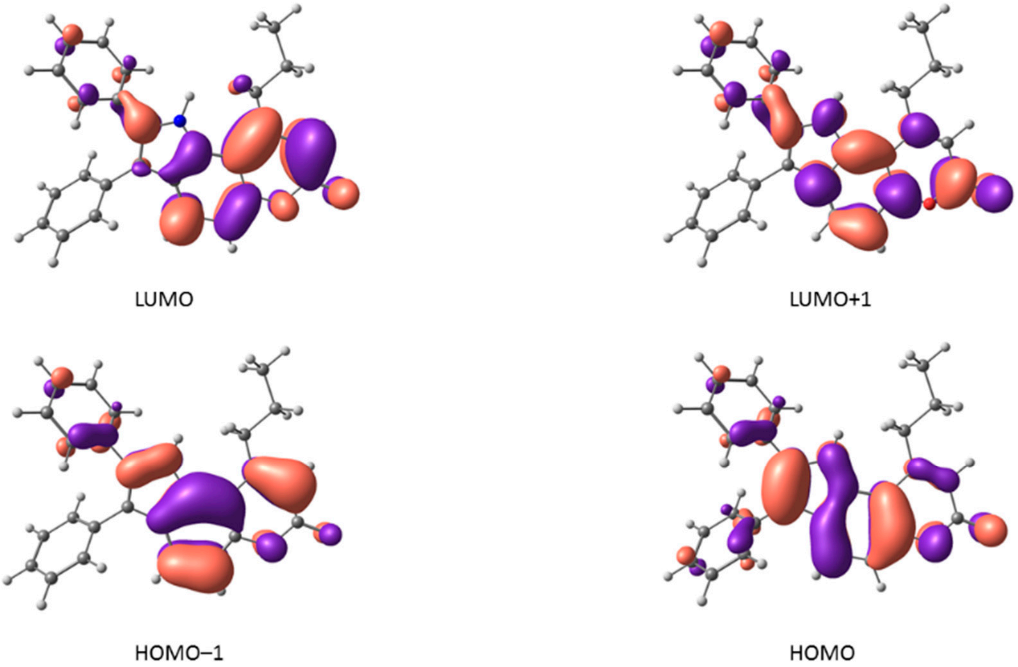

Figure S60. Visualization of HOMOs and LUMOs responsible for observed UV-Vis spectra in optimized equilibrium model structure **8c** (CAM-B3LYP/6-31+G* level of theory).

Table S21. Optimized structures in S_0 in gas phase and visualization of molecular electrostatic potential distribution.

#	Optimized Structures in S_0	Visualization of Molecular Electrostatic Potential Distribution 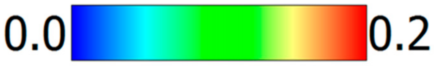
**7a**	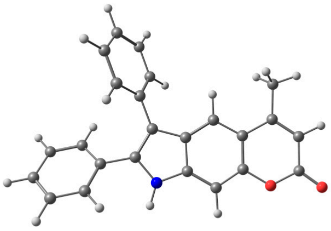	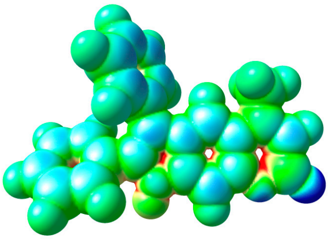
**7c**	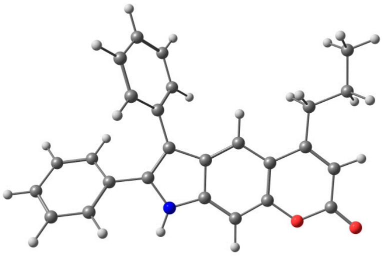	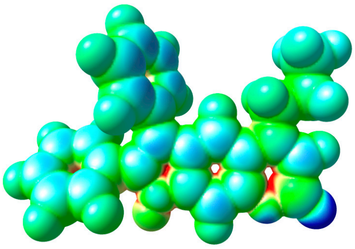
**7d**	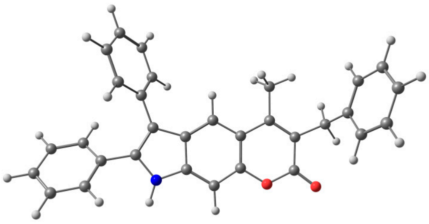	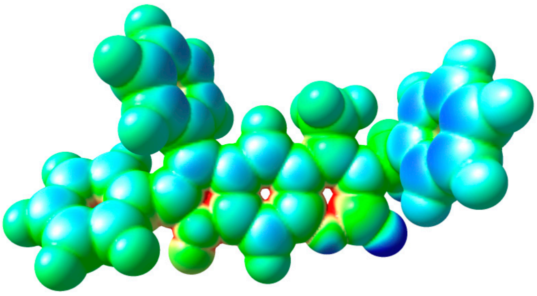
**7e**	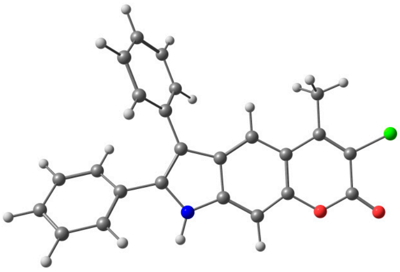	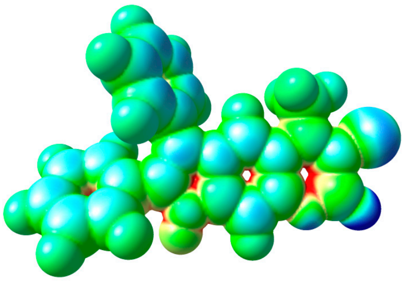
**7g**	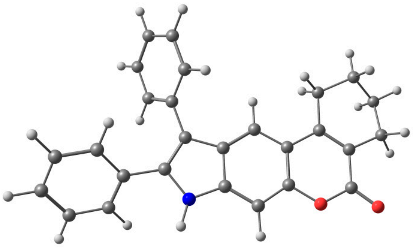	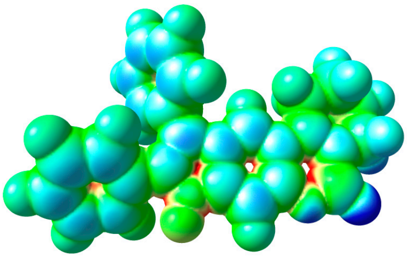
**8a**	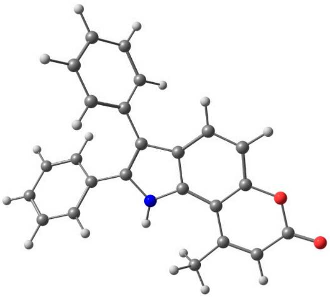	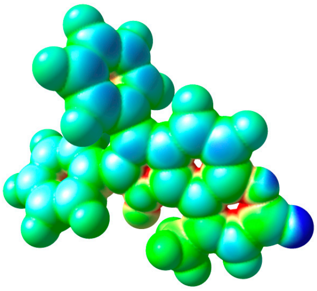

**8c**	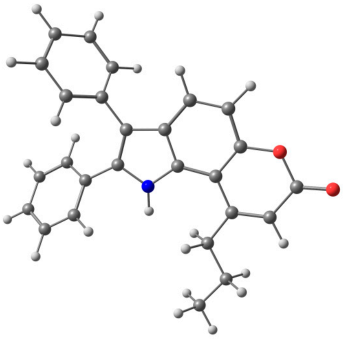	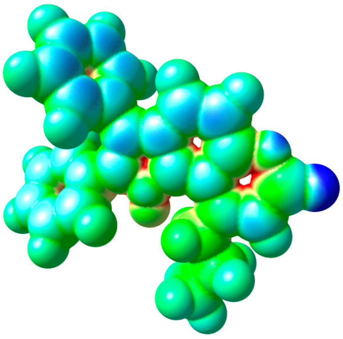
**8d**	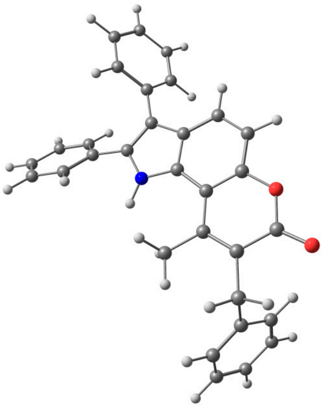	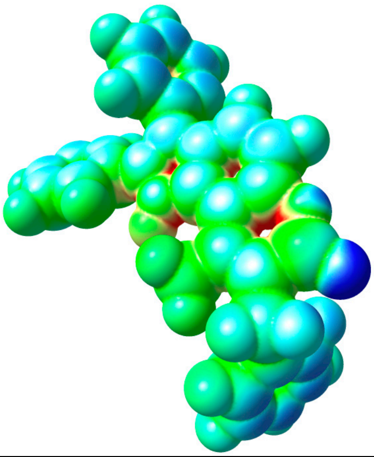
**8e**	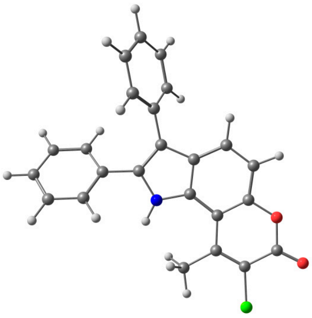	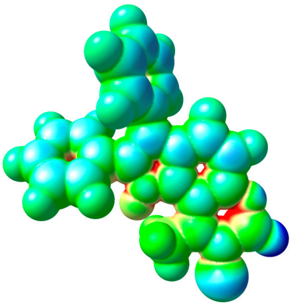
**8g**	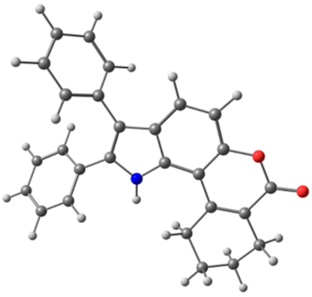	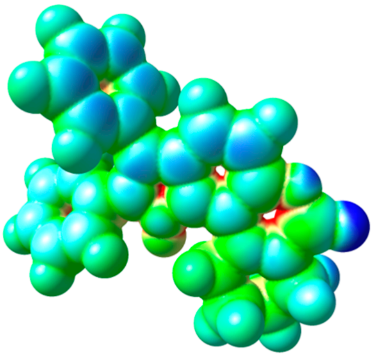
**9a**	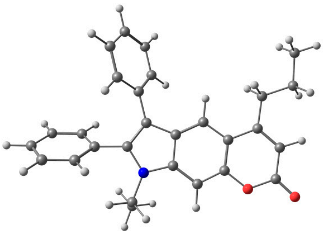	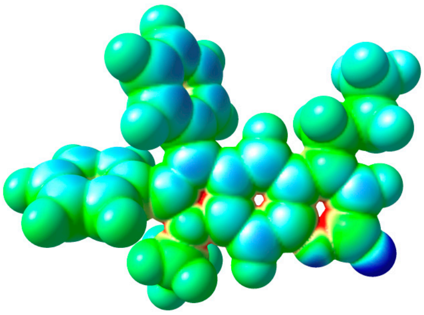

Table S22. Calculated indexes related to hole-electron distribution in model structures.

Model structures	D_{CT}(Å)	S_r (a.u.)
**7a**	1.388	0.75613
**8a**	1.446	0.77065
**7b**	1.269	0.76332
**8b**	1.298	0.78534
**7c**	1.344	0.75773
**8c**	1.363	0.77740
